# USP24 upregulation stabilizes PKA-Cα to promote lipogenesis, inflammation, and fibrosis during MASH progression

**DOI:** 10.1186/s12929-025-01148-4

**Published:** 2025-05-30

**Authors:** Beh Ning, Shao-An Wang, Ming-Jer Young, Yung-Ching Chen, Yun Hung, Tran Thu Huong, Wen-Chang Chang, Yi-Ching Wang, Ming-Lung Yu, Kai-Cheng Hsu, Jan-Jong Hung

**Affiliations:** 1 Department of Biotechnology and Bioindustry Sciences, National Cheng Kung University, Tainan, 701 Taiwan; 2 School of Respiratory Therapy, College of Medicine, Taipei Medical University, Taipei, Taiwan; 3 Center of Excellence for Metabolic Associated Fatty Liver Disease, National Sun Yat-Sen University, Kaohsiung, Taiwan; 4 Graduate Institute of Medical Sciences, College of Medicine, Taipei Medical University, Taipei, Taiwan; 5 Graduate Institute of Cancer Biology and Drug Discovery, College of Medical Science and Technology, Taipei Medical University, Taipei, Taiwan; 6 Institute of Pharmacology, National Cheng Kung University, Tainan, Taiwan

**Keywords:** USP24, Lipogenesis, p-CREB, PKA-Cα, MASH

## Abstract

**Background:**

Ubiquitin-specific peptidase 24 (USP24), a deubiquitinating enzyme, regulates protein stability by removing ubiquitin. This study investigates the role of UPS24 in lipid metabolism, inflammation, and fibrosis. It also explores the effect of targeting USP24 on metabolic disorders, focusing on high-fat diet (HFD)-induced obesity and liver diseases.

**Methods:**

This study utilized CRISPR/Cas9 to create functional knockout mice (USP24^C1695A^) and treated HFD-fed mice with USP24 inhibitor (USP24-i-101). The effects of USP24 inhibition or knockout on 3T3-L1 derived adipocytes, primary hepatocytes, hepatic stellate cells, and murine hepatocyte cell line AML12 (alpha mouse liver 12) cells were assessed with RNA-sequencing. Molecular mechanisms and the interaction between USP24 and PKA-Cα were studied with co-immunoprecipitation. Downstream signaling pathways involving CREB, SREBP1, PPARγ, and C/EBPβ, as well as USP24 role in liver inflammation and fibrosis, were studied using western blot and real-time PCR. Clinical and animal tissue samples were examined with immunohistochemistry to identify the correlations between USP24 and metabolic-associated liver diseases.

**Results:**

Knockout or inhibition of USP24 reduced body weight, lipid accumulation, inflammation, and fibrosis in HFD-fed mice. The expression of genes related to lipogenesis, inflammation, and fibrosis was downregulated in USP24^C1695A^ mice and those treated with USP24 inhibitor (USP24-i-101). USP24 inhibition decreased lipid droplet accumulation in adipocytes and hepatocytes, suppressed inflammation in hepatocytes and AML12 cells, and reduced fibrosis in hepatic stellate cells. Mechanistically, USP24 expression was upregulated by PKA activation during adipocyte differentiation, leading to increased PKA-Cα stability and CREB phosphorylation, which promoted lipogenic gene expression. Free fatty acids (FFA) increased USP24 expression, activating NF-κB and TGFβ pathways to induce inflammation (Cox2) and fibrosis (α-SMA). USP24 was highly expressed in patients with metabolic dysfunction-associated steatohepatitis (MASH) and correlated with Cox2 and α-SMA levels.

**Supplementary Information:**

The online version contains supplementary material available at 10.1186/s12929-025-01148-4.

## Introduction

Deubiquitinases (DUBs) are specific enzymes that regulate multiple cellular functions by modulating ubiquitin molecules. Ubiquitin-specific peptidases belong to the superfamily of DUBs associated with various human diseases, including cancer progression [1]. More than 50 ubiquitin-specific peptidases (USPs) have been identified, and most of these enzymes exert their functions by reversing the polyubiquitination or monoubiquitination of target proteins. Malfunction of the ubiquitin system can either enhance the effect of oncogenes or reduce the activity of tumor suppressor genes, and this system has been implicated in the tumorigenesis of various cancers [2, 3]. USP24 is a 2620-amino acid protein containing one ubiquitin-associated domain (UBA), which binds to the ubiquitin signal on substrate proteins, and one ubiquitin C-terminal hydrolase (UCH) domain, which is the catalytic domain. The function of USP24 is poorly understood, and most studies examining USP24 have focused on the single nucleotide polymorphisms (SNPs) of USP24 implicated in Parkinson’s disease (PD) [4, 5]. In our previous study, we demonstrated that USP24 expression was upregulated in most late-stage lung cancer patients due to increased mRNA stability caused by SNPs or RNA editing [6]. Upregulation of USP24 decreases the stability of the methyltransferase Suv39h1 by promoting the expression of MDM2. The downregulation of Suv39h1 releases downstream genes from inhibition, leading to the expression of metastasis-related genes, such as those encoding CCL5 and ADAM10 [6]. Based on these findings, the upregulation of USP24 in cancer cells plays a critical role in promoting lung cancer metastasis. Recently, we used a structure modelling method to screen a novel specific USP24 inhibitor, USP24-i-101, which can inhibit drug resistance during lung cancer therapy in autophagy activation dependent manner [7, 8]. However, there is no study about the role of USP24 in lipogenesis. 

Lipids derived from food or de novo lipogenesis are an important energy source. Imbalance in the synthesis or degradation of fat storage is associated with various diseases, such as obesity, diabetes, and lipodystrophy [9]. In mammals, excess fatty acids are stored in cytosolic lipid droplets (LDs) [10]. Several critical regulatory factors, including sterol regulatory element-binding proteins (SREBPs), function as sensors to control lipid homeostasis [11]. Additionally, mTORC1 and mTORC2 have been implicated in cancer lipid biogenesis through SREBP1-dependent and SREBP1-independent mechanisms [12]. Obesity-driven LD accumulation in non-adipose tissues is linked to insulin resistance, cardiovascular disease, and cancer [13]. Previous studies have shown that lipid droplets are involved in cancer-related signaling pathways, tumor-immune cell crosstalk, eicosanoid synthesis, cell cycle progression, sequestration of hydrophobic therapeutic agents, hypoxia-mediated lipid metabolism alterations, and the epithelial mesenchymal transition [14, 15]. However, the role of LDs in genomic stability remains poorly understood and required further investigation. Research has indicated that lipid droplets serve as the substrate for autophagy [16]. Multipotent stem cells (MSCs) differentiate into adipocytes through two stages, the commitment stage and the differentiation stage, when related factors like MDIR induction medium (M: IBMX, D: dexamethasone, I: Insulin, R: rosiglitazone) are added. Free fatty acids are released from mature adipocytes enter the circulation and induce fatty liver, metabolic associated steatohepatitis (MASH), vascular-related diseases, and diabetes [17, 18]. Proteins like C/EBPs and PPARγ are known to be directly involved in adipocyte differentiation [19]. C/EBPβ and C/EBPδ are upregulated during the early period to enhance PPARγ expression, while PPARγ combines with C/EBPα to activate adipocyte differentiation during the late period [20]. PKA or other kinases like AKT and MAPK phosphorylate CREB to activate lipogenesis-related genes [21]. Furthermore, acetylation of CREB increases transcription activity by recruiting p300/CBP [22]. MAFLD progresses to MASH, which increases the risk of developing liver failure, cirrhosis, and hepatocellular carcinoma [23]. In the livers of MASH patients, three major phenomena—lipogenesis in hepatocytes, inflammation in macrophages, and fibrosis in hepatic stellate cells (HSCs)—can be observed [23]. This study found that functional knockout of USP24 or USP24-i-101 targeting USP24 resulted in similar phenotypes, including weight loss in mice and inhibited MASH progression. 

## Materials and methods

### Cell culture and transfection

3T3-L1 mouse preadipocyte fibroblast cells and AML12 normal mouse hepatocytes were obtained from the Bioresource Collection and Research Center (BCRC), Taiwan. Mice primary hepatocytes were isolated from C57BL/6Jnarl mice obtained from Taiwan National Applied Research Laboratories (NARlabs). Huh7 was kindly provided by Professor Wen-Ya Huang from NCKU in Taiwan. LX-2 was kindly provided by Professor Su-Chi Wang from Kaohsiung Medical University. 3T3-L1 cells were cultured in DMEM (Corning, NY, US) containing 10% FBS, and 100U/mL penicillin/streptomycin antibiotics. AML12, Huh7, LX-2 cells and mouse primary hepatocyte cells were cultured in Ham’s F12/DMEM medium (Corning, NY, US) containing 10% FBS, 100 units/mL penicillin/streptomycin, 40 ng/mL dexamethasone (MedChemExpress, NJ, US) and Insulin–Transferrin–Sodium Selenite supplement (Roche, Basel, CH). Cells were maintained in a sterile environment and incubated in a humid incubator at 37 °C with 5% CO2.

### Animal experiment

C57BL/6Jnarl mice obtained from Taiwan National Applied Research Laboratories (NARlabs) were employed for animal experiments. Mice approximately 8–10 months of age were confined in standard cages under a 12-h light/dark cycle at 25 °C. Mice were fed a normal chow diet and high-fat diet (Research Diets, New Brunswick, NJ, USA) for 6 months to model obesity and fatty liver. The normal chow diet comprises 13.1% fat, 58.2% carbohydrate, and 28.6% protein as calorie intake, while the high-fat diet contains 60% fat, 20% carbohydrate, and 20% protein as calorie intake. Food intake and body weight were measured weekly, and blood sugar were determined in fasted mice via tai sampling. Additionally, USP24^C1695^^A^ functional knockout mice were constructed using CRISPR/Cas-9 genome editing (Suppl. Figure 1). The designed sgRNA guided Cas-9 and recognized the USP24 sequence through its 5’crRNA complementary base pair component. Various dosage of USP24 inhibitors were administered intraperitoneally. (IACUC Approval Number: 109110, 111124, 111171).

### Reverse transcription-quantitative polymerase chain reaction (RT-qPCR)

A reverse transcription pre-mixture (RT-premix) was prepared by combining 3 μg of RNA, 10 mM dNTP, and 20 mM OligoDT, with MQ water to a total volume of 14 μL. The reverse transcription buffer mixture (RT-buffer) consisted of 4 μL of 5X First Strand Buffer, 0.1 M dithiothreitol, 0.5 μL Superscript II, and 0.5 μL RNAse OUT (Invitrogen, MA, US). The RT-premix was incubated at 65 °C for 5 min, followed by 4 °C for 2 min. Subsequently, 6 μL of RT-buffer were added into RT-premix, which was then continued at 25 °C for 15 min, 50 °C for 50 min, and 72 °C for 15 min using C1000^™^ Thermo Cycler. The resulting cDNA was stored at −20 °C until use. Quantitative PCR was carried out using KAPA SYBR green reagent (Kapabioscience, NC, US) on a Bio-Rad CFX Connect Real-Time PCR detection system. Results were normalized and quantified using ∆∆Ct method. The sequences of the primers used are listed in Supplementary Table 4.

### Ultrasound assay

Ultrasound data were generated and recorded using a VisualSonics Vevo 770 (Laboratory Animal Center, College of Medicine, National Cheng Kung University) with a RMV704 probe (40-MHz center frequency). Hepatorenal echo contrast is the difference of echo between the liver and kidney. Bright liver indicates stronger and more intense echoes from the hepatic parenchyma.

### Magnetic resonance imaging (MRI)

MRI was carried out on BURKER 7 T PharmaScan (Laboratory Animal Center, Taipei Medical University, Taiwan) to detect body fat in mice. The field strength was 7 T and coil diameter was 40 mm. A spin echo sequence (SE_FAT, TR = 1000 ms, TE = 16 ms) was employed. Each mouse was imaged for a total of 16 slices at a thickness of 1.0 mm, with a field of view of 40 × 40 mm and matrix size of 300 × 400 mm.

### Rotarod test

The rotarod test was performed as described previously [24]. USP24^WT^ and USP24^C1695A^ mice were placed in a rotating rod and the latency to fall from a rotating rod was scored automatically with infrared sensors in a Rotamex 5 rotarod (Columbus Inst, Columbus, OH, USA).

### Biochemical tests

Blood samples were collected via terminal cardiocentesis from non-fasted, anesthetized mice. Samples were centrifuged for 15 min at 3000 rpm to extract the serum. The serum samples were then frozen at −80 °C and stored until further analysis. The levels of total protein (TP), glutamic oxaloacetic transaminase (GPT), glutamic oxaloacetic transaminase (GOT), albumin (ALB), alanine aminotransferase (ALT), triglyceride (TG) and total cholesterol (TCHO) in the serum were determined using FUJI DRI-CHEM 4000i (Laboratory Animal Center, College of Medicine, National Cheng Kung University).

### ImageJ quantification

Western blot results were analyzed using the Gel Analyzer function. Oil Red O staining was measured through the color threshold function. Adipocyte cell size and number were assessed with the Adiposoft plugin. ImageJ (F.I.J.I.) version 1.53o was employed for all analyses.

### Lentivirus infection knockdown

3T3-L1 cells were seeded in 6 well plates at a density of 15000 cells/well and incubated at 37 °C for 24 h. Lentivirus containing shRNA targeting mouse USP24 were added at a M.O.I. of 100 or 200, and cells were cultured for an additional 24 h. The medium was discarded, and fresh medium containing new lentivirus was introduced for another 48 h. Cells were collected to determine knockdown efficiency via western blotting. Lentiviruses were obtained from RNAi-Core, Sinica Academica Taiwan. The target sequence of the mouse USP24 shRNA was 5’-CCCGAGCTCTTGTCTGCCATT-3’.

### Western blotting

Cells were washed with PBS and lysed with sample buffer. For animal tissues, organ tissues placed in RIPA lysis buffer and homogenized using zirconium oxide beads before being lysed with an equal volume of sample buffer after homogenization. Lysed samples were incubated at 95 °C for 10 min prior to use. Protein concentrations were measure using the Pierce™ BCA protein assay kit (Thermo Scientific, MA, US). Proteins were separated on a 10% SDS-PAGE. They were then transferred from the polyacrylamide gel to a PVDF membrane using a wet transfer tank for 3 h at 375 mA. A 5% non-fat milk solution in TBST (10 mM Tris–HCl, 150 mM NaCl, and 0.5% Tween 20, pH 8.0) was used as the blocking buffer, which was applied to the membranes and incubated at room temperature for 1–2 h. Specific primary antibodies for the protein of interest were then incubated with the blotted membranes overnight at 4 °C. The membranes were washed three times with TBST for 10 min each before being incubated with a secondary antibody against rabbit or mouse at room temperature for 1 to 2 h. Proteins of interest were detected using Immobilon^®^ Western Chemiluminescent HRP substrate (Merck Millipore, MA, US) with the UVP CHEMIDOC-IT 815 Imaging System. β-actin and α-tubulin were used as internal controls. If stripping was required, blotted membrane were incubated in stripping buffer (0.1% glycine, 0.1% SDS, 1% Tween 20, pH 2.2). The primary antibodies used in this study are listed in Suppl. Table 5.

### RNA extraction and RNA-sequencing

Cells were washed in cold PBS and lysed with TRIzol^™^ Reagent (Invitrogen, MA, US). Animal organ tissues were placed in cold TRIzol^™^ Reagent and homogenized. Lysed samples were stored in −80 °C until extraction. For 1 mL of sample in TRIzol^™^ Reagent, 200 μL of chloroform was added. Samples were vortexed vigorously for 15 s, incubated at room temperature for 3 min, then centrifuged at 12000 rpm at 4 °C for 15 min. The uppermost aqueous layer (around 450 μL) was extracted. 350 μL of isopropanol was added, samples were vortex gently and incubated at room temperature for 10 min. Samples were then centrifuged at 12000 rpm at 4 °C for 10 min. The supernatant was removed and RNA pellets were washed with 75% ethanol. RNA pellets were air dried and reconstituted in MQ water. RNA quality was measured with OD260/280, with a ratio of 1.8–2.0 desired. RNA samples were stored at −80 °C until use. RNA samples were sent to BIOTOOLS Co., Ltd for RNA sequencing. RNA was sequenced with Illumina NovaSeq 6000, paired-end at 150 bp. Sequencing reads were analyzed using DESeq2, |Fold-Change|> 2 and adjusted p-value of less than 0.05.

### Oil Red O staining

Dissolve 0.5 g of Oil Red O dye (Abcam, CB, UK) in 100 mL isopropanol to prepare a stock solution. A working solution was prepared by diluting stock solution with water to obtain a 60% Oil Red O isopropanol solution, filter the solution. Cells were fixed in 10% formaldehyde/PBS for 30 min. Cells were later incubated in 60% isopropanol for 5 min and stained for 20 min. Cells were washed twice with water and covered with 90% glycerol/PBS. For tissue sections, animal tissues were incubated in 15% sucrose/PBS 6 h at 4 °C, followed by incubation in 30% sucrose/PBS overnight at 4 °C. After drying, tissues were fixed in OCT (Sakura, CA, US) and frozen in liquid nitrogen. Frozen sections were cut at 10–15 µm and air-dried for 30 min. Sections were fixed with 10% formaldehyde/PBS for 5 min, dipped in 60% isopropanol, and stained with the working stain solution for 15 min. Sections were dipped in 60% isopropanol and rinsed with water before being stained with hematoxylin for 1 min. After rinsing with water, 90% glycerol/PBS was used to mount the cover slip.

### 3T3-L1 adipocyte differentiation

3T3-L1 cells were seeded in 6-well plates and grown until confluence. The cells were subsequently fed with fresh DMEM medium for an additional 48 h. The cells were later treated with a differentiation medium consisting of DMEM containing 10% FBS, 500 µM IBMX, 1 µM dexamethasone, 1 µM rosiglitazone (MedChemExpress, NJ, US), and 1.5 µg/mL insulin (Eli Lilly and Company, IN, US) for 48 h. The differentiation medium was replaced with an insulin medium containing 1.5 µg/mL insulin for another 48 h. The cells were then incubated in fresh DMEM medium for an additional 4 days. The cells were stained with Oil Red O stain to confirm lipid accumulation in mature adipocytes.

### Free fatty acid supplement

Free fatty acid (FFA) solution was prepared by dissolving sodium oleate and sodium palmitate (Sigma-Aldrich, MO, US) at 200 mM and 100 mM, respectively, in 50% alcohol at 60 °C for 30 min. The FFA solution was then diluted 10X with a 5 mM BSA solution. The FFA-BSA solution was incubated at 37 °C for 1 h to allow conjugation. Hepatocytes were seeded in 6-well plates at 70% confluency. The cells were treated with medium containing 10% FBS and FFA for 24 h. The cells were then stained with Oil Red O staining to confirm lipid accumulation.

### Primary hepatocyte isolation

Primary mouse hepatocytes were isolated using a collagenase perfusion method [25]. Mice were anaesthetized with 0.2 mL of a 1:1 mixture of Zoletil and Xylazine (Virbac, Carros, FR). The liver was first perfused with a warm perfusion buffer (0.5 mM EDTA, 25 mM HEPES in HBSS, pH 7.4) through the vena cava at a flow rate of 3 mL/min using a peristaltic pump after confirming swelling of the liver. The portal vein was then cut to release the blood. After the blood had cleared from the liver, 20 mL of collagenase (C5138, Sigma-Aldrich, MO, US) in digestion buffer (25 mM HEPES, 0.1 µg/mL collagenase, in HBSS with Ca^2+^ and Mg^2+^, pH 7.4) was perfused into the liver, and the portal vein was clamped every minute to ensure full perfusion of the liver. Next, the liver was dissected and placed in ice-cold digestion buffer. Hepatocytes were released onto an uncoated plate by puncturing the liver sac and gently scraping the liver. The medium was collected and centrifuged at 50 g for 2 min. The cells were resuspended with 20 mL of 45% Percoll solution in DMEM and centrifuged at 200 g for 10 min to obtain purified live hepatocytes. Primary hepatocytes were washed with DMEM and plated on collagen-coated plates with low glucose DMEM medium for 3–4 h, after which the medium was changed to Ham’s DMEM/F12 supplemented with FBS, insulin, transferrin, selenium, and dexamethasone for maintenance. Experiments on primary hepatocytes were conducted within 3 days of isolation.

### Immunohistochemistry

Mouse liver tissues were fixed, dehydrate, and embedded in paraffin after incubating in 10% formaldehyde for 24 h at 5 µm thickness. Sections were stained with Hematoxylin and eosin. The Novolink Polymer Detection Systems (Leica Biosystem, HE, DE) was used for immunohistochemistry. Paraffin-embedded sections were de-paraffinized at 60 °C for 1 h, dewaxed with xylene, and dehydrated with a graded series of ethanol. Endogenous peroxidase was neutralized with Peroxidase Block for 5 min and protein blocking was performed with Protein Block for 5 min. Primary antibodies covered the sections overnight at 4 °C. After washing the primary antibodies, sections were incubated with Post Primary for 30 min, followed by Novolink Polymer for 30 min. Sections were developed using DAB solution for 5 min and counterstained with Hematoxylin. Photographs were taken using an Olympus BX-51 microscope (Olympus, Melville, NY, USA). Primary Antibodies used: anti-USP24 (1:100), anti-PLIN2 (1:200), anti-C/EBPβ (1:200), anti-PPAR-γ (1:500), and anti-SREBP1 (1:500).

### Masson’s trichrome staining

The liver tissues from all mice were collected and routinely fixed in 4% formalin at 4 °C for 48 h before being embedded in paraffin. Sections of 5 µm thickness were cut and then deparaffinized. Masson's trichrome staining was conducted using Masson's trichrome kits (Abcam, ab150686) according to the manufacturer' instruction to measure the density of collagen fibers.

### Protein modeling

A homology model of USP24 was generated using the Modeller module in Chimera, as previously reported [7, 26]. The crystal structures for PKA-Cα (PDB ID: 4WB5) and ubiquitin-USP7 complex (PDB ID: 5KYE) were obtained from The Protein Data Bank [27]. Next, the 3D coordinates of ubiquitin for USP24 were obtained from the ubiquitin-USP7 complex structure, which was used to align the USP24 structure. Protein–protein docking was performed using ZDOCK [28]. Docking results were selected based on the distance between the PKA-Cα residue G76 and Ubiquitin residue K310. The PKA-Cα and ubiquitin structures were then covalently bonded at these residues. Finally, a Molecular Dynamic simulation was performed using Discovery Studio [29]. The simulation was performed for 10 ns at default settings.

### Statistical assay

The investigator was aware of the sample allocation during the experiment and when evaluating its outcome for all experiments. For all experiments, at least three independent biological replicates of each condition were analyzed. The estimated variation within each experiment group is similar. Statistical significance between two experimental groups was calculated using a two-tailed Student’s t-test, where a p-value of less than 0.05 was considered significant. The data and error bars shown represent the mean ± standard deviation and p-values (*p < 0.05; **p < 0.01; ***p < 0.001; ****p < 0.0001). All data were statistically analyzed using GraphPad Prism software version 9.0.

## Results

### USP24 positively regulates adipogenesis

**Fig. 1:**
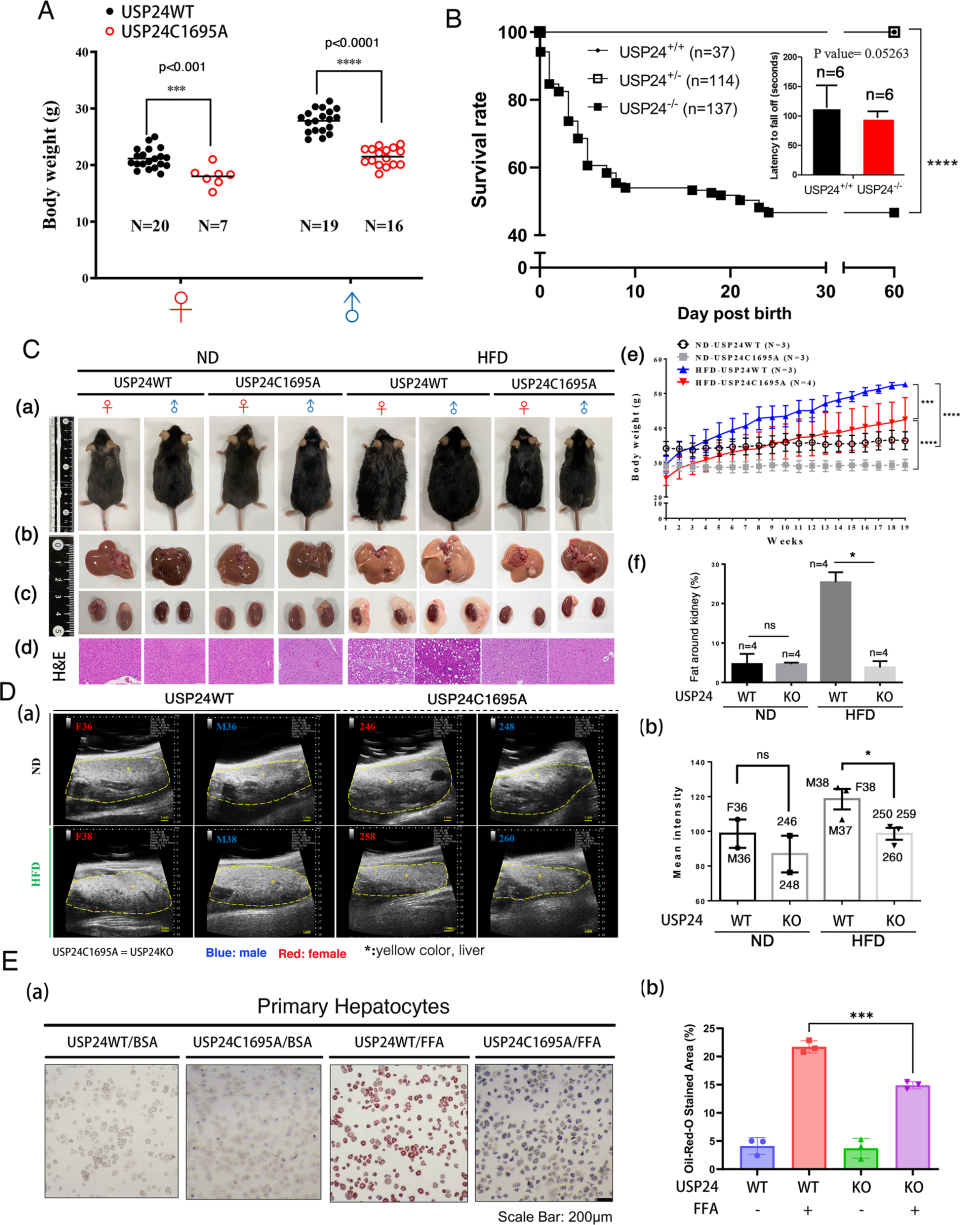
Functional knockout of USP24 suppresses lipogenesis. **A** Body weights of USP24^WT^ and USP24^C1695A^ mice fed a normal diet were measured. **B** Survival rate of newborn mice (**B**, left panel) and physiological activities of mice (**B**, right panel) with or without USP24 knockout were assessed by tracing the survival rate and performing a rotarod test, respectively. The results of **A** and **B** were analyzed by statistical assay, t-test, ***p < 0.001, ****p < 0.0001. USP24^WT^ (n = 3) and USP24^C1695A^ (n = 3) mice and USP24^WT^ (n = 3) and USP24^C1695A^ (n = 4) mice were fed a normal diet (ND, 13.1% fat) or a high-fat-diet (HFD, 60% fat) for 4 months, respectively. Mouse body sizes (**C**, **a**), organs (liver and kidney) (**C**, **b**, **c**), and H&E staining (**C**, **d**) are shown. The body weight of mice (**C**, **e**) and fat around kidneys (**C**, **f**) were quantified, and statistical analysis was performed using a t-test, *p < 0.05, ***p < 0.001, ****p < 0.0001. Fat content inside ND- and HFD-fed mice with or without function knockout mice (USP24^WT^ & USP24^C1695A^) was determined using an ultrasound machine (**D**, **a**), and the signal highlight with yellow dotted circle was quantified by Image J, and statistical analysis was conducted by t-test, *p < 0.05 (**D**, **b**). Lipid droplet formation in primary hepatocytes isolated from USP24^WT^ and USP24^C1695A^ mice treated with free fatty acid (500 μM FFA) was examined via oil red O staining assay (**E**, **a**). Subsequent quantification of lipid signaling levels followed three independent experiments, with statistical analysis performed using a t-test, ***p < 0.001 (**E**, **b**)

Previous studies on the role of USP24 in neurodegenerative diseases and cancer progression have been conducted, but its role in MAFLD progression remains unclear. According to previous studies on USP24, the loss of cys1698 (cys1695 in mice) in the catalytic motif of USP24 results in enzyme-death [30]. Therefore, we used CRISPR/Cas9 to construct mice with functional knockout of USP24 (USP24^C1695A^). The added Nar I restriction enzyme site was used for genotyping, and the experimental design and genotyping results are shown in Suppl. Figure 1A and Suppl. Figure 1B. The body weights and sizes of USP24^C1695A^ (USP24KO) mice were lower than those of USP24^WT^ mice, implying that USP24 expression might be involved in lipogenesis (Fig. 1A and Suppl. Figure 1C). There was no significant difference in food intake between USP24^WT^ and USP24^C1695A^ mice (Suppl. Figure 1D). The USP24^+/+^ and USP24^±^ mice survived, but approximately 50% of the USP24^−/−^ (USP24^C1695A/C1695A^) newborn mice died 1 week after birth (Fig. 1B). However, the mice who survived the first week after birth tended to survive, and the physical activity of the USP24^WT^ and USP24^C1695A^ mice was not significantly different (Fig. 1B, right panel). USP24^WT^ and USP24^C1695A^ (USP24^−/−^) mice fed a high-fat diet (HFD) for 19 weeks were used to study the role of USP24 in lipogenesis (Fig. 1C and D). Compared with USP24^WT^ mice, USP24^C1695A^ mice had significantly decreased body weights under normal diet (ND) and high-fat-diet (HFD) conditions (Fig. 1C, e). The blood glucose levels in USP24^C1695A^ mice were lower than those in USP24^WT^ mice under HFD conditions (Suppl. Figure 1E). The lipid droplet formation (Suppl. Figure 2A) and insulin expression (Suppl. Figure 2B) were decreased, and there was no significant change in pancreatic β-cells in USP24^C1695A^ mice (Suppl. Figure 2C), implying that USP24 might also be involved in diabetes (Suppl. Figure 2). After 19 weeks of ND or HFD feeding, USP24^WT^ HFD-fed mice were larger than USP24^C1695A^ HFD-fed mice were, suggesting that the loss of USP24 enzyme activity inhibited obesity (Fig. 1C, a). Furthermore, lipid droplets accumulated in the livers of USP24^WT^ HFD-fed mice but not in those of USP24^C1695A^ HFD-fed mice, indicating that the loss of USP24 may inhibit fatty liver development (Fig. 1C, b, d). Interestingly, visceral adipose tissue (VAT) around the kidneys was found in HFD-USP24^WT^-mice but not in ND- and HFD-USP24^C1695A^-mice (Fig. 1C, c, f), indicating that the loss of USP24 enzyme activity inhibits fat accumulation in vivo. Ultrasound was also used to study the fat accumulation in vivo (Fig. 1D). The data revealed an obvious signal in the livers of HFD-USP24^WT^ male and female mice but not in those of HFD-USP24^C1695A^ mice (Fig. 1D, a, b and Suppl. Figure 1F). The role of USP24 in lipogenesis in hepatocytes was investigated (Fig. 1E and Suppl. Figure 3). First, we used a hepatoma cell line, Huh-7 cells, to study this question (Suppl. Figure 3). USP24-i-101, which targets USP24, did not inhibit lipogenesis in Huh-7 (Suppl. Figure 3A-3C). Furthermore, we directly cultured primary hepatocytes from USP24^WT^ and USP24^C1695A^ mice to study the effect of USP24 expression on hepatocyte lipogenesis (Fig. 1E). Compared with those from USP24^WT^ mice, primary hepatocytes cultured from the livers of USP24^C1695A^ mice showed dramatically decreased FFA-induced lipid droplet formation (Fig. 1E, b). In summary, USP24 expression not only facilitates fatty liver but also facilitates the accumulation of visceral adipose tissue (VAT) around organs and subcutaneous adipose tissue (SAT), which induces several related diseases, such as liver cirrhosis, cardiovascular disease, and diabetes.

### USP24-i-101, which targets USP24, reduces the size of adipose tissues

**Fig. 2:**
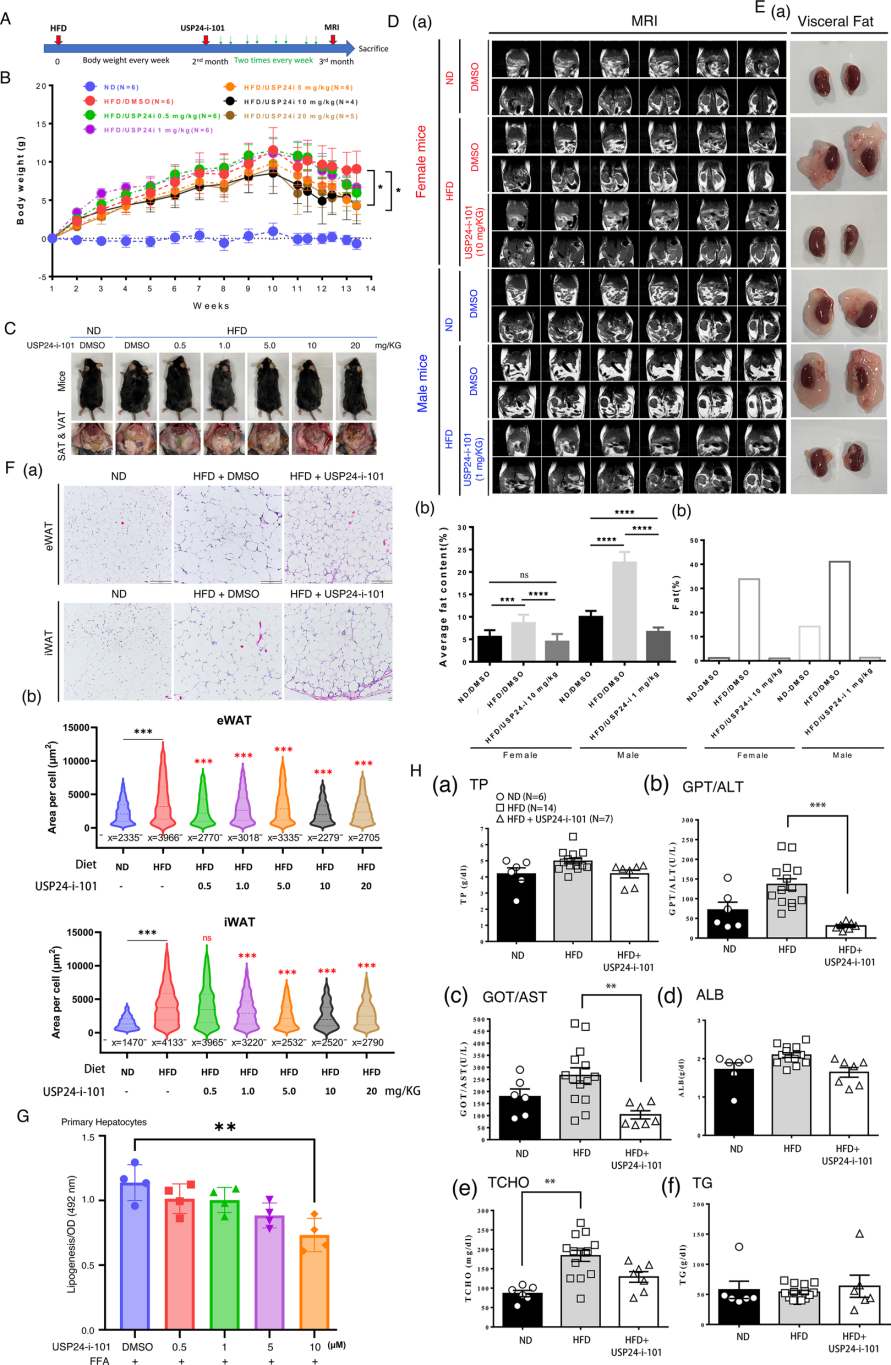
USP24-i-101, which targets USP24, inhibits lipogenesis. USP24^WT^ and USP24^C1695A^ mice were fed a normal diet (ND; n = 6) or a high-fat diet (HFD; n = 33) for 2 months and then treated with 0.5 mg/kg (n = 6), 1.0 mg/kg (n = 6), 5 mg/kg (n = 6), 10 mg/kg (n = 4) or 20 mg/kg (n = 5) USP24-i-101 twice a week for 1 month (**A**). Body weight changes in all mice were measured weekly (**B**). Mouse sizes and their lipid, SAT and VAT, contents, are presented (**C**). Lipid signals in USP24^WT^ and USP24^C1695A^ mice on either an ND or HFD with or without USP24-i-101 treatment were measured using MRI (**D**, **a**). The signal was quantified by Image J, and statistical analysis was performed by t-test, ***p < 0.001, ****p < 0.0005, ns: non-significant (**D**, **b**). Visceral adipose tissue (VAT) around the kidneys of female and male mice with or without USP24-i-101 treatment was shown (**E**, **a**), The area of VAT was quantified by Image J, and statistical analysis was conducted by t-test, ***p < 0.001 (**E**, **b**). iWAT and eWAT in male mice on either an ND or HFD with or without 1 mg/kg USP24-i-101 treatment are depicted (**F**, **a**). Adipocyte sizes were measured using Image J, and statistical analysis was performed by t-test, **p < 0.01, ***p < 0.001 (**F**, **b**). Primary hepatocytes isolated from the mice were treated with various doses of USP24-i-101 and FFA for 24 h, after which lipid droplet formation was studied by oil red O staining. After three independent experiments, lipid droplets contents were quantified, and statistical analysis was performed via a t-test, **p < 0.01 (**G**). Serum collected from the mice was used to study the levels of several lipogenesis-related markers, such as total protein (TP), GPT/ALT, GOT/AST, ALB, total cholesterol (TCHO) and triglyceride (TG) levels, via ELISA, followed by statistical analysis via a t-test, **p < 0.01, ***p < 0.001 (**H**)

We employed a specific USP24 inhibitor, USP24-i-101, to assess the therapeutic effect of USP24-i-101 on inhibiting lipogenesis mediated by USP24 (Fig. 2). Mice were pre-fed a HFD for 2 months and subsequently treated with various doses of USP24-i-101 twice a week for one month (Fig. 2A). The food intake of HFD-fed mice was not different from that of control mice with or without USP24-i-101 treatment (Suppl. Figure 4A). The body weights of the mice were measured every week (Fig. 2B). Compared to DMSO-treated mice, the body weights of USP24-i-101-treated mice were decreased by approximately 3–5 g (Fig. 2B). The body, VAT and SAT sizes of the mice were significantly decreased following USP24-i-101 treatment (Fig. 2D and Suppl. Figure 4B). Subcutaneous and visceral fat contents were measured via MRI (Fig. 2D). The data showed that the MRI signals representing subcutaneous adipose tissue (SAT) fat (Fig. 2D, a, b) and visceral adipose tissue (VAT) were increased in HFD-fed female and HFD-fed male mice, but 10 mg/kg USP24-i-101 in HFD-fed female mice and 1 mg/kg USP24-i-101 in HFD-fed male mice significantly inhibited fat signaling (Fig. 2D). After sacrifice, the fat collected from different locations was compared between HFD-fed mice that had or had not received USP24-i-101 treatment (Fig. 2E, a, b). The data indicated that visceral fat surrounded the kidneys in HFD-fed mice but was nearly abolished under USP24-i-101 treatment, suggesting that targeting USP24 dramatically inhibited obesity. The sizes of adipocytes in epididymal white adipose tissue (eWAT) and inguinal white adipose tissue (iWAT) were also studied (Fig. 2F). The sizes of adipocytes were increased in HFD-fed mice but were reversed after USP24-i-101 treatment (Fig. 2F). We also cultured wild-type primary hepatocytes to treat them with USP24-i-101 and found that USP24 expression significantly inhibited lipid droplet formation (Fig. 2G and Suppl. Figure 4C). In addition, serums collected from mice were used to investigate the biochemical analysis, and results indicated that total protein (TP), albumin (ALB) and triglyceride (TG) levels were not significantly different. GPT/GOT and TCHO expression were markedly inhibited under USP24-i-101 treatment, suggesting that USP24-i-101 is safe at the working dose (Fig. 2H).

### USP24 promotes adipogenesis

**Fig. 3:**
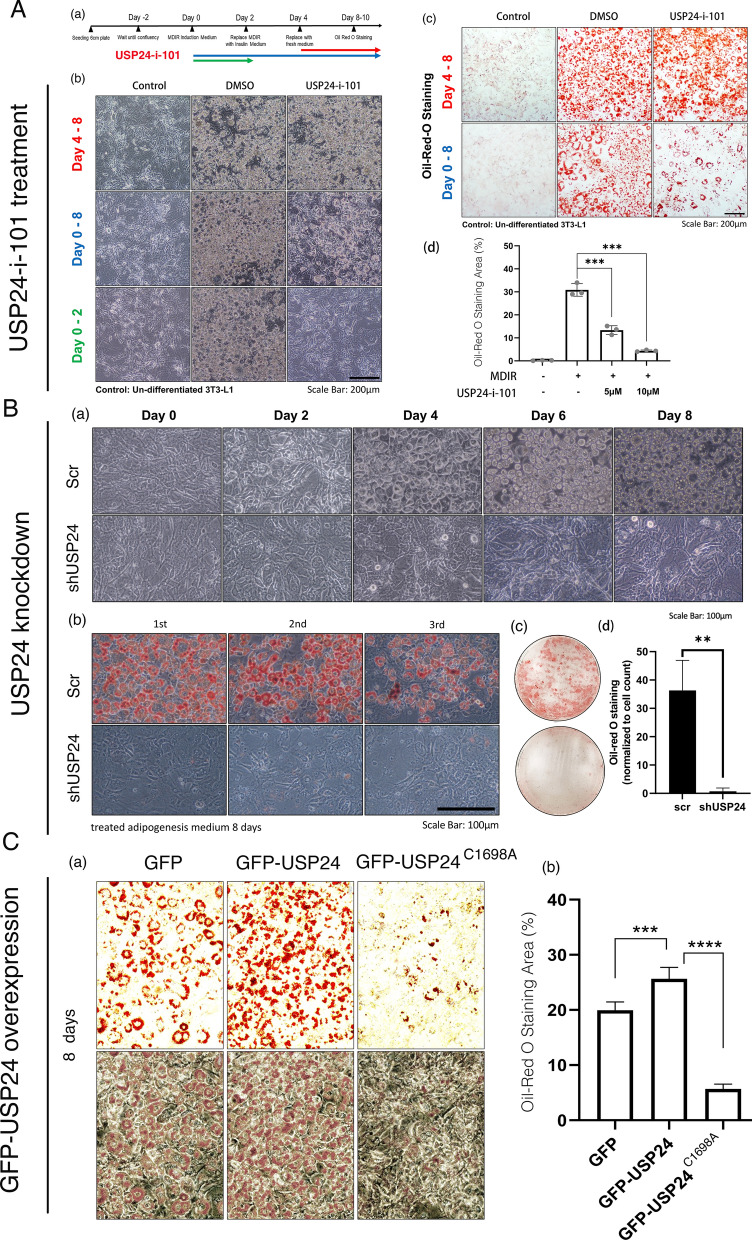
Upregulation of USP24 promotes adipogenesis. The schedule for adipocyte differentiation from 3T3-L1 cells and the cellular morphology with or without USP24-i-101 treatment at different time points, days 0–2, days 0–8 and days 4–8, are shown (**A**, **a**, **b**). Lipid droplet content during adipogenesis with or without 10 μM USP24-i-101 treatment were measured via oil red O staining (**A**, **c**), and the number of lipid droplets (day 0–8) was quantitated using Image J (**A**, **d**). 3T3-L1 cells with or without USP24 expression knockdown by shUSP24 lentivirus were observed (**B**). The morphology of the cells was observed (**B**, **a**), and fat accumulation was determined by oil red O staining (**B**, **b** and **c**). The levels of fat in adipocytes were quantified after three independent experiments (**B**, **d**). GFP, GFP-USP24^WT^, and GFP-USP24.^C1698A^ were overexpressed in 3T3-L1 cells during adipogenesis. Lipid droplet formation was measured by oil red O staining (**C**, **a**). After three independent experiments, the contents of lipid droplets were quantified via Image J (**C**, **b**). Statistical analysis was performed via a t-test, **p < 0.01, ***p < 0.001, ****p < 0.0001

Next, we investigated the mechanisms through which USP24 regulates body weight and lipid accumulation. Previous studies have shown that adipocyte formation is a major factor in increased body weight and lipid accumulation. Here, we studied the effect of USP24 on adipocyte formation using oil red O staining (Fig. 3). 3T3-L1 cells were differentiated into adipocytes with or without USP24-i-101 treatment (0-8th day) (Fig. 1A). The data indicated that lipid droplets were dramatically accumulated in control differentiated cells (treated with DMSO) but could be inhibited after USP24-i-101 treatment, suggesting that USP24 expression positively regulates adipogenesis (Fig. 3A). To study the effect of USP24-i-101 on differentiation stage of adipocytes, USP24-i-101 was added at the early stage (0-2nd day) or late stage (4th–8th day) to study adipocyte differentiation. The data indicated that the addition of USP24-i-101 at an early stage inhibited adipocyte formation, while the addition of USP24-i-101 at a late stage did not, implying that USP24 expression is involved in adipocyte differentiation at an early stage (Fig. 3A). USP24 expression was also silenced during 3T3-L1 differentiation to address the role of USP24 in lipogenesis (Fig. 3B). According to cell morphology (Fig. 3B, a) and oil red O staining results (Fig. 3B, b, c), lipid droplet accumulation was nearly abolished in USP24-knockdown cells, indicating that USP24 expression is critical for lipogenesis in adipocytes. Finally, GFP, GFP-USP24 and GFP-USP24^C1695A^ were overexpressed in 3T3-L1 cells to study adipogenesis (Fig. 3C). The data indicated that GFP-USP24 overexpression can significantly increase lipid droplet formation but can be inhibited by GFP-USP24^C1695A^ overexpression, suggesting that the enzyme activity of USP24 is required for adipogenesis (Fig. 3C).

### USP24 increases p-CREB, CEBPβ and PPARγ expression to promote adipogenesis

**Fig. 4:**
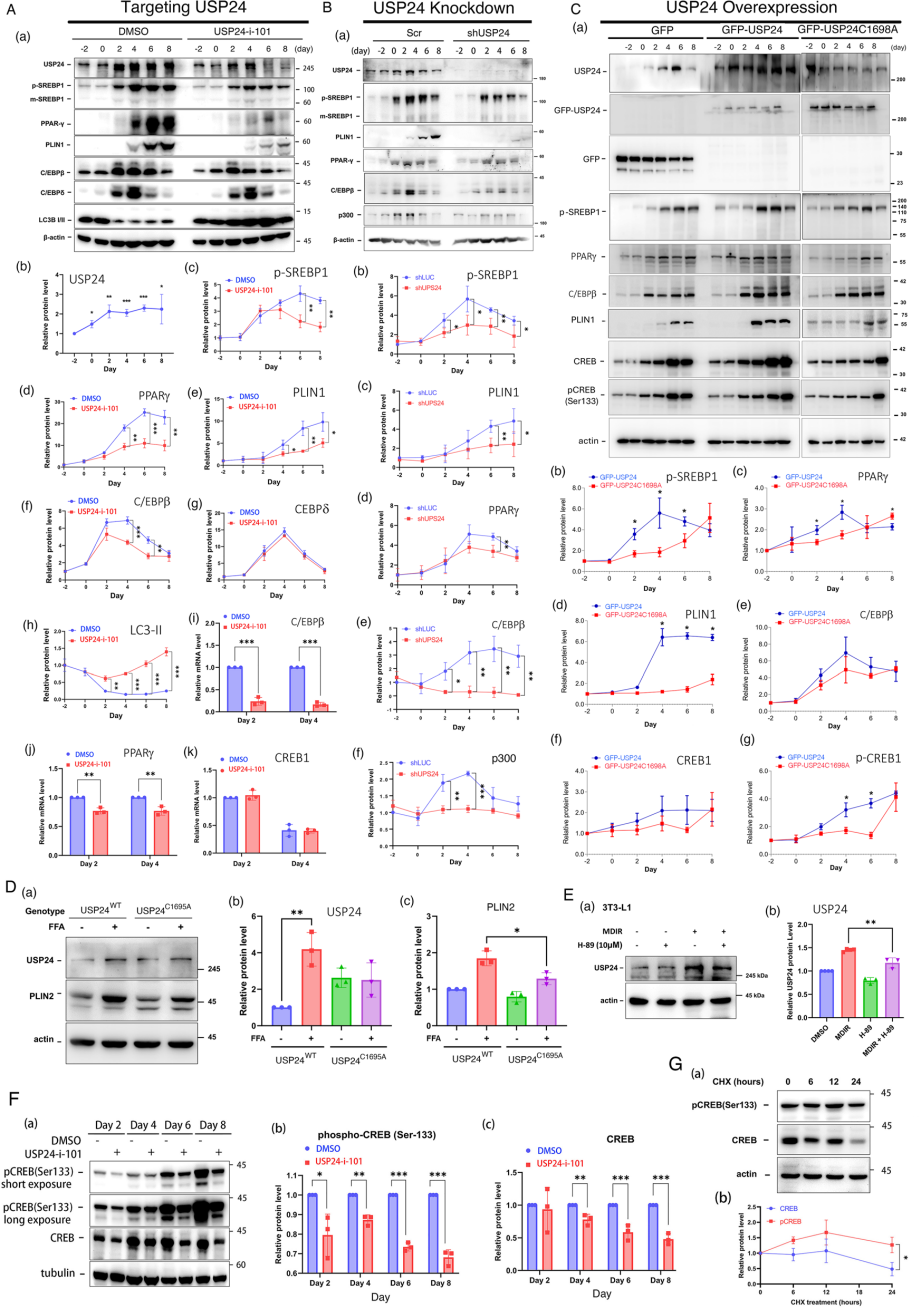
The USP24/PKA/p-CREB/CEBPβ/PPARγ axis regulates adipocyte differentiation. Lipogenesis-related proteins expressed in 3T3-L1 cells treated with 10 μM USP24-i-101 (**A**) were knocked down with shRNA-USP24 (**B**) or overexpressed with GFP/GFP-USP24/GFP-USP24^C1698A^ (**C**) during adipogenesis, and then samples were harvested on the -2nd, 0, 2nd, 4th, 6th and 8th days. The expression of lipogenesis-related proteins was measured via IB with antibodies agonist the indicated proteins (**A**–**C**). Primary hepatocytes isolated from mice with or without USP24 knockout, USP24^WT^ or USP24.^C1695A^, were treated with 500 μM FFA, and then, the cells were lysed to study the expression of USP24 and PLIN2 via IB (**D**). 3T3-L1 cells were treated with MDIR and the PKA inhibitor 10 μM H89 for 24 h, and then the samples were collected in sample buffer. The expression of USP24 was measured via IB with anti-USP24 antibodies (**E**). 3T3-L1 cells with or without 10 μM USP24-i-101 treatment were lysed to study the expression of CREB and pCREB via IB (**F**, **a**). After three independent experiments, the expression of pCREB (**F**, **b**) and CREB (**F**, **c**) were quantified. 3T3-L1 cells were treated with cycloheximide (CHX), and then, cell lysates were collected at the indicated times to study the expression of pCREB/CREB (**G**, **a**). After three independent experiments, the expression of pCREB/CREB (**D**, **b**) and PKA-Cα (**G**, **b**) was quantified. After three independent experiments, various protein expressions were quantified. The statistical analysis was performed via a t-test, *p < 0.05, **p < 0.01, ***p < 0.001

To elucidate how USP24 regulates adipogenesis, 3T3-L1 cells were treated with USP24-i-101, which targets USP24, and the knockdown or overexpression of USP24 was investigated in differentiated 3T3-L1 cells (Fig. 4). The expression of USP24 increased during adipocyte differentiation, suggesting that USP24 expression might be related to adipocyte differentiation in the early period (Fig. 4A, a, b). The expression levels of several lipogenesis-related proteins, including SREBP1c, CREB, p-CREB, C/EBPβ,C/EBPδ, PPARγ, PLIN1, and LC3B, were determined in USP24-i-101-treated adipocytes (Fig. 4A, a, d–i). Data showed that the expression of all proteins except C/EBPδ was significantly inhibited by USP24-i-101 treatment (Fig. 4A, a, d–i). As LC3B expression was also increased here, USP24-i-101 was used to study its effect on autophagy in hepatocytes (Suppl. Figure 5). The data indicated that USP24-i-101 treatment increased LC3B expression in hepatocytes and adipocytes, suggesting that USP24-i-101-mediated autophagy might be involved in adipogenesis (Suppl. Figure 5A-5E). The expression of several lipogenesis-related proteins, SREBP1c, C/EBPβ, PPARγ, PLIN1 and p300, was determined in USP24-silenced adipocytes (Fig. 4B). Data indicated that the expression of all related proteins was significantly decreased in USP24-knockdown cells, implying that the increase in USP24 expression during 3T3-L1 differentiation is involved in lipogenic gene expression (Fig. 4B). GFP, GFP-USP24, or GFP-USP24^C1695A^ was overexpressed in 3T3-L1 cells to study the expression of these adipogenesis-related proteins (Fig. 4C). Data indicated that the expression of all adipogenesis-related proteins, including p-SREBP1, PPARγ, C/EBPβ, PLIN1, CREB, and p-CREB, was increased in GFP-USP24-overexpressing cells but not in the GFP-USP24^C1695A^-overexpressing cells, indicating that the enzyme activity of USP24 is necessary for adipogenesis (Fig. 4C). Primary hepatocytes isolated from USP24 knockout mice were treated with free fatty acid (FFA), and FFA treatment significantly increased the expression of USP24 and PLIN2 in USP24^WT^ mice but only slightly increased the expression of PLIN2 in USP24^C1695A^ mice, suggesting that the enzyme activity of USP24 is important for FFA-mediated induction of lipogenesis in the liver (Fig. 4D). Inhibiting PKA activity with the PKA inhibitor H89 decreased USP24 expression (Fig. 4E), indicating that USP24 and PKA positively regulate each other. How does USP24 promote adipogenesis directly? The early-stage phosphorylation of CREB is critical for adipogenesis [31]. First, CREB protein phosphorylation during 3T3-L1 differentiation with or without USP24-i-101 treatment was measured (Fig. 4F). Data indicated that both the protein phosphorylation and total CREB but not mRNA expression level of CREB were decreased by USP24-i-101 treatment, implying that phosphorylation of CREB may be involved in its protein stability (Fig. 4F, Suppl. Figure 5G). Studying protein stability of p-CREB and CREB under cycloheximide (CHX) treatment found that phosphorylation of CREB can increase its protein (Fig. 4G), indicating that USP24 expression may increase the phosphorylation of CREB, thereby increasing its protein stability. These findings indicate that USP24 expression promotes adipogenesis through positive regulation of lipogenesis-related gene expression.

### USP24 expression stabilizes PKA-Cα and p300 during adipogenesis

**Fig. 5:**
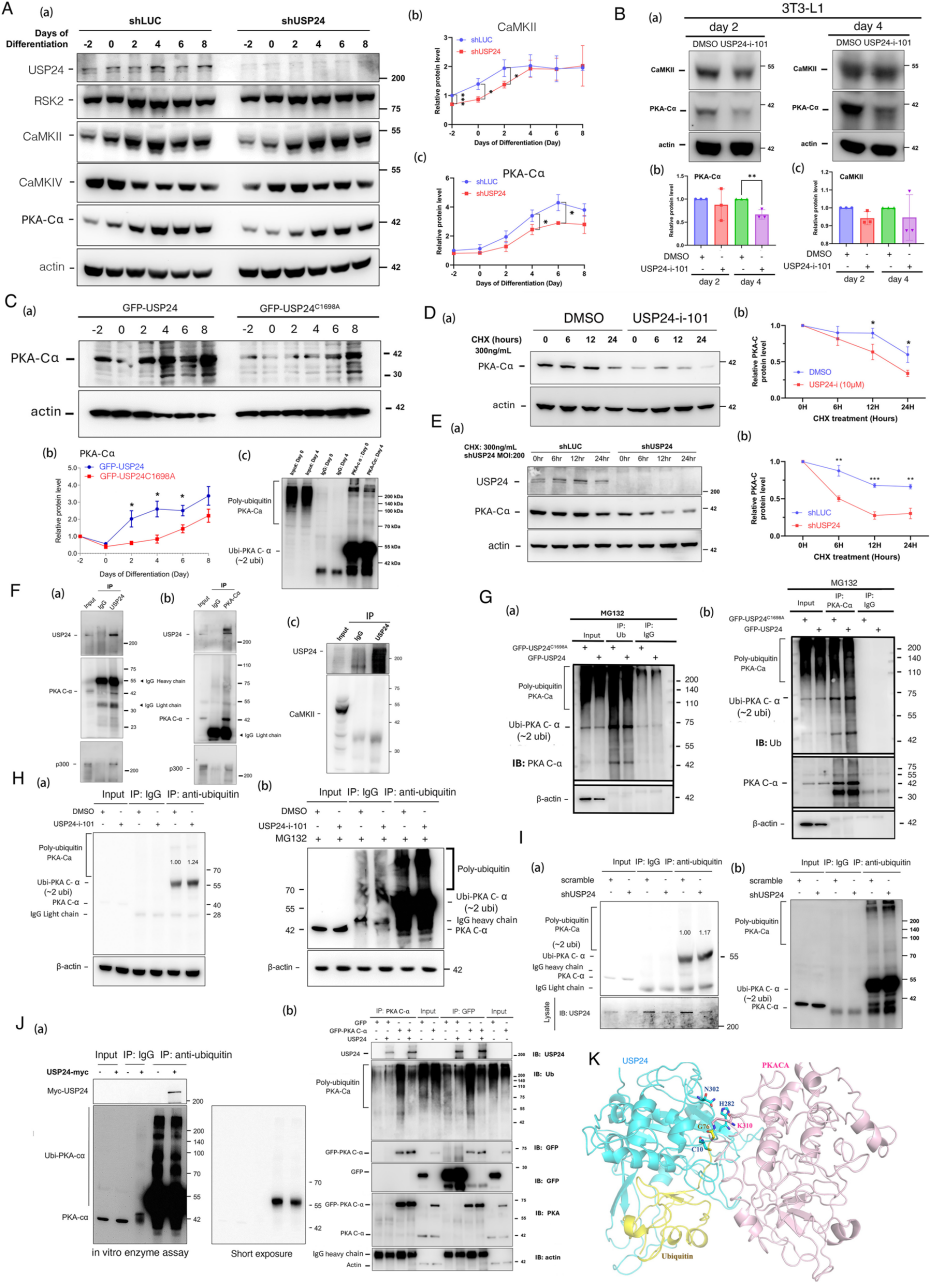
USP24 stabilizes PKA-Cα and p300 during adipogenesis. The expression of USP24 in 3T3-L1 cells was knocked down using shRNA-USP24 for three days, after which the cells were differentiated into adipocytes. Samples were collected at different time points to study the expression of the indicated proteins via IB with antibodies against the indicated proteins (**A**, **a**). After three independent experiments, the expression of CaMKII (**A**, **b**) and PKA-Cα (**A**, **c**) was quantified. 3T3-L1 cells were treated with or without 10 μM USP24-i-101, and samples were collected at day 2 and day 4 (**B**, **a**) to measure the expression of PKA-Cα and CaMKII via IB. After three independent experiments, the expression of PKA-Cα (**B**, **b**) and CaMKII (**B**, **c**) was quantified. GFP-USP24 and GFP-USP24^C1698A^ were overexpressed in 3T3-L1 cells, which were then induced for adipocyte differentiation via MDIR. Samples were collected at different time points to measure the expression of PKA-Cα via IB with anti-PKA-Cα antibodies (**C**). 3T3-L1 cells were treated with cycloheximide (CHX) with or without 10 μM USP24-i-101 treatment (**D**) and USP24 knockdown (**E**), and then cell lysates were collected at the indicated times to study the expression of PKA-Cα (**D** and **E**). After three independent experiments, the expression of PKA-Cα (**D** and **E**, **b**) was quantified. 3T3-L1 cells were differentiated, and samples were collected on the 2nd day for IP with anti-USP24 and IgG (**F**, **a**, **c**) or anti-PKA-Cα and IgG (**F**, **b**), and then IB with antibodies against indicated proteins. GFP, GFP-USP24^WT^ and GFP-USP24^C1698A^ were transfected into 3T3-L1 cells for differentiation, and samples were for IP with anti-GFP antibodies or anti-USP24 antibodies and IB with antibodies against USP24, PKA-Cα, ubiquitin and actin (**G**). 3T3-L1 cells with or without USP24-i-101 (**H**), or USP24 knockdown (**I**) were differentiated with MG132 treatment, and samples were collected on the 2^nd^ day for IP with anti-ubiquitin and IgG and IB with antibodies against PKA-Cα. GFP and GFP-PKA-Cα were overexpressed in 3T3-L1 cells for 2 days. Polyubiquitinated PKA-Cα was pulldown by IP with anti-ubiquitin antibodies (**J**, **a**), anti-PKA-Cα and anti-GFP antibodies (**J**, **b**), followed by the addition of myc-USP24 (1 μg recombinant protein) for 1 h. The levels of various proteins were measured by IB with indicated antibodies (**J**). The generation of the USP24-ubiquitin-PKA-Cα complex may indicate its function. An irreversible bond is formed between ubiquitin G76 and PKA-Cα K310. The catalytic site of USP24 envelops the ubiquitin-PKA-Cα bond and can be near the USP24 catalytic residues C10, H282, and N302. The modelled complex suggests potential functions for further cellular processing of the complex (**K**)

Since the data in Fig. 4 suggested that USP24 expression might be involved in the early period of adipogenesis, we studied the mechanism by which USP24 regulates signaling pathway during adipogenesis in detail (Fig. 5). Several kinases, including RSK2, CaMKII, CaMKIV and PKA, have been reported to phosphorylate CREB [31]. Our findings showed that treatment with USP24-i-101 or knockdown of USP24 expression decreased the expression of CaMKII and the PKA catalytic subunit α (PKA-Cα) (Fig. 5A and B). In contrast, overexpression of GFP-USP24^WT^ in 3T3-L1 cells increased PKA-Cα expression compared with USP24^C1698A^ overexpression (Fig. 5C, a, b). Additionally, the ubiquitination signal of PKA-Cα was reduced on day 4 compared to day 0 (Fig. 5C, c), suggesting that the enzyme activity of USP24 is required for adipogenesis. Treatment with USP24-i-101 and knockdown of USP24 expression also decreased the protein stability of PKA-Cα (Fig. 5D and E), implying that PKA-Cα may be as a substrate of USP24. Since p300 can bind to CREB to increase its activity [22], our previous studies revealed that p300 is the substrate of USP24 [31]. We also found that the knockdown of USP24 expression decreased the protein expression of p300 but did not significantly change its mRNA expression (Fig. 4B and Suppl. Figure 5F). PKA-Cα and p300 can interact with USP24 but not CaMKII (Fig. 5F). Overexpression of GFP-USP24 in 3T3-L1 cells increased PKA-Cα expression and decreased the ubiquitination signal of PKA-Cα through a proteasome-dependent manner, whereas this effect was not observed when GFP-USP24^C1695A^ was overexpressed (Fig. 5G). Conversely, treatment with USP24-i-101 or knockdown of USP24 expression increased the ubiquitination signal of USP24 in a proteasome-dependent manner under similar PKA-Cα level condition (Fig. 5H, I). In in vitro enzyme assays revealed that USP24 can eliminate the ubiquitination of PKA-Cα, possibly including the polyubiquitination and monoubiquitination of PKA-Cα (Fig. 5J). Previous studies have indicated that two residues, K286 and K310, in PKA-Cα can be ubiquitinated by its E3-ligase [32]. A model of the USP24-ubiquitin-PKA-Cα complex was generated to gain better insight into its structure (Fig. 5K). The USP24 catalytic site is positioned close to the ubiquitin-PKA-Cα protein. Molecular Dynamic dynamics simulations revealed that USP24 residues are positioned near the USP24-ubiquitin-PKA-Cα complex, including USP24 residues C10, H282, and N302, which are positioned near the PKA-Cα G76 and ubiquitin K310 bonds. The position of USP24 facilitates tagging for additional cellular processing of the complex. Finally, a cAMP analog, rp-cAMPs, was employed to compete with cAMP, thereby inhibiting the activity of PKA-Cα, to address the synthesis of fatty acid in adipocytes (Suppl. Figure 5H). The synthesis of fatty acid was partially inhibited by rp-cAMPs, but can be totally inhibited by USP24-i-101, indicating that other USP24-regulated factors, such as CaMKII, also contribute to the effect of USP24 in adipogenesis (Suppl. Figure 5H). In summary, USP24 not only interacts with PKA-Cα to positively regulate each other, increasing CREB1 phosphorylation but also stabilizes p300 and increases the transcriptional activity of p-CREB during adipogenesis.

### USP24 positively regulates lipogenesis, inflammation, and fibrosis gene expression in HFD-fed mice

**Fig. 6:**
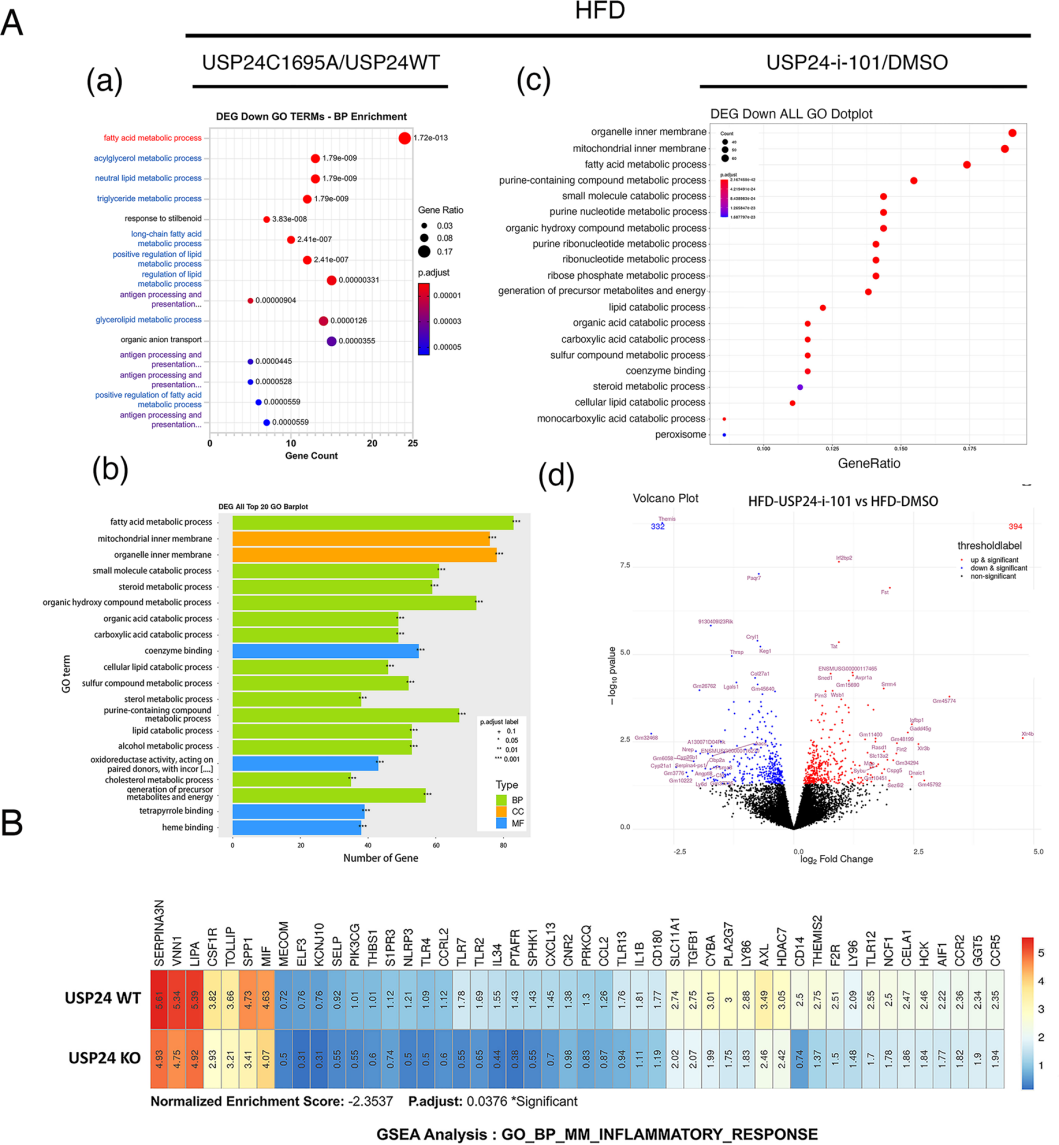

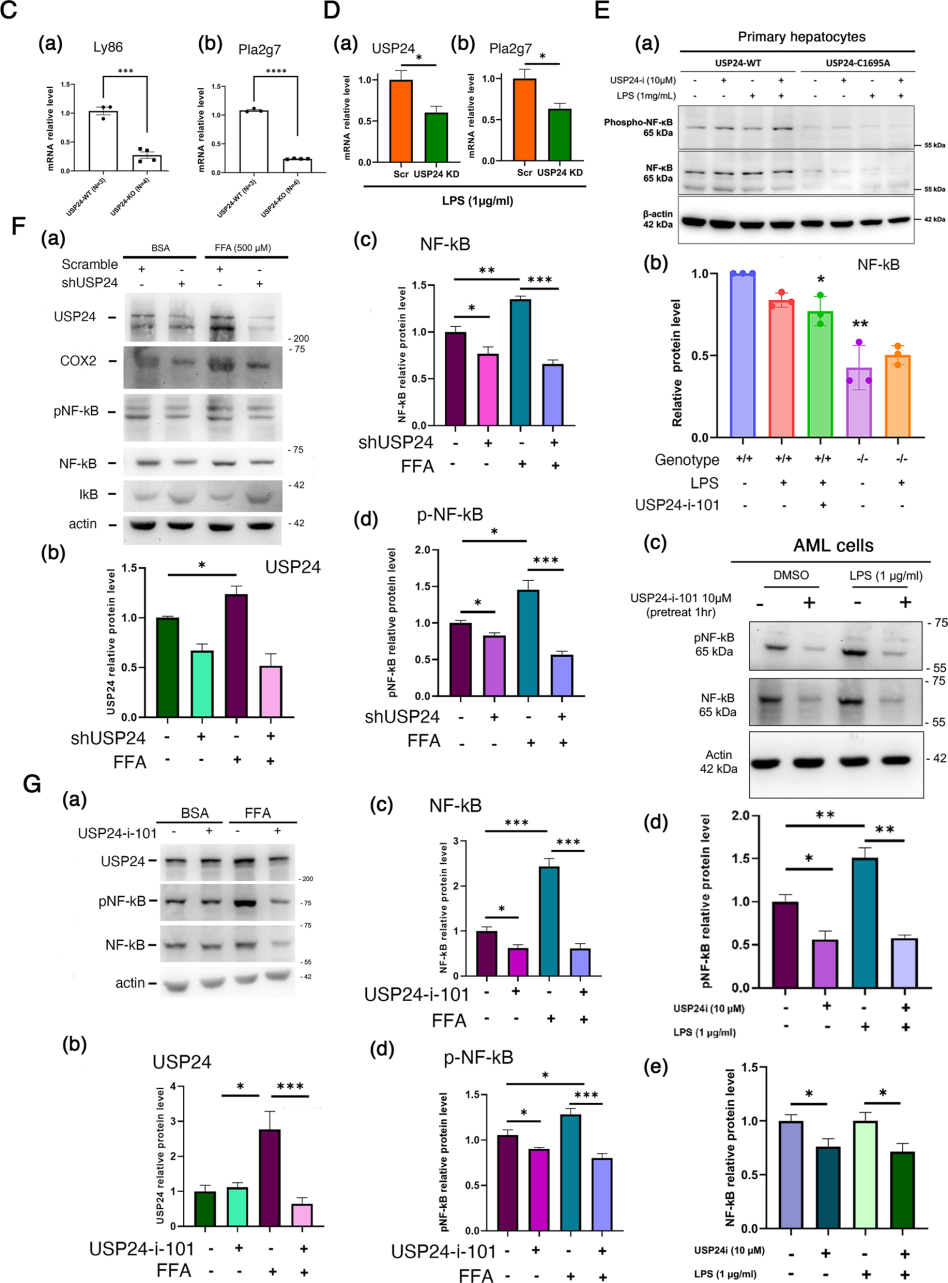
Numerous genes related to lipogenesis are regulated by USP24. Total RNA samples were isolated from the liver tissues of USP24^WT^, USP24^C1695A^ or USP24-i-101-treated mice fed a ND or HFD for RNA-Seq and then analyzed via the bioinformatics tools DEG Down GO TERMs – BP Enrichment (**A**, **a**; USP24^C1695A^/USP24^WT^), DEG All Top 20 GO Barplot (**A**, **b**; USP24^C1695A^/USP24^WT^), DEG Down ALL GO Dotplot (**A**, **c**; 1 mg/KG USP24-i-101, i.p., two times per week) and Volcano Plot (**A**, **d**; 1 mg/KG USP24-i-101, i.p., two times per week). Inflammation-related genes whose expression was regulated by USP24^C1695A^ were studied via RNA-seq and analyzed with a heatmap (**B**, USP24^C1695A^/USP24^WT^). Total RNA was extracted from mice with or without USP24 expression knockout to study the mRNA expression of Ly86 (**C**, **a**) and pLa2g7 (**C**, **b**) via qPCR. AML12 cells with or without USP24 knockdown were treated with LPS for 2 h, and the mRNA expression of USP24 (**D**, **a**) and Pla2g7 (**D**, **b**) were measured via qPCR. Primary hepatocytes isolated from mice with or without USP24 knockout (**E**, **a**, **b**) or hepatocyte AML cells (**E**, **c**, **d**, **e**) were treated with LPS or USP24-i-101. The expression of USP24, p-NF-κB and NF-κB were measured via IB (**E**). AML12 cells with or without USP24 knockdown (**F**) or 10 μM USP24-i-101 treatment (**G**) were treated with 500 μM free fatty acid (FFA) or BSA for 24 h, after which the samples were harvested to measure the expression of USP24,COX2, IκB, p-NF-κB and NF-κB via IB. After three independent experiments, the quantification and statistical analysis were performed via t-test: *p < 0.05, **p < 0.01, ***p < 0.001

To study the effect of USP24 on gene expression systemically, liver organs collected from normal diet (ND)-fed and high-fat diet (HFD)-fed mice with or without USP24 functional knockout (USP24^C1695A^) were used to investigate the gene expression profiles via RNA-seq (Fig. 6, Suppl. Figure 6-Suppl. Figure 11 and Suppl. Table 1-Table 2). First, the concentration and quality of all the RNA samples were evaluated (Suppl. Figure 7A). All the control results, including the error rate distribution, base content distribution, Trimmomatic results, distribution of gene expression levels, Pearson correlation coefficient, Trimmomatic Trim results summary and principal component analysis (PCA), supported the quality of these RNA-seq results (Supp. Figure 6). After analysis with bioinformatics tools, 236 genes in the livers of USP24^WT^ mice but only 52 genes in the livers of USP24^C1695A^ mice were found to show upregulated expression in HFD-fed mice compared with ND-fed mice (Suppl. Figure 7B, b, c), implying that USP24 regulates lipogenesis-related gene expression. However, 103 genes in female USP24-knockout HFD-fed mice and 113 genes in male USP24^C1695A^ HFD-fed mice showed significantly downregulated expression compared with those in USP24^WT^ HFD-fed mice (Suppl. Figure 7B–Suppl. Figure 7C). Most expression of the genes related to fatty liver and diabetes, such as LPIN1, LCN2 and CTSB, in USP24^C1695A^ HFD-fed mice were downregulated, suggesting that USP24 is involved in lipogenesis (Suppl. Figure 8). Acetyl-CoA carboxylase (ACACB), which catalyzes the carboxylation of acetyl-CoA to malonyl-CoA, the rate-limiting step in fatty acid synthesis, and ACACB expression was also significantly downregulated in USP24^C1695A^ HFD-fed mice (Suppl. Figure 8). The abundance of stearoyl-CoA 9-desaturase 1 (SCD1), an iron-containing enzyme that catalyzes a rate-limiting step in the synthesis of unsaturated fatty acids, was dramatically decreased in USP24^C1695A^ HFD-fed mice (Suppl. Figure 8). Another gene, secreted phosphoprotein 1 (SPP1), which is overexpressed in many cancer types and correlated with poor prognosis, was also significantly downregulated expression in USP24^C1695A^ HFD-fed mice (Suppl. Figure 8) ^30,31^. By using GO enrichment (Suppl. Figure 9A) and DEG analysis, we also found that most of the genes related to lipogenesis, such as those related to fatty acid metabolism, acyl-CoA metabolism, steroid metabolism, and lipid catabolism, had downregulated expression in USP24-knockout HFD-fed mice (Fig. 6A, b and Suppl. Figure 9A). In addition, biological pathways (DEG Down GO TERMs-BP Enrichment; Fig. 6A, a and Suppl. Figure 10A) and disease pathways (DEG Down DisGeNET Enrichment Pathway; Suppl. Figure 9B and Suppl. Figure 10B) were regulated by USP24. Other genes related to diseases such as lupus vulgaris, amyloid neuropathies, aortic aneurysm and chronic kidney diseases also showed downregulated expression in USP24^C1695A^ mice (Suppl. Figure 10B). Many lipogenesis-related diseases, such as fatty liver, hyperinsulinism, diabetes, and dyslipidemia, were significantly inhibited in USP24^C1695A^ -overexpressed or USP24-i-101-treated HFD-fed mice, implying that targeting USP24 might prevent these diseases (p < 0.00005; Fig. 6A, a and c). In the DEG Down GO TERM-CC enrichment analysis, we found that the loss of USP24 expression decreased the activity of several lipid trafficking pathways, such as the lysosome, lytic vacuole and lipoprotein particle pathways (Suppl. Figure 10C). In the DEG downregulated KEGG enrichment analysis, pathways related to lysosomal activity, biosynthesis of unsaturated fatty acids, the PPAR signaling pathway and alcoholic liver disease had downregulated expression in USP24-knockout HFD-fed mice (Suppl. Figure 11A). In the DEG Down DO Enrichment Pathway, pathways related to arteriosclerotic cardiovascular disease obesity were significantly inhibited by knocking out USP24 (Suppl. Figure 11B). Numerous genes related to β-oxidation (Suppl. Figure 12A, a), adipogenesis (Suppl. Figure 12B, b), glycolysis and gluconeogenesis (Suppl. Figure 12C, a, b) had downregulated expression in USP24^C1695A^ mice (Suppl. Figure 12).

We examined the global gene expression profiles not only in HFD-USP24^C1695A^ mice but also in USP24-i-101-treated mice (Fig. 6A, c and d, Suppl. Figure 9C-9E). All control results, including H&E staining results of the RNA-seq samples (Suppl. Figure 13), the error rate distribution (Suppl. Figure 14A), the base content distribution (Suppl. Figure 14B), Trimmomatic results (Suppl. Figure 14C), the distribution of gene expression levels (Suppl. Figure 14D), Pearson correlation coefficient (Suppl. Figure 14E) and Trimmomatic Trim results summary (Suppl. Figure 14F), supported the quality of these RNA-seq results. We used a heatmap (Suppl. Figure 9C) and volcano plot (Fig. 6A, d) to address the effect of USP24-i-101, which targets USP24, on the systemic gene expression profile of HFD-fed mice (n = 3). A total of 332 genes showed downregulated expression, and 394 genes showed upregulated expression following the 5 μM USP24-i-101 treatment (Suppl. Figure 9C and Fig. 6A, d). Several lipocalin (LCN)-like genes, such as Mup1, Mup3, Mup12, Mup15, Mup16, Mup7, Mup11, Mup17 and Mup14, had upregulated expression induced by USP24-i-101 treatment, which is consistent with the inhibition of obesity and diabetes [33, 34]. Several Cyp genes, such as Cyp4a14, Cyp3a11 and Cyp4a10, had downregulated expression in mice treated with USP24-i-101, which targets USP24 (Fig. 6A, d). Several lipogenesis-related pathways, such as the fatty acid metabolic process, lipid catabolic process, alcohol metabolic process, cholesterol metabolic process and steroid metabolic process, were downregulated in DEG ALL GO Dotplot (Suppl. Figure 9D) and DEG Down ALL GO Dotplot (Fig. 6A, c). However, several pathways related to the collagen-containing extracellular matrix, negative regulation of proteolysis and the humoral immune response had decreased activity according to the DEG Up ALL GO Dotplot (Suppl. Figure 9E). Finally, not only fatty acid metabolism-related genes (Suppl. Table 1) but also inflammation-related genes (Fig. 6B and Suppl. Table 2) and fibrosis-related genes (Suppl. Table 3) had downregulated expression in USP24^C1695^^A^ mice, implying that targeting USP24 might potentially inhibit metabolic associated fatty liver disease (MAFLD). The expression of two inflammatory-related genes, Ly86 and Pla2g7, was validated in mice with or without USP24 knockout by qPCR (Fig. 6C). Indeed, the loss of USP24 expression decreased the mRNA expression of Ly86 and Pla2g7 (Fig. 6C). Hepatocyte AML12 cells with or without USP24 knockdown were treated with LPS to measure the expression of Pla2g7 (Fig. 6D). The data indicated that USP24 knockdown decreased the mRNA expression of LPS-induced Pla2g7 in AML12 cells (Fig. 6D). WT and USP24-knockout hepatocytes isolated from mice (Fig. 6E, a, b) or hepatocyte AML cells (Fig. 6E, c, d, e) were used to study the effect of USP24 expression on inflammation with or without LPS or USP24-i-101 treatment. The levels of NF-κB and p-NF-κB were significantly decreased in USP24^C1695A^ and USP24-i-101-treated USP24^WT^ primary hepatocytes (Fig. 6E, a, b) and AML cells (Fig. 6E, c, d, e), suggesting that the loss of USP24 expression in mice decreases the activity of the NF-κB signaling pathway, thereby inhibiting inflammation (Fig. 6E). Finally, the effect of USP24 expression on inflammation in FFA-treated AML cells with or without USP24 knockdown (Fig. 6F) or USP24-i-101 treatment (Fig. 6G) was also investigated. The expressions of NF-κB and p-NF-κB (p-p65) were inhibited in USP24-knockdown (Fig. 6F) or USP24-i-101-treated (Fig. 6G) FFA-treated AML cells. Together, these findings suggest that USP24 promotes inflammation and fibrosis during MASH progression. In summary, not only in USP24 knockout mice but also in USP24-i-101-treated mice, most lipogenesis-, inflammation- and fibrosis-related genes had downregulated expression, indicating that USP24-i-101, which targets USP24, can inhibit MASH.

### Inhibition of USP24 expression suppresses inflammation- and fibrosis-related gene expression

**Fig. 7:**
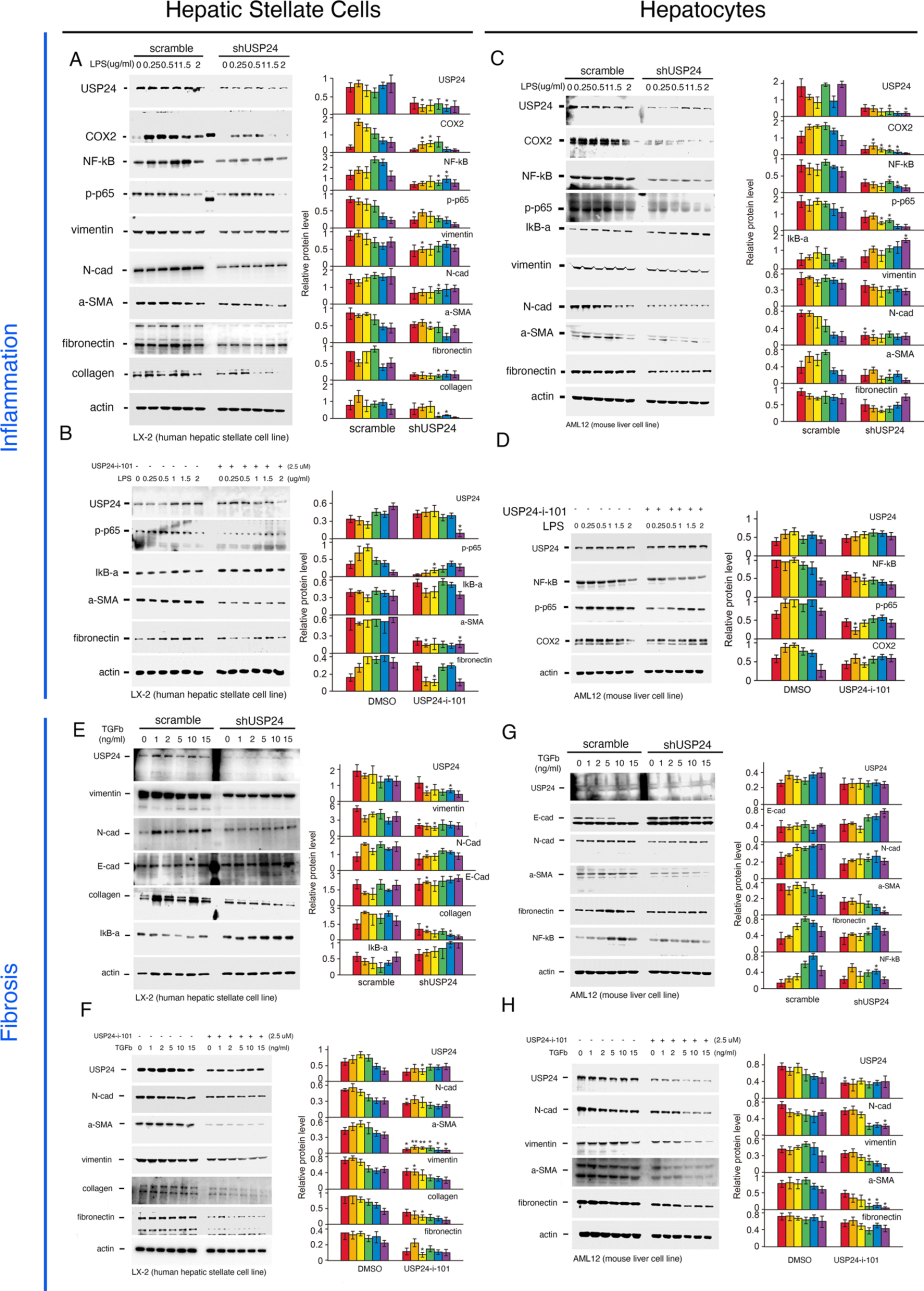
USP24 is involved in the expression of MASH-related proteins. USP24 expression was knocked down (**A**) or treated with 2.5 μM USP24-i-101 (**E**) in LX-2 cells (**B**) with or without LPS treatment, and samples were harvested to study the expression of the indicated proteins via IB. USP24 was knocked down (**C**) or treated with 2.5 μM USP24-i-101 (**D**) in AML12 cells with or without LPS treatment, and samples were harvested to study the expression of the indicated proteins via IB. USP24 was knocked down (**E**) or treated with 2.5 μM USP24-i-101 (**F**) in AML12 cells and LX-2 cells (**G**, **H**) with or without TGFβ treatment, and samples were harvested to study the expression of the indicated proteins via IB. After three independent experiments, the quantification and statistical analysis were performed via t-test: *p < 0.05, **p < 0.01

To date, we have elucidated the role and mechanism of USP24 in adipocytes. Does USP24 expression participate in MASH progression in the liver? To further investigate the role of USP24 expression in the liver, several experiments related to MASH were conducted here (Fig. 7). All the pathways associated with fibrosis showed decreased activity in USP24 knockout mice (Suppl. Figure 15A). The number of cells with macrovascular steatosis in the livers of the mice was determined (Suppl. Figure 14 and Suppl. Figure 15B). The number of cells with macrovascular steatosis was higher in HFD-fed mice than in ND-fed mice, and the knockout of USP24^WT^ (USP24^C1695A^) decreased the number of these cells (Suppl. Figure 15B and Suppl. Figure 16). Additionally, the mRNA expression of fibrosis-related genes α-SMA, Cola1 and TGF-β1 was significantly inhibited by USP24-i-101 treatment in primary hepatocytes (Suppl. Figure 15C). USP24 expression was knocked down or treated with USP24-i-101 in LX-2 cells (Fig. 7A and B) and AML12 cells (Fig. 7C and D) with or without LPS treatment to study the expression of inflammation- and fibrosis-related proteins. The expression of COX2, NF-κB, p-p65, vimentin, NLRP3, N-cadherin (N-cad), α-SMA, fibronectin and collagen were significantly decreased, and IκB expression was increased in LX-2 cells (Fig. 7A and B) and AML12 cells (Fig. 7C and D), suggesting that USP24 expression positively regulates inflammation in the liver. Since TGF-β can induce fibrosis in the liver, USP24 plays a role in TGF-β-treated LX-2 cells (Fig. 7E and F) and AML12 cells (Fig. 7G and H). The expression of the fibrosis-related proteins vimentin, N-cadherin, α-SMA, fibronectin and collagen were inhibited in USP24-knockdown LX-2 cells (Fig. 7E) and AML cells (Fig. 7G) and in USP24-i-101-treated LX-2 cells (Fig. 7F) and AML cells (Fig. 7H), while the expression of E-cadherin (E-cad) was increased, indicating that USP24 promotes fibrosis in the liver.

**Fig. 8:**
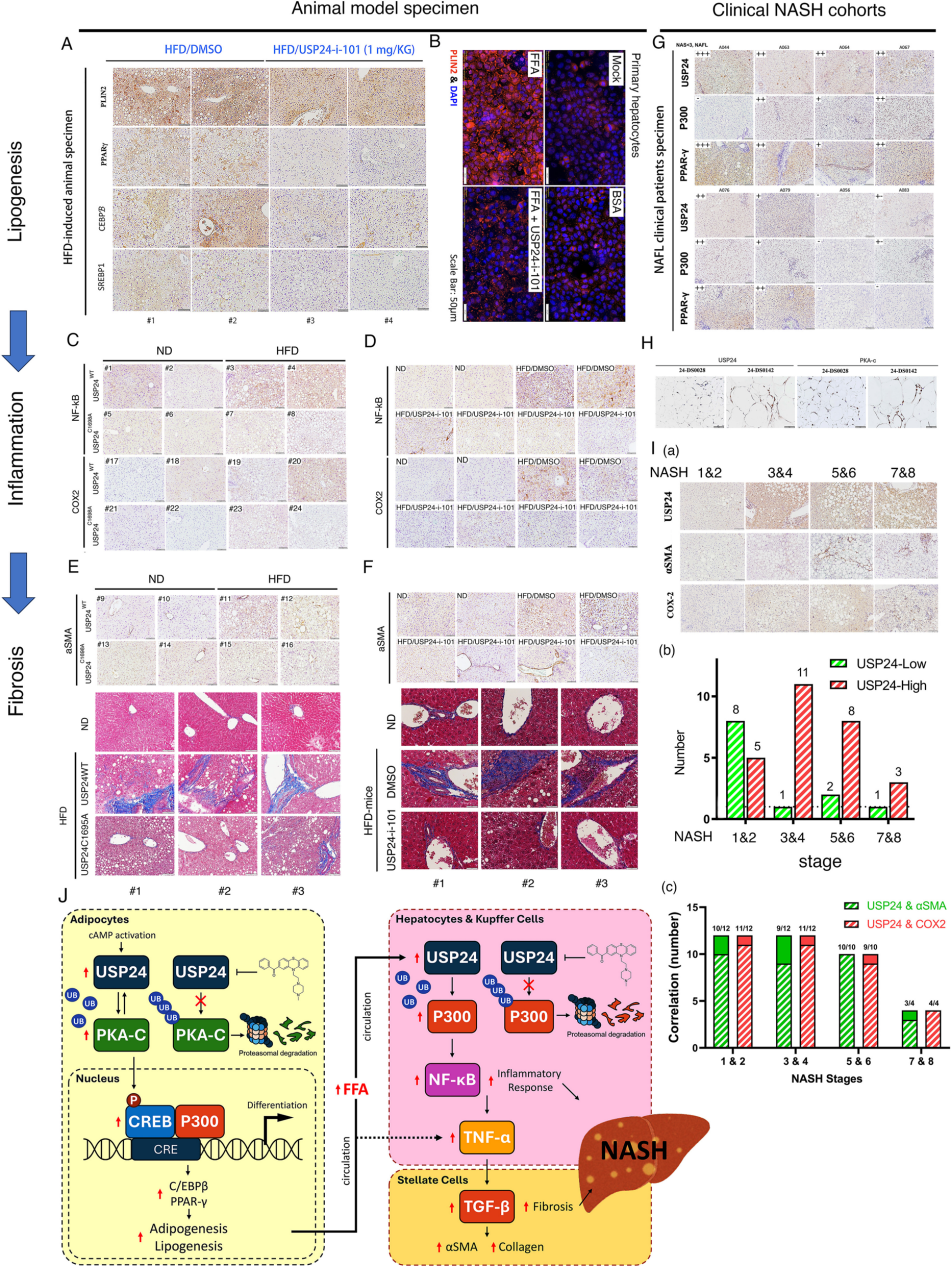
Positive correlation between USP24 expression and that of MAFLD-related markers in animal samples and clinical specimens. The protein expressions of the indicated proteins in the livers of HFD-fed mice with or without USP24-i-101 treatment were measured by IHC (**A**). Primary hepatocytes were isolated from mice treated with free fatty acid (FFA) and USP24-i-101 for 24 h showed lipid level measurements via immunofluorescence with anti-PLIN2 antibodies (**B**). The expression of the inflammation- and fibrosis-related proteins, COX-2, and NF-κB in HFD-fed USP24^WT^ and USP24.^C1695A^ mice (**C**), and HFD-fed mice treated with USP24-i-101 (**D**) was studied via IHC. The level of α-SMG and collagen in ND- and HFD-fed mice with or without USP24 functional knockout (**E**) or USP24-i-101 treatment (**F**) were determined using IHC and Masson’s trichome staining. The expression of USP24, p300, PPARγ in MAFL clinical cohorts was determined via IHC (**G**). The expression of USP24, COX2 and αSMA in the MASH patients was measured via IHC, and the signals were divided into low and high expression. The relationship between USP24 expression and different stages of MASH is shown (**H**, **a**, **b**), and the correlations among the expression of USP24, COX2 and αSMA in the different stages of MASH are shown (**H**, **a**, **c**). Working model—PKA-mediated upregulation of USP24 during lipogenesis increases CREB1 phosphorylation and stabilizes p300. This promotes PPARγ and C/EBPβ gene expression, leading to adipogenesis and fatty liver. FFA treatment increases USP24 expression in the liver, promoting inflammation and fibrosis through activating the NF-κB and TGF-β signaling pathways (**I**)

The expression of the lipogenesis-related proteins PLIN2, PPARγ, CEBPβ and SREBP1 was decreased in USP24-i-101-treated mice, indicating that USP24-i-101, which targets USP24, inhibits lipid formation (Fig. 8A). Free fatty acid (FFA) treatment increased lipid droplet formation in primary hepatocytes, which were inhibited by USP24-i-101 (Fig. 8B). The expression of NF-κB and COX2 was decreased in USP24 knockout mice (USP24^C1695A^) (Fig. 8C) and USP24-i-101-treated HFD-fed mice (Fig. 8D), indicating that targeting USP24 inhibits inflammation. Additionally, the expression of αSMA and collagen was significantly decreased in USP24^C1695A^- (Fig. 8E) or USP24-i-101-treated HFD-fed mice (Fig. 8F), implying that the loss of USP24 represses fibrosis in the livers of HFD-fed mice. Lastly, the relationships among the expression of USP24, p300, PPARγ, COX2 and αSMA in metabolic associated fatty liver (MAFL) cohorts were studied (Fig. 8G, H, Suppl. Table 6 and Suppl. Figure 17). The data showed strong correlations between USP24 and PPARγ (8/8; 100%) and between USP24 and p300 (7/8; 87.5%) (Fig. 8G). We also studied USP24 and PKA-Cα expression in clinical cohorts with obesity using the TCGA database (Fig. 8H). There was a positive correlation between the protein expression and localisation of USP24 and PKA-Cα (Fig. 8H). Furthermore, USP24 expression was upregulated from stage 3 to stage 4 in most MASH patients (Fig. 8I, a, b). A highly positive correlation among USP24, COX2 and αSMA expression was also found in patients of the MASH clinical cohort whose disease was at different stages (Fig. 8I, c, Suppl. Figure 17). These findings suggest that USP24 and p300 may together regulate PPARγ, COX2 and αSMA, leading to fatty liver, inflammation and fibrosis. Since MAFLD/MASH is associated with a high risk of HCC, HCC cohorts from the TCGA database revealed that patients with higher USP24 expression had poor prognosis (p = 0.0024), indicating that higher USP24 expression in MASH patients might also be correlated with a high risk of HCC (Suppl. Figure 18).

## Discussion

Functional disruption of USP24 and targeting USP24 by USP24-i-101 reduces body weight in mice, inhibits lipogenesis through decreased PKA-Cα and p300, thereby suppressing CREB phosphorylation, leading inhibition of adipogenesis. USP24-i-101 decreases FFA to inhibit USP24 expression, thereby impeding NF-κB and TGF-β signaling pathways, leading to the suppression of inflammation and fibrosis during MASH progression (Fig. 8J).

Most research on USP24 focuses on cancer progression and neurodegenerative diseases [4]. This study first demonstrated that USP24 is involved in lipogenesis both in vitro and in vivo. We utilized USP24^C1695A^ mice and USP24-i-101-targeting USP24 mice to investigate the role of USP24 in visceral and liver fat accumulation, as well as using adipocytes and primary hepatocytes to study the molecular mechanism by which USP24 regulates lipogenesis. Previous studies have shown that USP22 stabilizes PPARγ in hepatocellular carcinoma to regulate lipidome accumulation. Previous studies indicated that the PKA/CREB/C/EBPβ/PPARγ axis is directly involved in adipocyte differentiation [35]. Here, we found that USP24 was significantly increased during the early stage of adipocyte differentiation, making it an optimal target for USP24-i-101 intervention. Additionally, we discovered that PKA activation is involved in USP24 upregulation. Second, USP24 upregulation not only increases CREB1 phosphorylation but also stabilizes p300, which can be recruited into CREB to enhance its transcriptional activity in regulating the expression of various lipogenesis-related genes [36]. Previous studies have indicated that several kinases, including PKA, Akt and MAPK, are involved in the phosphorylation [37]. In this study, PKA-mediated USP24 upregulation may stabilize these kinases to increase CREB phosphorylation. Furthermore, previous studies have also shown that CREB1 can be acetylated, thereby recruiting p300/CBP to enhance the transcriptional activity of CREB1. Our previous studies also indicated that p300 is a substrate of USP24 [31, 38].

Previous research has shown that autophagy is involved in lipogenesis [16]. Our recent findings also demonstrated that USP24-i-101 significantly induces autophagy. During adipocyte differentiation, LC3B levels decrease but increase under USP24-i-101 treatment. We attempted to investigate the role of autophagy in adipocyte differentiation by treating with the autophagy inhibitor bafilomycin A1 and the autophagy activator rapamycin. Both activation and inhibition of autophagy blocked adipocyte differentiation, suggesting that autophagy activation and inhibition are required at specific stages of adipocyte differentiation. According to previous studies, autophagy is induced in response to obesity [38]. USP24-i-101 targeting USP24-induced autophagy may also be involved in obesity. The underlying mechanisms require further investigation. In this study, USP24-i-101 targeting USP24 not only inhibited lipid droplet accumulation in the liver but also significantly reduced subcutaneous (SAT) fat and visceral (VAT) body fat around organs, including inguinal WAT (iWAT) and epididymal (eWAT). Previous studies have indicated that visceral fat is associated with several diseases such as cardiovascular diseases [39] and inflammation-related diseases [40]. Herein, we provided in vivo data supporting that treating with USP24-i-101 during the transition from nonobese to obese by HFD or after obesity can inhibit SAT and VAT body fat accumulation. Based on previous studies, VAT adipocytes are more metabolically active and more sensitive to insulin resistance than SAT adipocytes [41]. Whether USP24 is also involved in the trans-differentiation of white adipocytes into brown adipocytes needs to be studied in the future.

Previous research has suggested that lipogenesis and autophagy are involved in several diseases, including MASH, diabetes, cardiovascular disease, and neurodegenerative diseases [16, 42, 43]. Several studies have shown that single nucleotide polymorphisms (SNPs) of USP24 are correlated with Parkinson’s disease [44]. Moreover, recent findings indicate that USP24 is a negative regulator of autophagy. Knockdown of USP24 can improve neurite extension or maintenance in aged iPSC-derived dopaminergic neurons [4], implying that USP24-i-101 targeting USP24 may be effective in inhibiting neurodegenerative diseases. Further related experiments will be conducted to study this issue in the future. Additionally, many studies support that lipogenesis is the crucial factor causing MASH and diabetes [45]. In this study, we discovered that the knockout of USP24 reduced inflammation and fibrosis in the liver, suggesting that USP24-i-101 may inhibit MASH. We also used ultrasound and MRI to study the signal of fat accumulation inside mice and found that fat accumulation was significantly inhibited in USP24^C1695A^ mice or those treated with USP24-i-101 targeting USP24. However, 0.5–1.0 μM USP24-i-101 was effective in HFD-fed male mice, while 10 μM USP24-i-101 was effective in HFD-fed female mice, suggesting that estrogen might be involved in fat accumulation in female mice. Many studies have reported a relationship between estrogen and obesity in women [46]. Most metabolism-related studies used male mice to avoid interference from estrogen. However, we used both male and female mice to study the role of USP24 in lipogenesis. Indeed, the effect of USP24-i-101 targeting USP24 on male and female mice is different. Additionally, blood glucose levels were slightly decreased in USP24^C1695^^A^ mice and those treated with USP24-i-101 targeting USP24. When we checked insulin expression, insulin expression was still increased in HFD-fed mice, implying that insulin tolerance has not yet been achieved. To study the effect of USP24-i-101 on diabetes, an intact type 2 diabetes animal model needs to be established in the future.

Most of the lipogenesis and metabolism-related genes were downregulated in USP24^C1695A^ mice and those treated with USP2-i-101 targeting USP24. Additionally, many other genes were also regulated. Genes involved in ribonucleotide metabolic processes were downregulated by USP24-i-101 treatment; these processes include the chemical reactions and pathways involving a ribonucleotide [47]. Recent studies have indicated that inhibiting proline biosynthesis and lipogenesis synergistically suppressed tumor growth [48], implying that USP24-i-101 targeting USP24 may be effective in inhibiting cancer progression. Other types of genes involved in lysosomes and lytic vacuoles were also downregulated in USP24^C1695A^ mice. The lysosome is a metabolic center. Recent evidence indicates that mTORC1 recruitment to the lysosome responds to nutrient-replete states [49]. The other mechanism related to lysosome-mediated metabolism involves SREBPs, which are provided by lipin1 [50]. In mammals, lipin1 is an mTORC1 substrate that suppresses the activity of the SREBPs. Inactivation of mTORC1 protects mice from lipid accumulation in the liver caused by a HFD, but this protective effect was largely abolished upon knockdown of lipin1 [50]. In this study, several lipin1-like genes, such as Mup3, Mup15, Mup16, Mup1 and Mup12, were upregulated under USP24-i-101 treatment, suggesting that USP24 may regulate lipogenesis by regulating major urinary protein (Mup) gene expression. Previous studies have shown that the upregulation of Mups inhibits hepatic steatosis [33], suggesting that targeting USP24 with USP24-i-101 may be beneficial for preventing hepatic steatosis. Another study also indicated that MUP1 was regulated by nutritional and metabolic signals. The expression of hepatic MUP1 was significantly decreased in both genetic and dietary fat-induced type 2 diabetes, implying that a reduction in MUP1 contributes to hyperglycemia, insulin resistance, and glucose intolerance [34]. Additionally, previous studies have revealed that cytochrome P450 genes, including the Cyps genes Cyp2b10, Cyp2c29, Cyp3a11 and Cyp3a16, were increased, and Hsd3b2 and Hsd3b5 were decreased during lipogenesis [51]. In this study, we found that Cyp3a11, Cyp4a10, Cyp2a22, Cyp26b1 and Cyp2b9 were downregulated, and Hsd3b5 was upregulated in mice treated with USP24-i-101 targeting USP24, suggesting that USP24-i-101 plays a role in preventing lipogenesis. Apart from the role of USP24 in adipogenesis, this study also clarifies the effect of USP24 on inflammation and fibrosis. In vivo studies showed that many inflammation- and fibrosis-related pathways were down regulated by USP24 knockout. In vitro studies found that numerous inflammation- and fibrosis-related genes were downregulated in primary hepatocytes and hepatic stellate cells. Previous research indicated that NF-κB and JNK-State pathways positively regulate inflammation and fibrosis during MAFLD’s progression [52]. This study discovered that NF-κB was dramatically decreased in USP24 knockout mice, USP24-i-101-treated cells, and USP24 knockdown cells, thereby inhibiting NF-κB activation and leading inhibition of inflammation. Our previous study also revealed that p300 is one of the substrate of USP24 to regulate NF-κB expression in lung cancer cells [53]. Therefore, targeting USP24 with USP24-i-101 may destabilize p300 to decrease NF-κB expression, subsequently inhibiting inflammation and leading to fibrosis during MAFLDs progression.

In this study, the β-oxidation of fatty acids and lipogenesis were concurrently suppressed in USP24^C1695A^ mice. PPARγ is involved in lipogenesis and β-oxidation and is inhibited by USP24-i-101 treatment [54]. However, other factors such as PPARα and PPARδ, are also related to β-oxidation. However, the effect of USP24-i-101 treatment on the levels of PPARα and PPARδ remains unknown and needs to be addressed in the future [54]. Additionally, we found that the level of secreted phosphoprotein 1 (SPP1), which is involved in the progression of many cancers, was dramatically decreased in USP24^C1695A^ mice. However, the role of SPP1 in lipogenesis is unclear and requires further clarification. Fatty acid β-oxidation is the process by which fatty acids are broken down to generate acetyl-CoA, which subsequently enters the citric acid cycle to produce energy [55]. β-oxidation occurs in the matrix of mitochondria, and lipogenesis occurs in the cytoplasm of liver cells and adipocytes [56]. Furthermore, the antigen processing and presentation genes were downregulated in USP24^C1695A^ mice, implying that USP24 might be involved in the immune response. How USP24 regulates the immune system will be studied in the future. Additionally, in this study, targeting USP24 showed consistent effect on lipogenesis in primary hepatocytes and in vivo but not in cancer cell lines, HCC cells, and Huh7 cells. This implies that the regulation of lipogenesis changes during cellular immortalization and tumorigenesis, which will be clarified in the future. Finally, other disease models, such as type 2 diabetes and neurodegenerative diseases, will be used to study the effect of targeting USP24 with USP24-i-101 on the inhibition these diseases.

## Conclusion

USP24 upregulation stabilizes PKA-Cα and p300 to activate related pathways involved in lipid metabolism, inflammation, and fibrosis, making it a potential therapeutic target for metabolic disorders such as obesity and Metabolic Associated Fatty Liver Disease (MAFLD). Targeting USP24 with USP24-i-101 can alleviated MAFLD through the modulation of key signaling pathways involved in lipogenesis, inflammation, and fibrosis.

## Supplementary Information

Supplementary material 1: Table 1. Lipid metabolism-related gene expression profile regulated by USP24 knockout in HFD-fed female mice (A) and HFD-fed male mice (B). Table 2. Inflammation-related gene expression profile regulated by USP24 knockout in HFD-fed female mice (A) and HFD-fed male mice (B). Table 3. Fibrosis-related gene expression profile regulated by USP24 knockout in HFD-fed female mice (A) and HFD-fed male mice (B). Table 4. All the sequences of the primers used in this study are listed. Table 5. All the information of primary antibodies used here are listed. Table 6. The information of the MASH patients are included in this study.

Supplementary material 2: Fig. 1. Functional knockout of USP24 expression by CRISPR/Cas9. The cysteine residue at position 1695 of USP24 was altered to alanine (C1695A) by CRISPR/Cas9. The Bbs I restriction enzyme site was deleted, and a new site Nar I was introduced for genotyping (A). Mouse genotypes were determined via PCR and digestion with the Nar I restriction enzyme (B). The sizes of USP24^WT^ and USP24^C1695A^ mice 7 days and 2 months after birth (C). Food intake of USP24^WT^ and USP24^C1695A^ mice is depicted in (D). Blood glucose levels (E) of USP24^WT^ and USP24^C1695A^ mice fed a ND or HFD are shown. (F). Fat content inside HFD-fed mice with or without function knockout mice (USP24^WT^ & USP24^C1695A^) was determined using an ultrasound machine. Fig. 2. The effect of USP24 knockout in HFD-fed mice . The pathology of ND-fed, HFD-fed and USP24-i-101-treated HFD-fed mice was examined via H&E staining (A). The levels of insulin in USP24^WT^ and USP24^C1695A^ mice fed a HFD were determined by IHC (B). The pathology of the pancreas in USP24^WT^ and USP24^C1695A^ mice with or without a HFD was studied by H&E staining (C). Fig. 3. USP24-i-101, which targets USP24, does not significantly inhibit lipogenesis in immortalized hepatocytes. Huh7 hepatocyte cancer cells were treated with FFAs with or without USP24-i-101 treatment. Cell morphology was observed (A), and expression of lipogenesis-related proteins was measured via IB with antibodies against the indicated proteins (B). Fat content inside the cells was measured via oil red O staining (C). Fig. 4. Effect of USP24 knockout and USP24-i-101 on HFD-fed mice. Food intake was comparable among ND-fed, HFD-fed and HFD/USP24-i-101 mice (A). All the mice and their organs, such as the livers of HFD-fed mice, were treated with various doses of USP24-i-101 (B). Primary hepatocytes cultured from USP24^WT^ and USP24^C1695A^ (USP24KO) mice were used to study the effect of USP24 on FFA-induced lipid accumulation via oil red O staining (C). Fig. 5. USP24-i-101 induces autophagy in hepatocytes and adipocytes. The expression of ULK and LC3B in Huh7 (A) and HepG2 (B) cells was determined by IB. The expression of LC3B in Huh7 and HepG2 cells treated with DMSO, bafilomycin A1 (Baf-A1), rapamycin or USP24-i-101 treatment was determined by IF (C). The expression of autophagy-related proteins (D) and lipogenesis-related proteins (E) was determined via protein array and IB, respectively. The mRNA expression of EP300 during adipogenesis under USP24 knockdown was studied by qPCR; NS = not significantly different (F). 3T3-L1 cells were treated with DMSO or 10μM USP24-i-101 for 2 days or 4 days, and then the total RNA was isolated to measure the mRNA level by qPCR (G). 3T3-L1 cells were differentiated into adipocytes with or without 10 μM rp-cAMPs and 10 μM USP24-i-101 treatment, and then the cell morphology was taken (H). Fig. 6. All the results related to the quality control of the RNA-seq data are shown in Fig.3 - Error rate distribution (A), base content distribution (B), Trimmomatic results (C), distribution of gene expression levels (D), Pearson correlation coefficient (E), summary of the Trimmomatic Trim results (F) and TPM log-normalized counts sample PCA (G). Fig. 7. Systemic gene expression by RNA-seq in the livers of USP24^C1695A^ and USP24^WT^ HFD-fed mice. The detailed experimental design and bioinformatics tools used to study the systemic gene expression by RNA-seq in the livers of USP24^C1695A^ and USP24^WT^ HFD-fed mice (A). NGS data from the livers of ND-fed and HFD-fed mice with or without USP24 knockout (USP24^WT^ & USP24^C1695A^) were analyzed with the following GSEA gene set: MousePath_Pathway_gmt Versus Matrix Volcano Plot (B, C). Fig. 8. NGS data from the livers of HFD-fed mice with or without USP24 knockout (HFD_USP24^C1695A^/HFD_USP24^WT^) were analyzed with DisGeNET_All_cnetplot_circo. Fig. 9. Systemic gene expression by RNA-seq in the livers of USP24^C1695A^ and USP24^WT^ HFD-fed mice. The NGS data of HFD-fed mice with or without USP24 knockout (HFD_USP24^C1695A^/HFD_USP24^WT^) or USP24-i-101-treated HFD-fed mice were analyzed via bioinformatics tools; Top 30 ALL GO enrichment terms: Up vs. Down Gene Counts (A; HFD_USP24^C1695A^/HFD_USP24^WT^), DEG Down DisGeNET Enrichment Pathway (B; HFD_USP24^C1695A^/HFD_USP24^WT^), heatmap plot (C; 1 mg/KG USP24-i-101, i.p., two times per week), DEG All GO Dotplot (D; 1 mg/KG USP24-i-101, i.p., two times per week) and DEG Up ALL GO Dotplot (E; 1 mg/KG USP24-i-101, i.p., two times per week). Fig. 10. Systemic gene expression by RNA-seq in the livers of USP24^C1695A^ and USP24^WT^ HFD-fed mice - The NGS data of HFD-fed mice with or without USP24 knockout (HFD_USP24^C1695A^/HFD_USP24^WT^) were analyzed by bioinformatics tools; DEG Down GO TERMs – BP Enrichment (A), DEG Down DisGeNET Enrichment Pathway (B) and DEG Down GO TERMs – CC Enrichment (C). Fig. 11. Systemic gene expression by RNA-seq in the livers of USP24^C1695A^ and USP24^WT^ HFD-fed mice - The NGS data of HFD-fed mice with or without USP24 knockout (HFD_USP24^C1695A^/HFD_USP24^WT^) were analyzed by bioinformatics tools; DEG Down KEGG Enrichment Pathway (A), DEG Down DO Enrichment Pathway (B). Fig. 12. Expression of numerous genes related to lipogenesis is regulated by USP24 *in vivo* - NGS data from the livers of HFD-fed mice with or without USP24 knockout (HFD_USP24^C1695A^/HFD_USP24^WT^) were analyzed with a heatmap; MM_FATTY_ACID_BETA_OXIDATION-WP143 heatmap (A), WIKIPATHWAYS_MM_ADIPOGENESIS-WP236 heatmap (B), GLYCOLYSIS_AND_GLUCONEOGENESIS-WP534 heatmap (female mice (C, a)) and male mice (C, b). Fig. 13. USP24-i-101 targeted USP24 in HFD-fed mice. The pathology of all the USP24-i-101-treated mice was examined via H&E staining (A), and fat accumulation was also determined via oil red O staining assay (B). Fig. 14. Systematic gene expression under USP24-i-101 treatment. RNA samples isolated from liver organs of HFD-fed mice with or without USP24-i-101 treatment were used to study the global gene expression profiles via RNA-seq, and the quality of the NGS data was subsequently analyzed by (A) error rate distribution (B). Base content distribution. (C) Trimmomatic results (D). Distribution of gene expression levels (E). Pearson correlation coefficient (F). Summary of the Trimmomatic Trim Results. Fig.15. USP24 promotes inflammation- and fibrosis-related protein expression - All the USP24-mediated fibrosis-related pathways identified from the NGS data of HFD-USP24^WT^ and HFD-USP24^C1695A^ mice were analyzed via the DEG Down ALL GO Dotplot (A). The number of macrovesicular steatotic cells in ND- and HFD-fed mice with or without USP24 knockout was counted (B). AML12 cells were treated with 1μg/ml LPS or 10 μM USP24-i-101 for 24 h, and the mRNA expressions of α-SMA, Col1a1 and TGFβ1 were measured via qPCR (C). Fig. 16. Hepatocytes with macrovesicular steatosis were analyzed by software. Fig.17. All the expression of USP24, COX2 and αSMA in the clinical MASH cohorts was measured via IHC. The inconsistent results between USP24 and αSMA or USP24 and COX2 are highlighted with squares. Fig.18. The relationship between USP24 expression and the survival rate of patients with HCC in the TCGA cohort was analyzed.

## Data Availability

All data generated or analyzed during this study are included in this published article and its supplementary information files.
