# Supplementary material for: USP24 upregulation stabilizes PKA-Cα to promote lipogenesis, inflammation, and fibrosis during MASH progression
Source: J Biomed Sci. 2025 May 30;32:54. doi: 10.1186/s12929-025-01148-4 (PMC12125897; doi:10.1186/s12929-025-01148-4)
Supplement: Supplementary file 1 — Supplementary material 1: Table 1. Lipid metabolism-related gene expression profile regulated by USP24 knockout in HFD-fed female mice (A) and HFD-fed male mice (B). Table 2. Inflammation-related gene expression profile regulated by USP24 knockout in HFD-fed female mice (A) and HFD-fed male mice (B). Table 3. Fibrosis-related gene expression profile regulated by USP24 knockout in HFD-fed female mice (A) and HFD-fed male mice (B). Table 4. All the sequences of the primers used in this study are listed. Table 5. All the information of primary antibodies used here are listed. Table 6. The information of the MASH patients are included in this study. [file 12929_2025_1148_MOESM1_ESM.docx]

**Suppl. Table 1. Gene expression profile of downregulated fatty acid metabolism related gene in USP24 knockout mice liver**

**Female**

| **Gene Symbol** | **Gene Name** | **log_2_ Fold Change** | **P-value** |
| --- | --- | --- | --- |
| Scd1 | Stearoyl-Coenzyme A desaturase 1 | -5.672238424 | 1.8349E-291 |
| Acox1 | Acyl-Coenzyme A oxidase 1, palmitoyl | -1.259237248 | 3.1786E-108 |
| Acaa1b | Acetyl-Coenzyme A acyltransferase 1B | -1.652375081 | 3.8039E-101 |
| Apoa4 | Apolipoprotein A-IV | -1.887862194 | 2.89428E-46 |
| Lpin2 | Lipin 2 | -1.774673985 | 3.1901E-43 |
| Ehhadh | Enoyl-Coenzyme A, hydratase/3-hydroxyacyl Coenzyme A dehydrogenase | -1.311252812 | 9.83728E-33 |
| Acot1 | Acyl-coA thioesterase 1 | -2.535971451 | 1.73311E-24 |
| Acot4 | Acyl-coA thioesterase 4 | -1.11961777 | 4.10397E-24 |
| Por | P450 (cytochrome) oxidoreductase | -1.128977135 | 1.51578E-23 |
| Cyp2c29 | Cytochrome P450, family 2, subfamily c, polypeptide 29 | -1.292957491 | 5.12777E-19 |
| Cyp2f2 | Cytochrome P450, family 2, subfamily f, polypeptide 2 | -1.487838089 | 1.6904E-17 |
| Acot3 | Acyl-coA thioesterase 3 | -1.398131145 | 1.83244E-16 |
| Lpin1 | Lipin 1 | -1.843327633 | 1.10712E-14 |
| Cd74 | CD74 antigen | -1.679434801 | 3.55712E-14 |
| Acot2 | Acyl-coA thioesterase 2 | -2.149109474 | 4.52791E-14 |
| Decr1 | 2,4-dienoyl coA reductase 1, mitochondrial | -1.006101148 | 2.29158E-10 |
| Cd36 | CD36 molecule | -1.154663453 | 7.66569E-10 |
| Acacb | Acetyl-Coenzyme A carboxylase beta | -1.767182388 | 3.49067E-08 |
| Pdk4 | Pyruvate dehydrogenase kinase, isoenzyme 4 | -3.136788458 | 1.08437E-07 |
| Plin5 | Perilipin 5 | -1.654529799 | 1.33985E-07 |
| Crat | Carnitine acetyltransferase | -1.134341339 | 2.407E-06 |
| Gpat4 | Glycerol-3-phosphate acyltransferase 4 | -1.181867343 | 5.57755E-06 |
| Acads | Acyl-Coenzyme A dehydrogenase, short chain | -1.200073647 | 0.000285512 |
| Eif6 | Eukaryotic translation initiation factor 6 | -1.615398223 | 0.001320967 |

Male

| **Gene Symbol** | **Gene Name** | **log_2_ Fold Change** | **P-value** |
| --- | --- | --- | --- |
| Apoa4 | Apolipoprotein A-IV | -5.541273467 | N/A |
| Fabp1 | Fatty acid binding protein 1, liver | -1.012892483 | 2.2088E-165 |
| Scd1 | Stearoyl-Coenzyme A desaturase 1 | -1.136874311 | 1.3469E-159 |
| Lpin2 | Lipin 2 | -2.056625312 | 1.77829E-54 |
| Acaa1b | Acetyl-Coenzyme A acyltransferase 1B | -1.052768729 | 2.31902E-48 |
| Apoa5 | Apolipoprotein A-V | -1.307940074 | 1.15175E-46 |
| Cpt1a | Carnitine palmitoyl transferase 1a, liver | -1.784837837 | 4.08873E-46 |
| Elovl5 | ELOVL family member 5, elongation of long chain fatty acids (yeast) | -1.696063405 | 5.17184E-38 |
| Aldh3a2 | Aldehyde dehydrogenase family 3, subfamily A2 | -1.132251157 | 1.47791E-37 |
| Lpin1 | Lipin 1 | -3.013993504 | 7.09396E-34 |
| Fads2 | Fatty acid desaturase 2 | -1.462542481 | 4.60055E-33 |
| Cyp2a22 | Cytochrome P450, family 2, subfamily a, polypeptide 22 | -1.705063972 | 2.06258E-21 |
| Fabp2 | Fatty acid binding protein 2, intestinal | -1.731593152 | 4.92272E-20 |
| Cyp2b9 | Cytochrome P450, family 2, subfamily b, polypeptide 9 | -1.191488207 | 6.85553E-17 |
| Cd36 | CD36 molecule | -1.283808504 | 5.92805E-14 |
| Cyp2c38 | Cytochrome P450, family 2, subfamily c, polypeptide 38 | -1.291594049 | 9.40352E-14 |
| Fads1 | Fatty acid desaturase 1 | -1.037227313 | 1.85621E-13 |
| Cd74 | CD74 antigen | -1.961395445 | 2.77606E-10 |
| Cyp2b13 | Cytochrome P450, family 2, subfamily b, polypeptide 13 | -3.683580116 | 2.22072E-09 |
| Abhd2 | Abhydrolase domain containing 2 | -1.175487532 | 7.47213E-08 |
| Acot3 | Acyl-coA thioesterase 3 | -1.229095274 | 7.844E-06 |
| Acot2 | Acyl-coA thioesterase 2 | -1.678550909 | 0.000186684 |
| Aacs | Acetoacetyl-coA synthetase | -1.726202509 | 0.000894571 |
| Cyp2a4 | Cytochrome P450, family 2, subfamily a, polypeptide 4 | -2.118023221 | 0.001419774 |

**Suppl. Table 2. Gene expression profile of downregulated inflammatory response gene in USP24 knockout mice liver**

**Female**

| **Gene Symbol** | **Gene Name** | **log_2_ Fold Change** | **P-value** |
| --- | --- | --- | --- |
| Clu | Clusterin | -1.119561657 | 3.02575E-38 |
| Grn | Granulin | -1.060188706 | 2.02843E-15 |
| C1qa | Complement component 1, q subcomponent, alpha polypeptide | -1.953278929 | 2.77052E-08 |
| Ctsc | Cathepsin C | -1.061816561 | 0.00206222 |
| Lbp | Lipopolysaccharide binding protein | -1.432359799 | 3.2852E-09 |
| Rtn4 | Reticulon 4 | -1.569172248 | 2.06865E-08 |
| Gpx1 | Glutathione peroxidase 1 | -1.464815177 | 2.0217E-122 |
| Gpx4 | Glutathione peroxidase 4 | -1.240392113 | 1.68774E-21 |
| Cd5l | CD5 antigen-like | -1.895322391 | 6.17101E-13 |
| Ctss | Cathepsin S | -2.256325987 | 2.5427E-07 |
| Nlrp12 | NLR family, pyrin domain containing 12 | -1.837185667 | 1.92374E-06 |
| Saa1 | Serum amyloid A 1 | -2.545155996 | 2.94951E-33 |
| Saa2 | Serum amyloid A 2 | -2.846295881 | 1.01415E-24 |
| Cd36 | CD36 molecule | -1.154663453 | 7.66569E-10 |

Male

| **Gene Symbol** | **Gene Name** | **log_2_ Fold Change** | **P-value** |
| --- | --- | --- | --- |
| Saa1 | Serum amyloid A 1 | -3.071263879 | 1.56345E-16 |
| Serpina3n | Serine (or cysteine) peptidase inhibitor, clade A, member 3N | -1.110682799 | 2.27241E-12 |
| Saa2 | Serum amyloid A 2 | -2.957042400 | 2.43174E-08 |
| Cd5l | CD5 antigen-like | -2.390636564 | 5.1963E-07 |
| Orm2 | Orosomucoid 2 | -1.490894678 | 0.000151873 |
| Grn | Granulin | -1.059267981 | 3.18888E-13 |
| C1qa | Complement component 1, q subcomponent, alpha polypeptide | -2.140147685 | 0.000881897 |
| Stap1 | Signal transducing adaptor family member 1 | -4.234990057 | 0.004498586 |
| Ctss | Cathepsin S | -2.290461642 | 0.000416776 |
| Cd81 | CD81 antigen | -1.025923896 | 5.03479E-15 |
| Cd36 | CD36 molecule | -1.283808504 | 5.92805E-14 |
| Rtn4 | Reticulon 4 | -1.412242105 | 0.001043124 |
| Saa1 | Serum amyloid A 1 | -3.071263879 | 1.56345E-16 |

**Suppl. Table 3. Gene expression profile of downregulated fibrosis response gene in the liver of USP24 knockout HFD fed mice**

**Female**

| **Gene Symbol** | **Gene Name** | **log_2_ Fold Change** | **P-value** |
| --- | --- | --- | --- |
| Abcd3 | ATP-binding cassette, sub-family D, member 3 | -0.79543399 | 5.23097E-10 |
| Lipa | Lysosomal acid lipase A | -0.672520545 | 0.000589433 |
| Asl | Argininosuccinate lyase | -0.372368105 | 0.00251454 |
| Marc1 | Mitochondrial amidoxime reducing component 1 | -0.423613174 | 0.004867896 |

Male

| **Gene Symbol** | **Gene Name** | **log_2_ Fold Change** | **P-value** |
| --- | --- | --- | --- |
| Abcb4 | ATP-binding cassette, sub-family, member 4 | -0.822726179 | 4.23883E-09 |
| Abcd3 | ATP-binding cassette, sub-family, member 3 | -0.649179278 | 7.04961E-08 |
| Lipa | Lysosomal acid lipase A | -0.820167078 | 1.19395E-06 |
| Gpd1 | Glycerol-3-phosphate dehydrogenase 1 | -0.6381114205 | 4.02624E-05 |
| Pygl | Liver glycogen phosphorylase | -0.55230681 | 0.001131151 |
| Taldo1 | Transaldolase 1 | -0.731066238 | 0.003021381 |
|  |  |  |  |

**Suppl. Table 4. Primer Sequences used in this study**

| **Gene** | **Species** | **Forward** | **Reverse** |
| --- | --- | --- | --- |
| GAPDH | Human | 5’-CCATCACCATCTTCCAGGAG-3’ | 5’-CCTGCTTCACCACCTTCTTG-3’ |
| GAPDH | Mouse | 5’-CATCACTGCCACCCAGAAGACTG-3’ | 5’-ATGCCAGTGAGCTTCCCGTTCAG-3’ |
| SREBP1 | Human | 5’-ACTTCTGGAGGCATCGCAAGCA-3’ | 5’-AGGTTCCAGAGGAGGCTACAAG-3’ |
| PPARγ | Human | 5’-AGCCTGCGAAAGCCTTTTGGTG-3’ | 5’-GGCTTCACATTCAGCAAACCTGG-3’ |
| PPARγ | Mouse | 5’-GTACTGTCGGTTTCAGAAGTGCC-3’ | 5’-ATCTCCGCCAACAGCTTCTCCT-3’ |
| Cebpβ | Mouse | 5’-CAACCTGGAGACGCAGCACAAG-3’ | 5’-GCTTGAACAAGTTCCGCAGGGT-3’ |
| USP24 | Mouse | 5’-TTCTGCTCGGATCACCAAGTGC-3’ | 5’-CTGCACATCTGGATGCGCTGAT-3’ |
| CREB1 | Mouse | 5’-AACAATGGTACGGATGGGGT-3’ | 5’-AGGACGCCATAACAACTCCA-3’ |
| Pla2g7 | Human | 5’-CTGCTATTGGCATTGACCTGGC-3’ | 5’-AGGTAGAGCCAAGACTTGTCCC-3’ |
| Ly86 | Human | 5’-AGGCGGCTCTGCCCAAGTTTTC-3’ | 5’-CCACGGTGGACCGTTTTTCAGT-3’ |

**Suppl. Table 5. Primary Antibodies used in this study**

| **Target** | **Cat No.** | **Manufacturer** | **Concentration** |
| --- | --- | --- | --- |
| USP24 | 13126-AP | Proteintech | 1:3000 |
| β-Actin | GTX26276 | Genetex | 1:5000 |
| α-Tubulin | GTX628802 | Genetex | 1:5000 |
| SREBP1 | Ab28481 | Abcam | 1:1000 |
| PPARγ | GTX32803 | Genetex | 1:1000 |
| PLIN1 | GTX634406 | Genetex | 1:1000 |
| PLIN2 | GTX54569 | Genetex | 1:2000 |
| C/EBPβ | GTX64382 | Genetex | 1:1000 |
| CREB | GTX112846 | Genetex | 1:3000 |
| phosphor CREB  at Ser133 | GTX130379 | Genetex | 1:3000 |
| LC3B | 3868S | Cell Signaling | 1:3000 |
| C/EBPδ | Ab245241 | Abcam | 1:1000 |
| RSK2 | GTX104934 | Genetex | 1:2000 |
| CaMKII | GTX637556 | Genetex | 1:2000 |
| CaMKIV | GTX106023 | Genetex | 1:2000 |
| PKA-Cα | GTX104934 | Genetex | 1:1000 |
| COX2 | IR109-446 | iREAL | 1:1000 |
| NF-kB | IRM033 | iREAL | 1:1000 |
| Phosphor-p65 | GTX133899 | Genetex | 1:1000 |
| Vimentin | IR45-137 | iREAL | 1:1000 |
| N-cadherin | IR46-143 | iREAL | 1:1000 |
| E-cadherin | IR55-180 | iREAL | 1:1000 |
| α-SMA | Ab5694 | Abcam | 1:1000 |
| Fibronectin | IR48-138 | iREAL | 1:1000 |
| Collagen | IR292-962 | iREAL | 1:1000 |
| IkBα | GTX110521 | Genetex | 1:1000 |
| Ubiquitin | GTX128826 | Genetex | 1:2000 |
| GFP | SC-9996 | Santa Cruz | 1:3000 |
| P300 | 554215 | BD Biosciences | 1:1000 |

Suppl. Table 6. The information of the clinical cohorts

| Variable | NAS=1,2 (N=13) | NAS=3,4 (N=12) | NAS=5,6 (N=10) | NAS=7,8 (N=4) |
| --- | --- | --- | --- | --- |
| Age (Years) |  |  |  |  |
| Mean (± SEM) | 46.23 (± 3.44) | 36.5 (± 2.12) | 43.2 (± 3.57) | 43 (± 4.74) |
| Gender |  |  |  |  |
| Male | 6 | 5 | 7 | 2 |
| Female | 7 | 7 | 3 | 2 |
| Race/Ethnicity |  |  |  |  |
| Asian | 13 | 12 | 10 | 4 |
| ALT (U/L) |  |  |  |  |
| Mean (± SEM) | 44.08 (± 8.78) | 43.68 (± 5.11) | 68.6 (± 19.97) | 130 (± 23.13) |
| AST (U/L) |  |  |  |  |
| Mean (± SEM) | 30.31 (± 2.88) | 27.68 (± 2.10) | 40.8 (± 9.76) | 99.25 (± 22.75) |
| BMI (kg/m^2^) |  |  |  |  |
| Mean (± SEM) | 34.70 (± 3.05) | 45.42 (± 2.61) | 40.86 (± 2.59) | 30.49 (± 2.49) |
| Fatty liver |  |  |  |  |
| NAS=0 | 0 | 0 | 0 | 0 |
| NAS=1,2 | 13 | 0 | 0 | 0 |
| NAS=3,4 | 0 | 12 | 0 | 0 |
| NAS=5,6 | 0 | 0 | 10 | 0 |
| NAS=7,8 | 0 | 0 | 0 | 4 |

Data are presented as number of patients, mean (± SEM).
