# Supplementary material for: USP24 upregulation stabilizes PKA-Cα to promote lipogenesis, inflammation, and fibrosis during MASH progression
Source: J Biomed Sci. 2025 May 30;32:54. doi: 10.1186/s12929-025-01148-4 (PMC12125897; doi:10.1186/s12929-025-01148-4)
Supplement: Supplementary file 2 — Supplementary material 2: Fig. 1. Functional knockout of USP24 expression by CRISPR/Cas9. The cysteine residue at position 1695 of USP24 was altered to alanine (C1695A) by CRISPR/Cas9. The Bbs I restriction enzyme site was deleted, and a new site Nar I was introduced for genotyping (A). Mouse genotypes were determined via PCR and digestion with the Nar I restriction enzyme (B). The sizes of USP24WT and USP24C1695A mice 7 days and 2 months after birth (C). Food intake of USP24WT and USP24C1695A mice is depicted in (D). Blood glucose levels (E) of USP24WT and USP24C1695A mice fed a ND or HFD are shown. (F). Fat content inside HFD-fed mice with or without function knockout mice (USP24WT & USP24C1695A) was determined using an ultrasound machine. Fig. 2. The effect of USP24 knockout in HFD-fed mice . The pathology of ND-fed, HFD-fed and USP24-i-101-treated HFD-fed mice was examined via H&E staining (A). The levels of insulin in USP24WT and USP24C1695A mice fed a HFD were determined by IHC (B). The pathology of the pancreas in USP24WT and USP24C1695A mice with or without a HFD was studied by H&E staining (C). Fig. 3. USP24-i-101, which targets USP24, does not significantly inhibit lipogenesis in immortalized hepatocytes. Huh7 hepatocyte cancer cells were treated with FFAs with or without USP24-i-101 treatment. Cell morphology was observed (A), and expression of lipogenesis-related proteins was measured via IB with antibodies against the indicated proteins (B). Fat content inside the cells was measured via oil red O staining (C). Fig. 4. Effect of USP24 knockout and USP24-i-101 on HFD-fed mice. Food intake was comparable among ND-fed, HFD-fed and HFD/USP24-i-101 mice (A). All the mice and their organs, such as the livers of HFD-fed mice, were treated with various doses of USP24-i-101 (B). Primary hepatocytes cultured from USP24WT and USP24C1695A (USP24KO) mice were used to study the effect of USP24 on FFA-induced lipid accumulation via oil red O staining (C). Fig. 5. USP24 [file 12929_2025_1148_MOESM2_ESM.pdf]

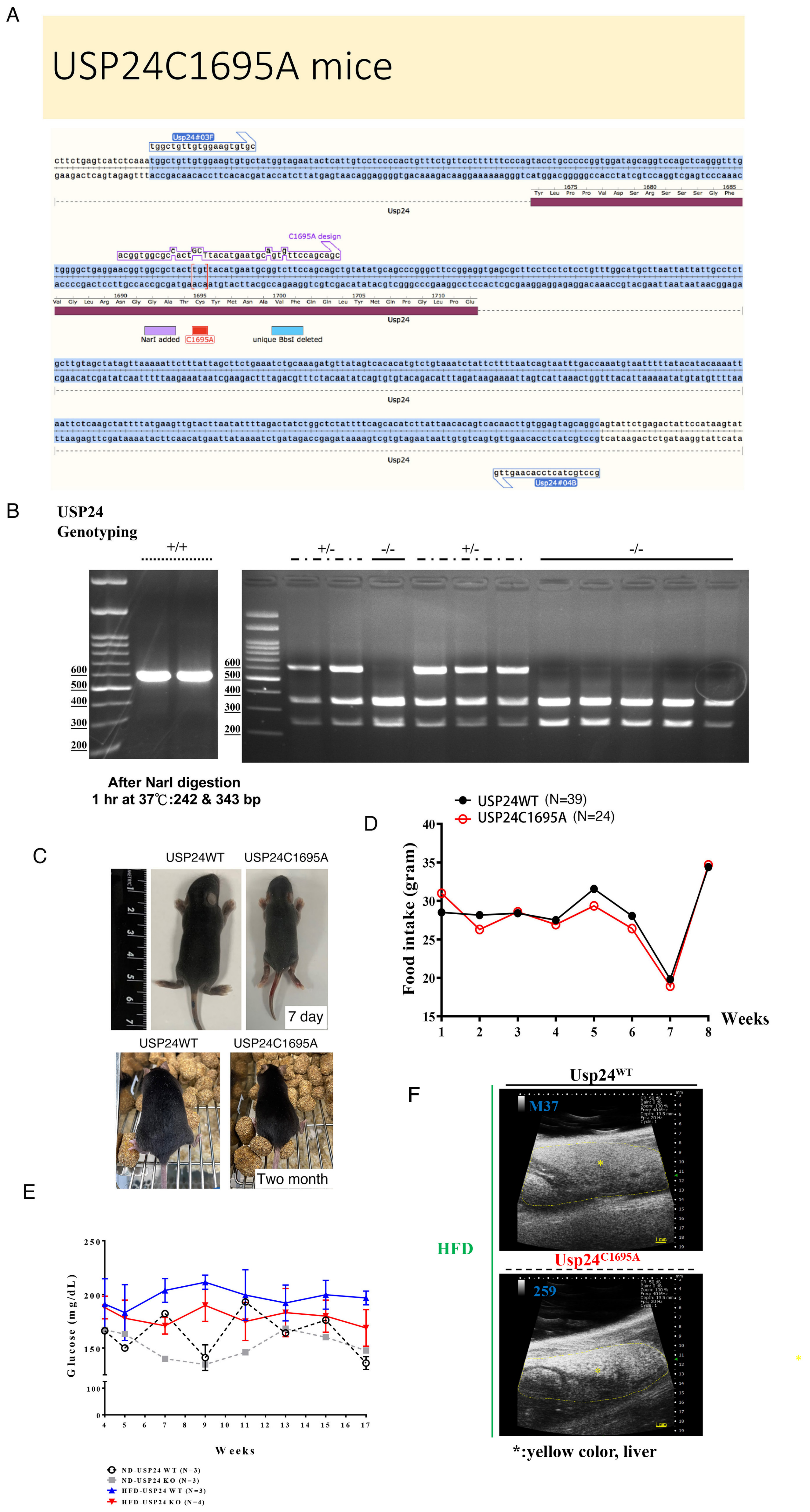
A
USP24C1695A mice
Usp24#03F
C1695A design
NarI added
C1695A
unique BbsI deleted
Usp24
Usp24#04B
B
USP24
Genotyping
+/+
+/-
-/-
+/-
-/-
600
500
400
300
200
After NarI digestion
1 hr at 37℃:242 & 343 bp
C
USP24WT
USP24C1695A
7 day
USP24WT
USP24C1695A
Two month
D
USP24WT (N=39)
USP24C1695A (N=24)
Food intake (gram)
Weeks
E
Glucose (mg/dL)
Weeks
ND-USP24 WT (N=3)
ND-USP24 KO (N=3)
HFD-USP24 WT (N=3)
HFD-USP24 KO (N=4)
F
HFD
Usp24WT
M37
Usp24C1695A
259
*:yellow color, liver

Suppl.Fig.1

Suppl. Fig.2

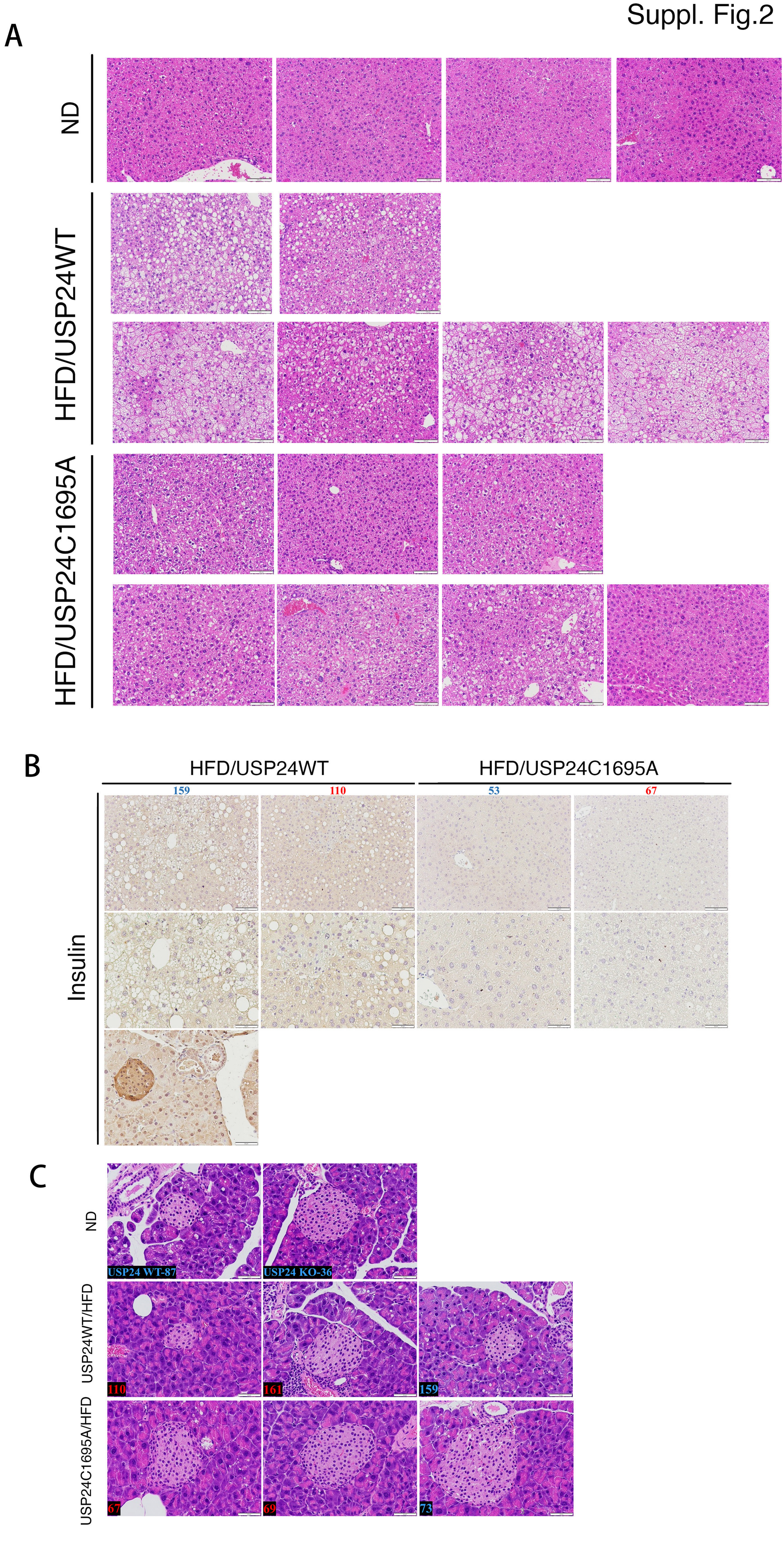
A
ND
HFD/USP24WT
HFD/USP24C1695A
B
HFD/USP24WT
HFD/USP24C1695A
159
110
53
67
Insulin
C
ND
USP24 WT-87
USP24 KO-36
USP24WT/HFD
110
161
159
USP24C1695A/HFD
67
69
73

Huh7

Suppl. Fig.3

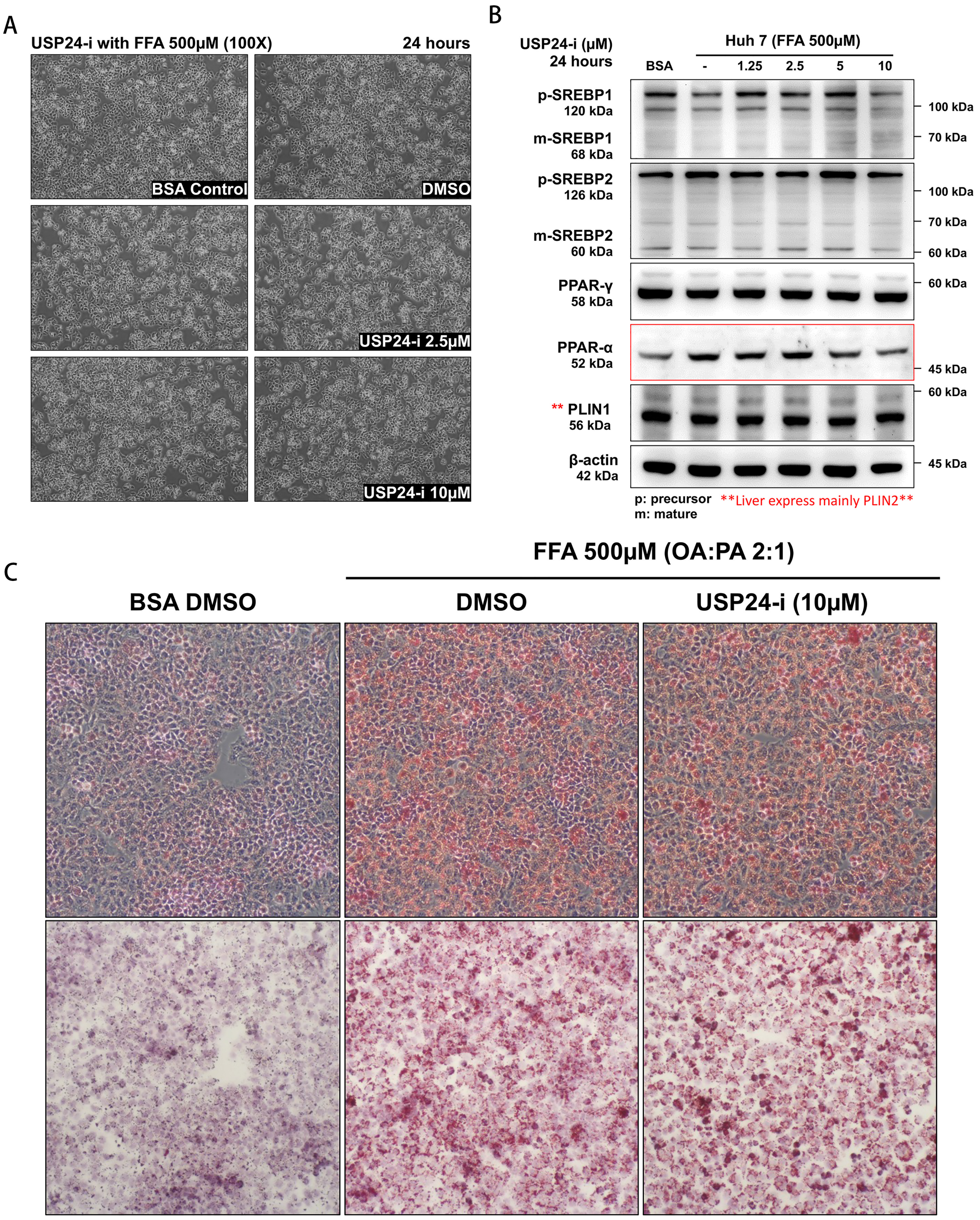

USP24-i = USP24-i-101

Suppl. Fig.4

A

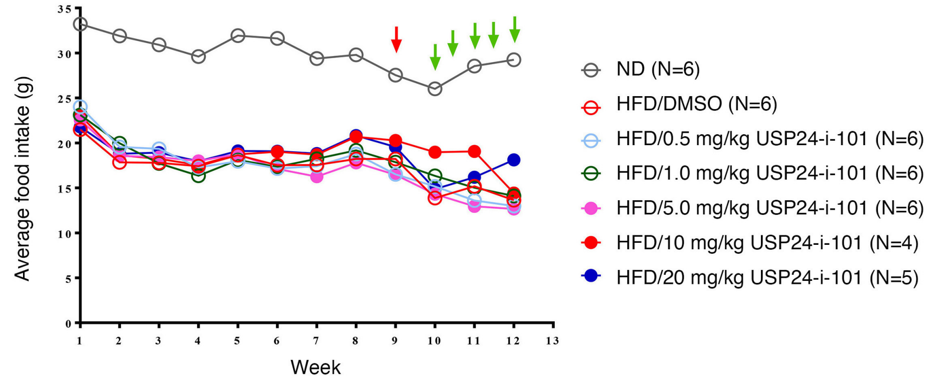

B

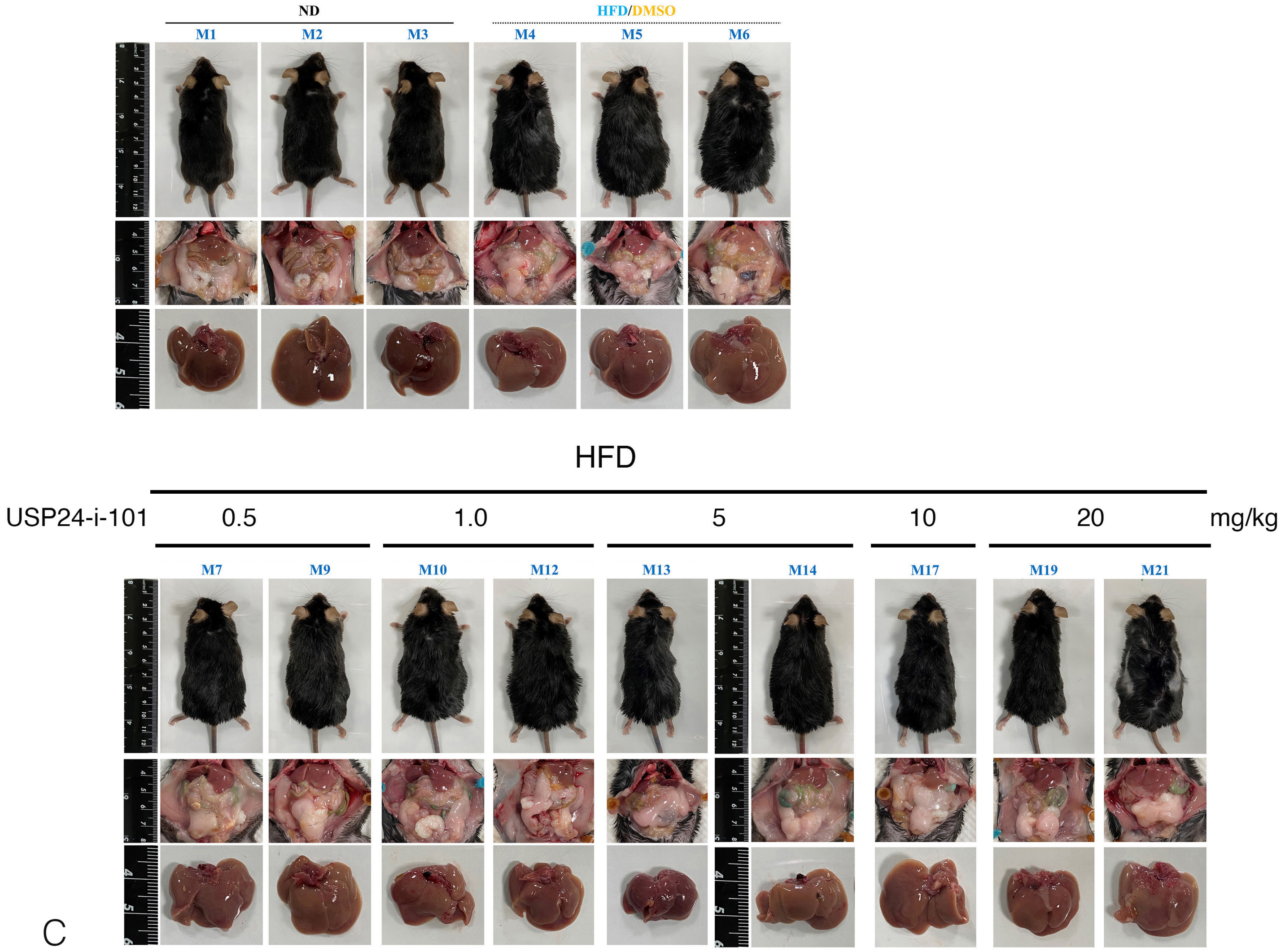

C

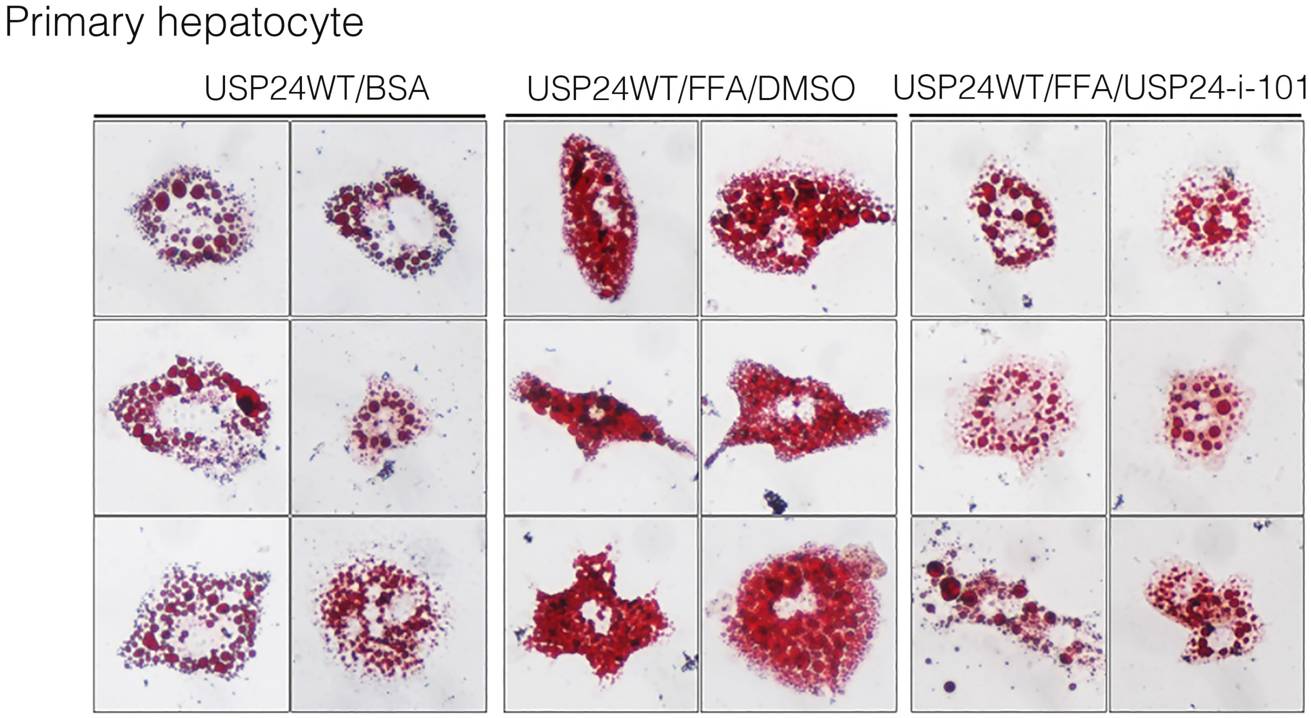

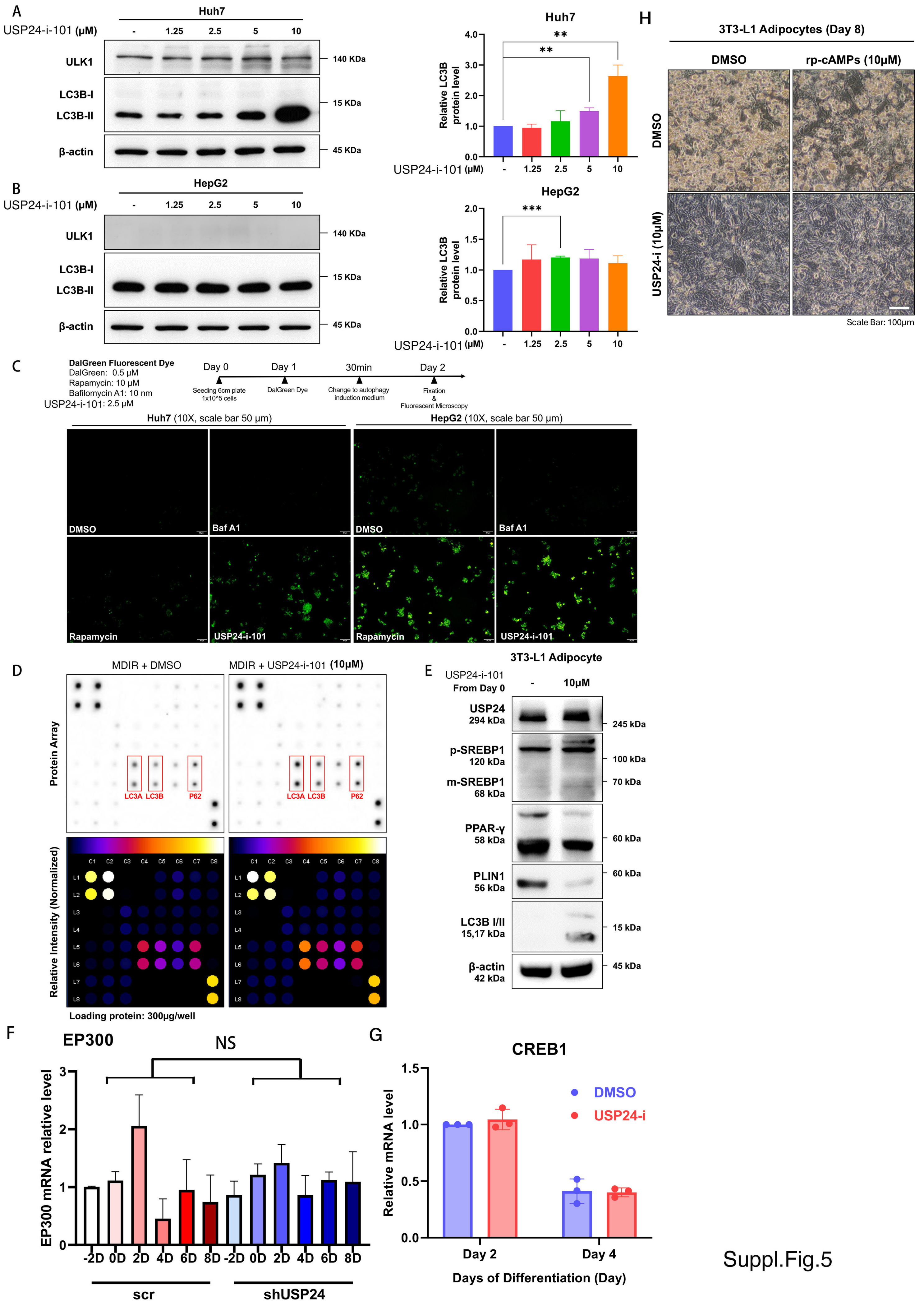
A
Huh7
USP24-i-101 (μM)
- 1.25 2.5 5 10
ULK1
LC3B-I
LC3B-II
β-actin
140 KDa
15 KDa
45 KDa
B
HepG2
Relative LC3B protein level
C
DalGreen Fluorescent Dye
DalGreen: 0.5 μM
Rapamycin: 10 μM
Bafilomycin A1: 10 nm
USP24-i-101: 2.5 μM
Day 0 Day 1 30min Day 2
Seeding 6cm plate 1x10^5 cells
DalGreen Dye
Change to autophagy induction medium
Fixation & Fluorescent Microscopy
Huh7 (10X, scale bar 50 μm)
HepG2 (10X, scale bar 50 μm)
DMSO
Baf A1
Rapamycin
USP24-i-101
D
MDIR + DMSO
MDIR + USP24-i-101 (10μM)
Protein Array
LC3A LC3B P62
Relative Intensity (Normalized)
Loading protein: 300μg/well
E
3T3-L1 Adipocyte
USP24-i-101
From Day 0
- 10μM
USP24 294 kDa
p-SREBP1 120 kDa
m-SREBP1 68 kDa
PPAR-γ 58 kDa
PLIN1 56 kDa
LC3B I/II 15,17 kDa
β-actin 42 kDa
245 kDa
100 kDa
70 kDa
60 kDa
15 kDa
45 kDa
F
EP300
NS
EP300 mRNA relative level
-2D 0D 2D 4D 6D 8D -2D 0D 2D 4D 6D 8D
scr
shUSP24
G
CREB1
DMSO
USP24-i
Relative mRNA level
Day 2 Day 4
Days of Differentiation (Day)
H
3T3-L1 Adipocytes (Day 8)
DMSO
rp-cAMPs (10μM)
USP24-i (10μM)
Scale Bar: 100μm

Suppl.Fig.5

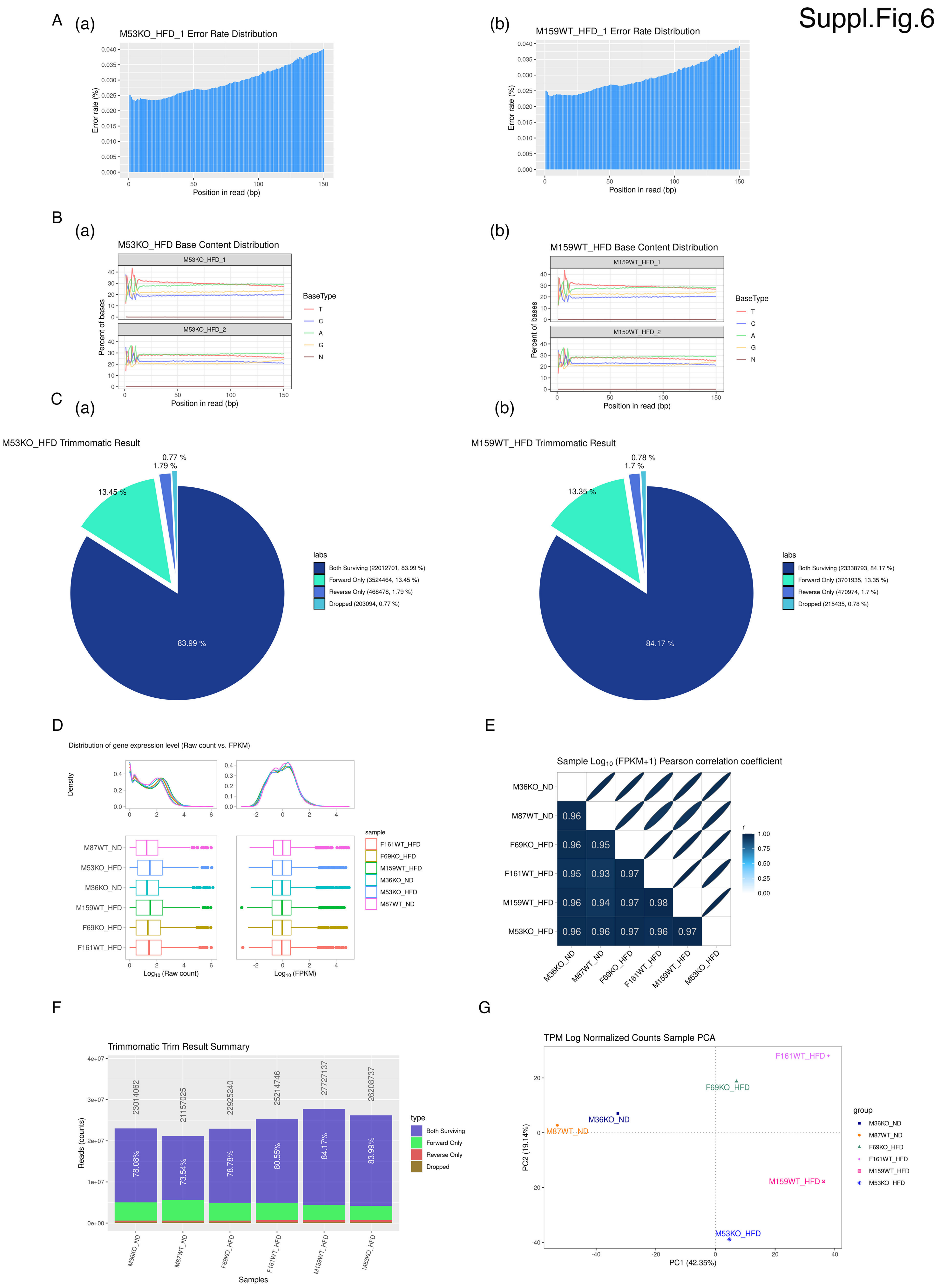

Suppl.Fig.6
A (a)
M53KO_HFD_1 Error Rate Distribution
Error rate (%)
Position in read (bp)
(b)
M159WT_HFD_1 Error Rate Distribution
B (a)
M53KO_HFD Base Content Distribution
M53KO_HFD_1
M53KO_HFD_2
Percent of bases
BaseType
T
C
A
G
N
(b)
M159WT_HFD Base Content Distribution
M159WT_HFD_1
M159WT_HFD_2
C (a)
M53KO_HFD Trimmomatic Result
labs
Both Surviving (22012701, 83.99 %)
Forward Only (3524464, 13.45 %)
Reverse Only (468478, 1.79 %)
Dropped (203094, 0.77 %)
(b)
M159WT_HFD Trimmomatic Result
Both Surviving (23338793, 84.17 %)
Forward Only (3701935, 13.35 %)
Reverse Only (470974, 1.7 %)
Dropped (215435, 0.78 %)
D
Distribution of gene expression level (Raw count vs. FPKM)
Density
sample
F161WT_HFD
F69KO_HFD
M159WT_HFD
M36KO_ND
M53KO_HFD
M87WT_ND
Log10 (Raw count)
Log10 (FPKM)
E
Sample Log10 (FPKM+1) Pearson correlation coefficient
M36KO_ND
M87WT_ND 0.96
F69KO_HFD 0.96 0.95
F161WT_HFD 0.95 0.93 0.97
M159WT_HFD 0.96 0.94 0.97 0.98
M53KO_HFD 0.96 0.96 0.97 0.96 0.97
F
Trimmomatic Trim Result Summary
Reads (counts)
Samples
type
Both Surviving
Forward Only
Reverse Only
Dropped
M36KO_ND 23014062 78.08%
M87WT_ND 21157025 73.54%
F69KO_HFD 22925240 78.78%
F161WT_HFD 25214746 80.55%
M159WT_HFD 27727137 84.17%
M53KO_HFD 26208737 83.99%
G
TPM Log Normalized Counts Sample PCA
PC1 (42.35%)
PC2 (19.14%)
group
M36KO_ND
M87WT_ND
F69KO_HFD
F161WT_HFD
M159WT_HFD
M53KO_HFD

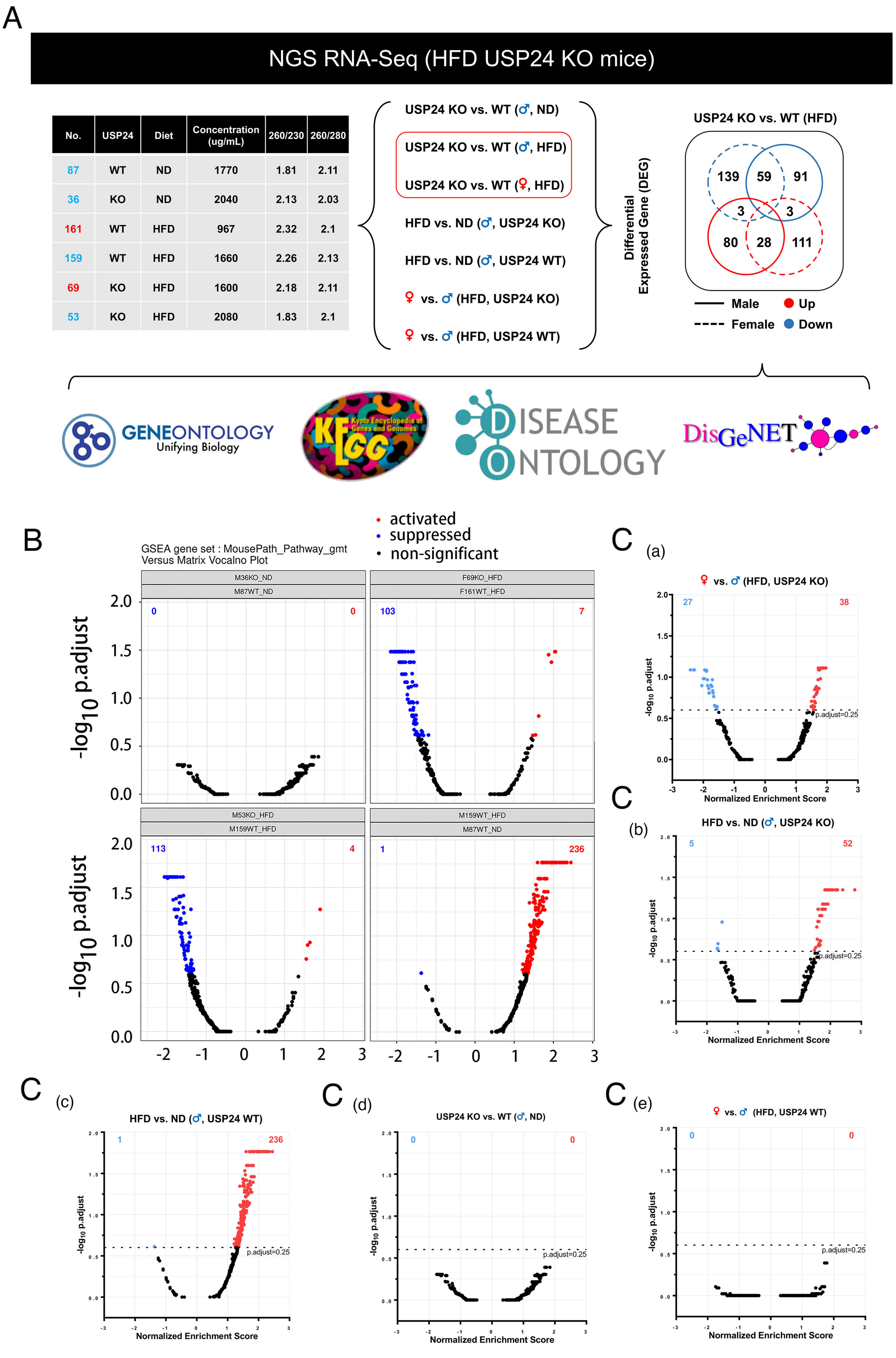

No.	USP24	Diet	Concentration (ug/mL)	260/230	260/280
87	WT	ND	1770	1.81	2.11
36	KO	ND	2040	2.13	2.03
161	WT	HFD	967	2.32	2.1
159	WT	HFD	1660	2.26	2.13
69	KO	HFD	1600	2.18	2.11
53	KO	HFD	2080	1.83	2.1

Suppl.Fig.7

Suppl. Fig.8

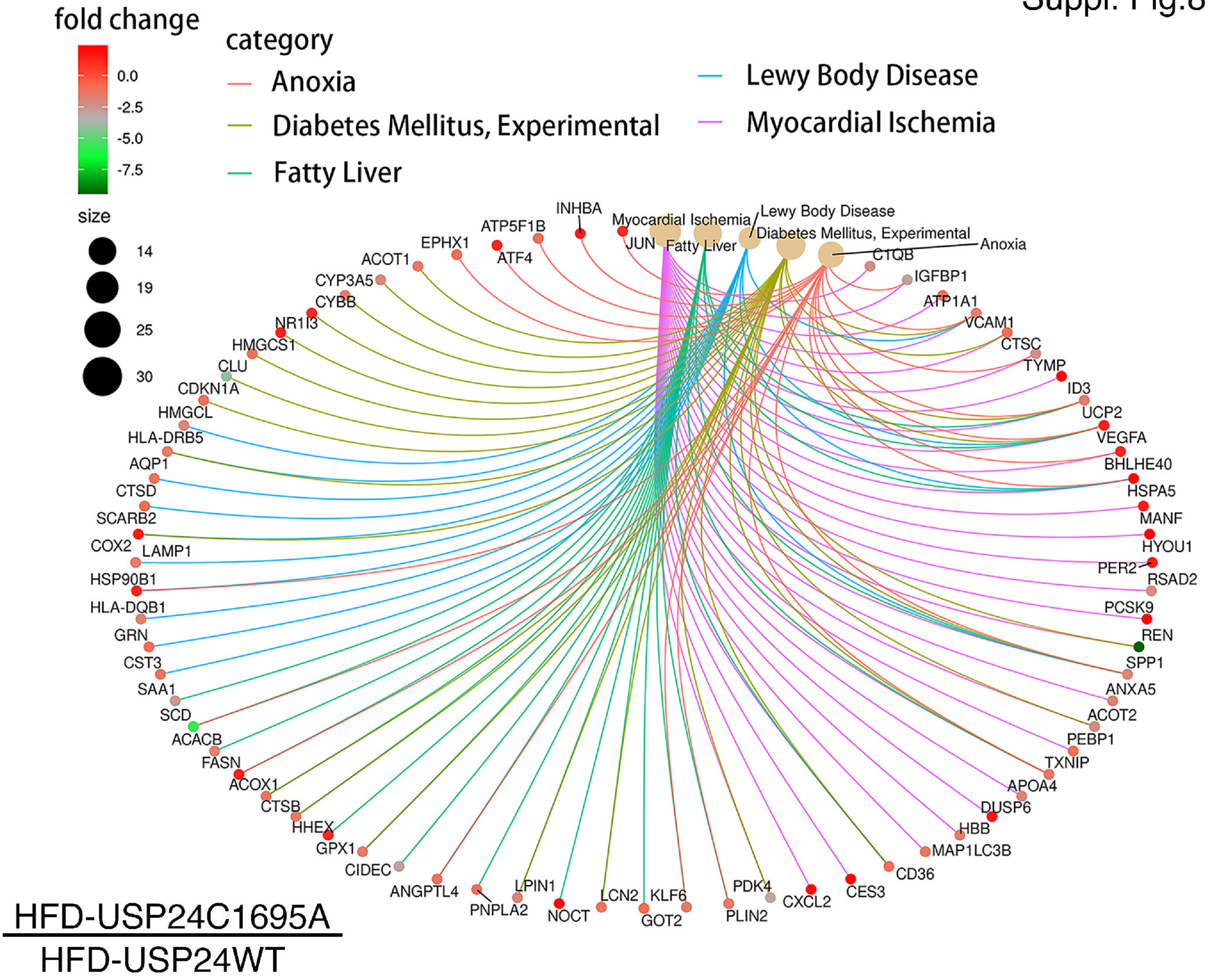
fold change
0.0
-2.5
-5.0
-7.5
size
14
19
25
30
category
Anoxia
Diabetes Mellitus, Experimental
Fatty Liver
Lewy Body Disease
Myocardial Ischemia
Myocardial Ischemia
Lewy Body Disease
Fatty Liver
Diabetes Mellitus, Experimental
Anoxia
INHBA
ATP5F1B
EPHX1
ATF4
ACOT1
JUN
CYP3A5
CYBB
NR1I3
HMGCS1
CLU
CDKN1A
HMGCL
HLA-DRB5
AQP1
CTSD
SCARB2
COX2
LAMP1
HSP90B1
HLA-DQB1
GRN
CST3
SAA1
SCD
ACACB
FASN
ACOX1
CTSB
HHEX
GPX1
CIDEC
ANGPTL4
PNPLA2
LPIN1
NOCT
LCN2
GOT2
KLF6
PLIN2
PDK4
CXCL2
CES3
CD36
MAP1LC3B
HBB
DUSP6
APOA4
TXNIP
PEBP1
ACOT2
ANXA5
SPP1
REN
PCSK9
RSAD2
PER2
HYOU1
MANF
HSPA5
BHLHE40
VEGFA
UCP2
ID3
TYMP
CTSC
VCAM1
ATP1A1
IGFBP1
C1QB
HFD-USP24C1695A
HFD-USP24WT

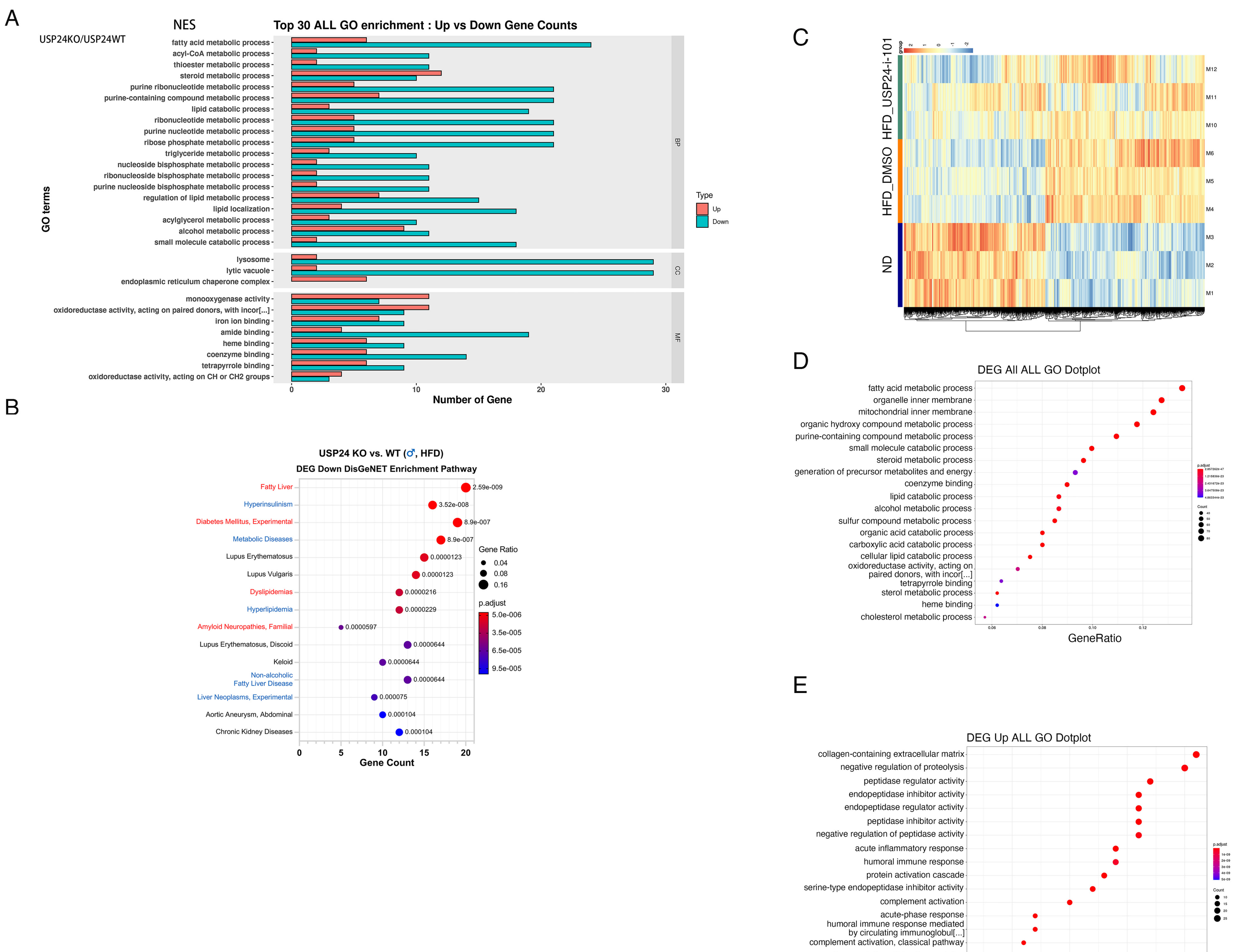
A
NES
USP24KO/USP24WT
Top 30 ALL GO enrichment : Up vs Down Gene Counts
GO terms
fatty acid metabolic process
acyl-CoA metabolic process
thioester metabolic process
steroid metabolic process
purine ribonucleotide metabolic process
purine-containing compound metabolic process
lipid catabolic process
ribonucleotide metabolic process
purine nucleotide metabolic process
ribose phosphate metabolic process
triglyceride metabolic process
nucleoside bisphosphate metabolic process
ribonucleoside bisphosphate metabolic process
purine nucleoside bisphosphate metabolic process
regulation of lipid metabolic process
lipid localization
acylglycerol metabolic process
alcohol metabolic process
small molecule catabolic process
lysosome
lytic vacuole
endoplasmic reticulum chaperone complex
monooxygenase activity
oxidoreductase activity, acting on paired donors, with incor[...]
iron ion binding
amide binding
heme binding
coenzyme binding
tetrapyrrole binding
oxidoreductase activity, acting on CH or CH2 groups
BP
CC
MF
0
10
20
30
Number of Gene
Type
Up
Down
B
USP24 KO vs. WT (♂, HFD)
DEG Down DisGeNET Enrichment Pathway
Fatty Liver
Hyperinsulinism
Diabetes Mellitus, Experimental
Metabolic Diseases
Lupus Erythematosus
Lupus Vulgaris
Dyslipidemias
Hyperlipidemia
Amyloid Neuropathies, Familial
Lupus Erythematosus, Discoid
Keloid
Non-alcoholic Fatty Liver Disease
Liver Neoplasms, Experimental
Aortic Aneurysm, Abdominal
Chronic Kidney Diseases
2.59e-009
3.52e-008
8.9e-007
8.9e-007
0.0000123
0.0000123
0.0000216
0.0000229
0.0000597
0.0000644
0.0000644
0.0000644
0.000075
0.000104
0.000104
Gene Ratio
0.04
0.08
0.16
p.adjust
5.0e-006
3.5e-005
6.5e-005
9.5e-005
0
5
10
15
20
Gene Count
C
HFD_USP24-i-101
HFD_DMSO
ND
M12
M11
M10
M6
M5
M4
M3
M2
M1
D
DEG All ALL GO Dotplot
fatty acid metabolic process
organelle inner membrane
mitochondrial inner membrane
organic hydroxy compound metabolic process
purine-containing compound metabolic process
small molecule catabolic process
steroid metabolic process
generation of precursor metabolites and energy
coenzyme binding
lipid catabolic process
alcohol metabolic process
sulfur compound metabolic process
organic acid catabolic process
carboxylic acid catabolic process
cellular lipid catabolic process
oxidoreductase activity, acting on paired donors, with incor[...]
tetrapyrrole binding
sterol metabolic process
heme binding
cholesterol metabolic process
GeneRatio
E
DEG Up ALL GO Dotplot
collagen-containing extracellular matrix
negative regulation of proteolysis
peptidase regulator activity
endopeptidase inhibitor activity
endopeptidase regulator activity
peptidase inhibitor activity
negative regulation of peptidase activity
acute inflammatory response
humoral immune response
protein activation cascade
serine-type endopeptidase inhibitor activity
complement activation
acute-phase response
humoral immune response mediated by circulating immunoglobul[...]
complement activation, classical pathway
negative regulation of blood coagulation
negative regulation of hemostasis
negative regulation of coagulation
fibrinolysis
complement activation, alternative pathway
GeneRatio

Suppl. Fig.9

Suppl. Fig.10

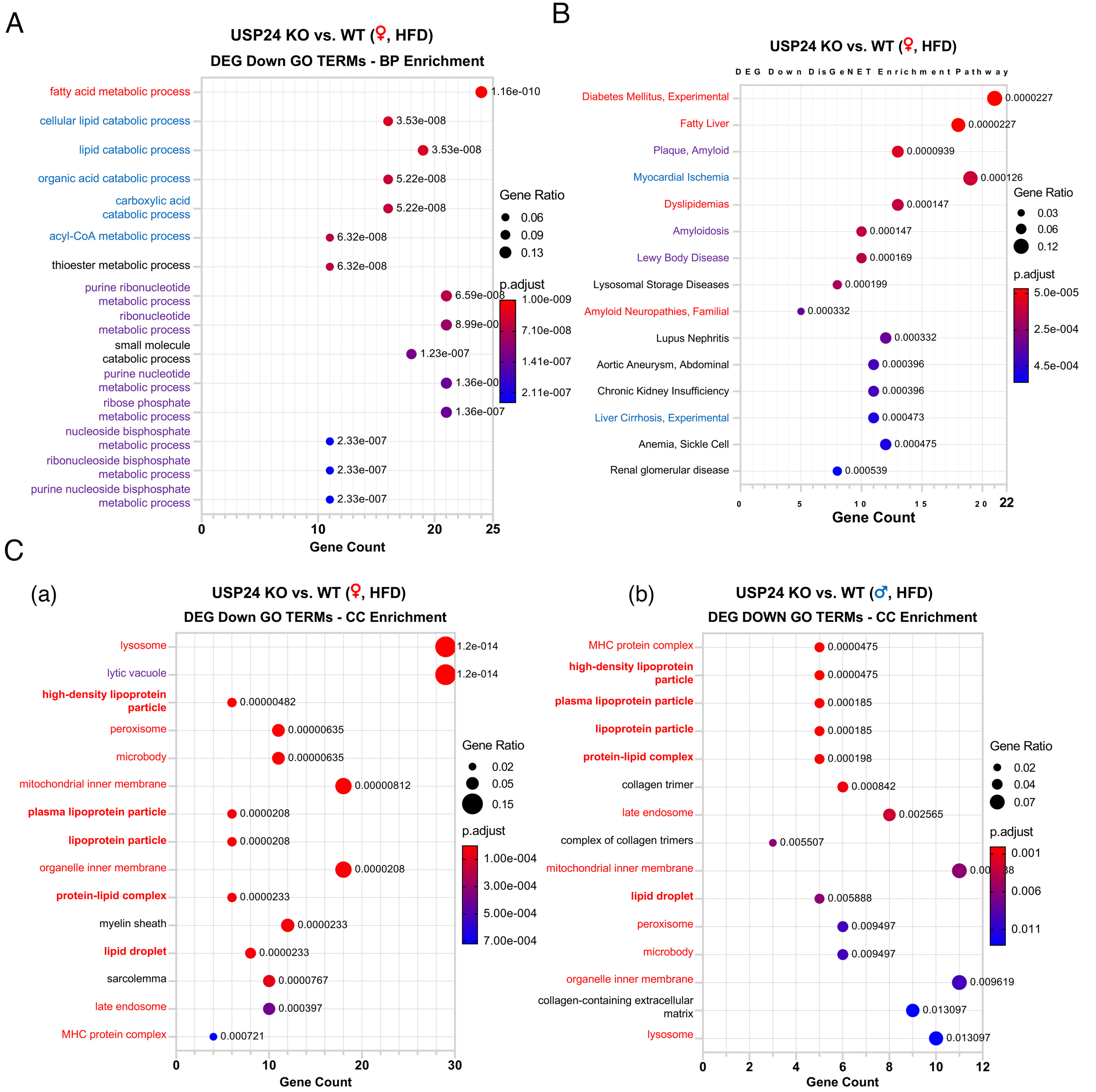
A
USP24 KO vs. WT (♀, HFD)
DEG Down GO TERMs - BP Enrichment
fatty acid metabolic process
cellular lipid catabolic process
lipid catabolic process
organic acid catabolic process
carboxylic acid catabolic process
acyl-CoA metabolic process
thioester metabolic process
purine ribonucleotide metabolic process
ribonucleotide metabolic process
small molecule catabolic process
purine nucleotide metabolic process
ribose phosphate metabolic process
nucleoside bisphosphate metabolic process
ribonucleoside bisphosphate metabolic process
purine nucleoside bisphosphate metabolic process
1.16e-010
3.53e-008
3.53e-008
5.22e-008
5.22e-008
6.32e-008
6.32e-008
6.59e-008
1.23e-007
1.36e-007
2.33e-007
2.33e-007
2.33e-007
Gene Ratio
0.06
0.09
0.13
p.adjust
1.00e-009
7.10e-008
1.41e-007
2.11e-007
0
10
20
25
Gene Count
B
USP24 KO vs. WT (♀, HFD)
DEG Down DisGeNET Enrichment Pathway
Diabetes Mellitus, Experimental
Fatty Liver
Plaque, Amyloid
Myocardial Ischemia
Dyslipidemias
Amyloidosis
Lewy Body Disease
Lysosomal Storage Diseases
Amyloid Neuropathies, Familial
Lupus Nephritis
Aortic Aneurysm, Abdominal
Chronic Kidney Insufficiency
Liver Cirrhosis, Experimental
Anemia, Sickle Cell
Renal glomerular disease
0.0000227
0.0000227
0.0000939
0.000126
0.000147
0.000147
0.000169
0.000199
0.000332
0.000332
0.000396
0.000396
0.000473
0.000475
0.000539
Gene Ratio
0.03
0.06
0.12
p.adjust
5.0e-005
2.5e-004
4.5e-004
0
5
10
15
20
22
Gene Count
C
(a)
USP24 KO vs. WT (♀, HFD)
DEG Down GO TERMs - CC Enrichment
lysosome
lytic vacuole
high-density lipoprotein particle
peroxisome
microbody
mitochondrial inner membrane
plasma lipoprotein particle
lipoprotein particle
organelle inner membrane
protein-lipid complex
myelin sheath
lipid droplet
sarcolemma
late endosome
MHC protein complex
1.2e-014
1.2e-014
0.00000482
0.00000635
0.00000635
0.00000812
0.0000208
0.0000208
0.0000208
0.0000233
0.0000233
0.0000233
0.0000767
0.000397
0.000721
Gene Ratio
0.02
0.05
0.15
p.adjust
1.00e-004
3.00e-004
5.00e-004
7.00e-004
0
10
20
30
Gene Count
(b)
USP24 KO vs. WT (♂, HFD)
DEG DOWN GO TERMs - CC Enrichment
MHC protein complex
high-density lipoprotein particle
plasma lipoprotein particle
lipoprotein particle
protein-lipid complex
collagen trimer
late endosome
complex of collagen trimers
mitochondrial inner membrane
lipid droplet
peroxisome
microbody
organelle inner membrane
collagen-containing extracellular matrix
lysosome
0.0000475
0.0000475
0.000185
0.000185
0.000198
0.000842
0.002565
0.005507
0.005888
0.009497
0.009497
0.009619
0.013097
0.013097
Gene Ratio
0.02
0.04
0.07
p.adjust
0.001
0.006
0.011
0
2
4
6
8
10
12
Gene Count

Suppl. Fig.11

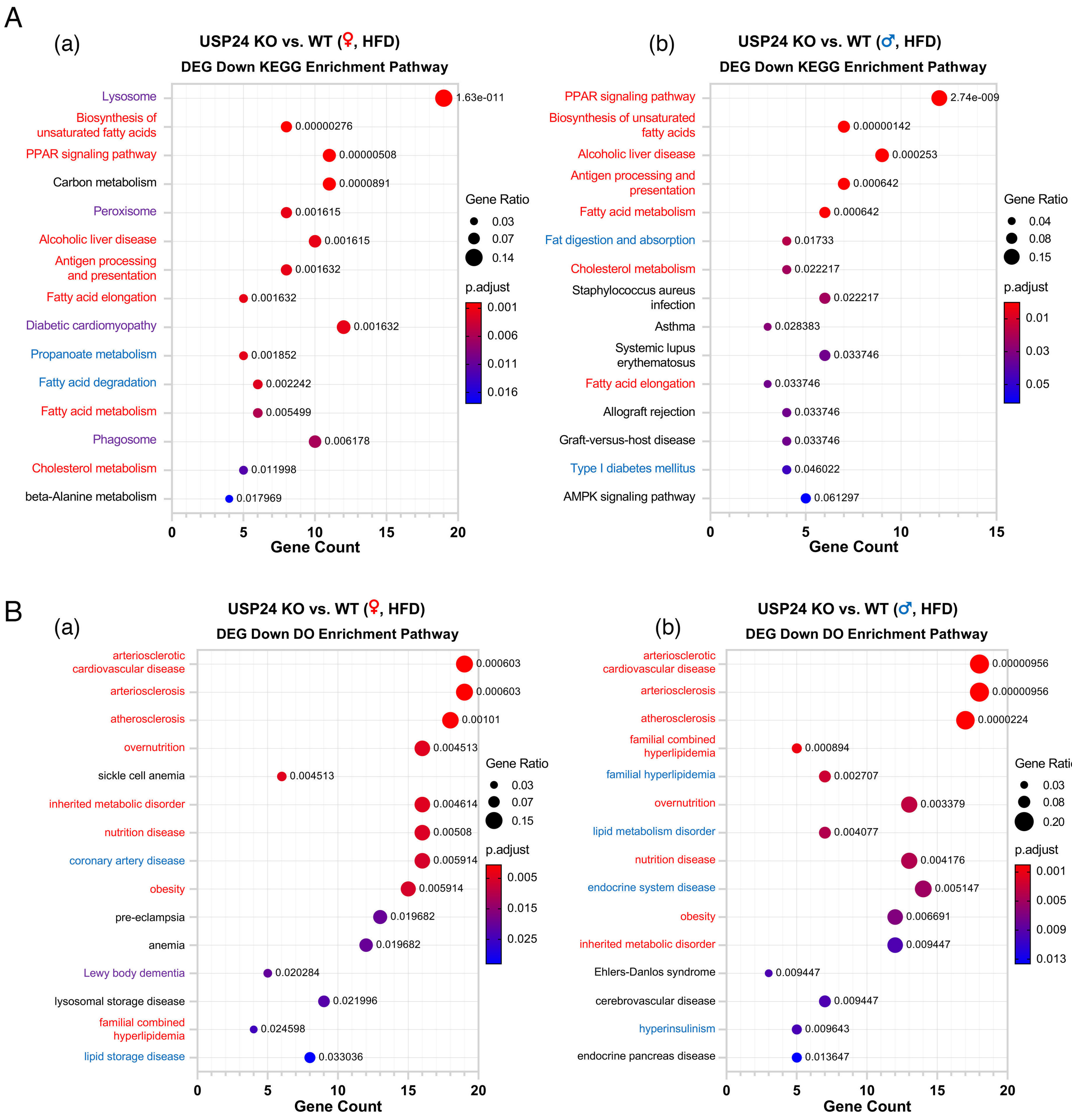
A
(a)
USP24 KO vs. WT (♀, HFD)
DEG Down KEGG Enrichment Pathway
Lysosome 1.63e-011
Biosynthesis of unsaturated fatty acids 0.00000276
PPAR signaling pathway 0.00000508
Carbon metabolism 0.0000891
Peroxisome 0.001615
Alcoholic liver disease 0.001615
Antigen processing and presentation 0.001632
Fatty acid elongation 0.001632
Diabetic cardiomyopathy 0.001632
Propanoate metabolism 0.001852
Fatty acid degradation 0.002242
Fatty acid metabolism 0.005499
Phagosome 0.006178
Cholesterol metabolism 0.011998
beta-Alanine metabolism 0.017969
Gene Count
0 5 10 15 20
Gene Ratio
0.03
0.07
0.14
p.adjust
0.001
0.006
0.011
0.016
(b)
USP24 KO vs. WT (♂, HFD)
DEG Down KEGG Enrichment Pathway
PPAR signaling pathway 2.74e-009
Biosynthesis of unsaturated fatty acids 0.00000142
Alcoholic liver disease 0.000253
Antigen processing and presentation 0.000642
Fatty acid metabolism 0.000642
Fat digestion and absorption 0.01733
Cholesterol metabolism 0.022217
Staphylococcus aureus infection 0.022217
Asthma 0.028383
Systemic lupus erythematosus 0.033746
Fatty acid elongation 0.033746
Allograft rejection 0.033746
Graft-versus-host disease 0.033746
Type I diabetes mellitus 0.046022
AMPK signaling pathway 0.061297
Gene Count
0 5 10 15
Gene Ratio
0.04
0.08
0.15
p.adjust
0.01
0.03
0.05
B
(a)
USP24 KO vs. WT (♀, HFD)
DEG Down DO Enrichment Pathway
arteriosclerotic cardiovascular disease 0.000603
arteriosclerosis 0.000603
atherosclerosis 0.00101
overnutrition 0.004513
sickle cell anemia 0.004513
inherited metabolic disorder 0.004614
nutrition disease 0.00508
coronary artery disease 0.005914
obesity 0.005914
pre-eclampsia 0.019682
anemia 0.019682
Lewy body dementia 0.020284
lysosomal storage disease 0.021996
familial combined hyperlipidemia 0.024598
lipid storage disease 0.033036
Gene Count
0 5 10 15 20
Gene Ratio
0.03
0.07
0.15
p.adjust
0.005
0.015
0.025
(b)
USP24 KO vs. WT (♂, HFD)
DEG Down DO Enrichment Pathway
arteriosclerotic cardiovascular disease 0.00000956
arteriosclerosis 0.00000956
atherosclerosis 0.0000224
familial combined hyperlipidemia 0.000894
familial hyperlipidemia 0.002707
overnutrition 0.003379
lipid metabolism disorder 0.004077
nutrition disease 0.004176
endocrine system disease 0.005147
obesity 0.006691
inherited metabolic disorder 0.009447
Ehlers-Danlos syndrome 0.009447
cerebrovascular disease 0.009447
hyperinsulinism 0.009643
endocrine pancreas disease 0.013647
Gene Count
0 5 10 15 20
Gene Ratio
0.03
0.08
0.20
p.adjust
0.001
0.005
0.009
0.013

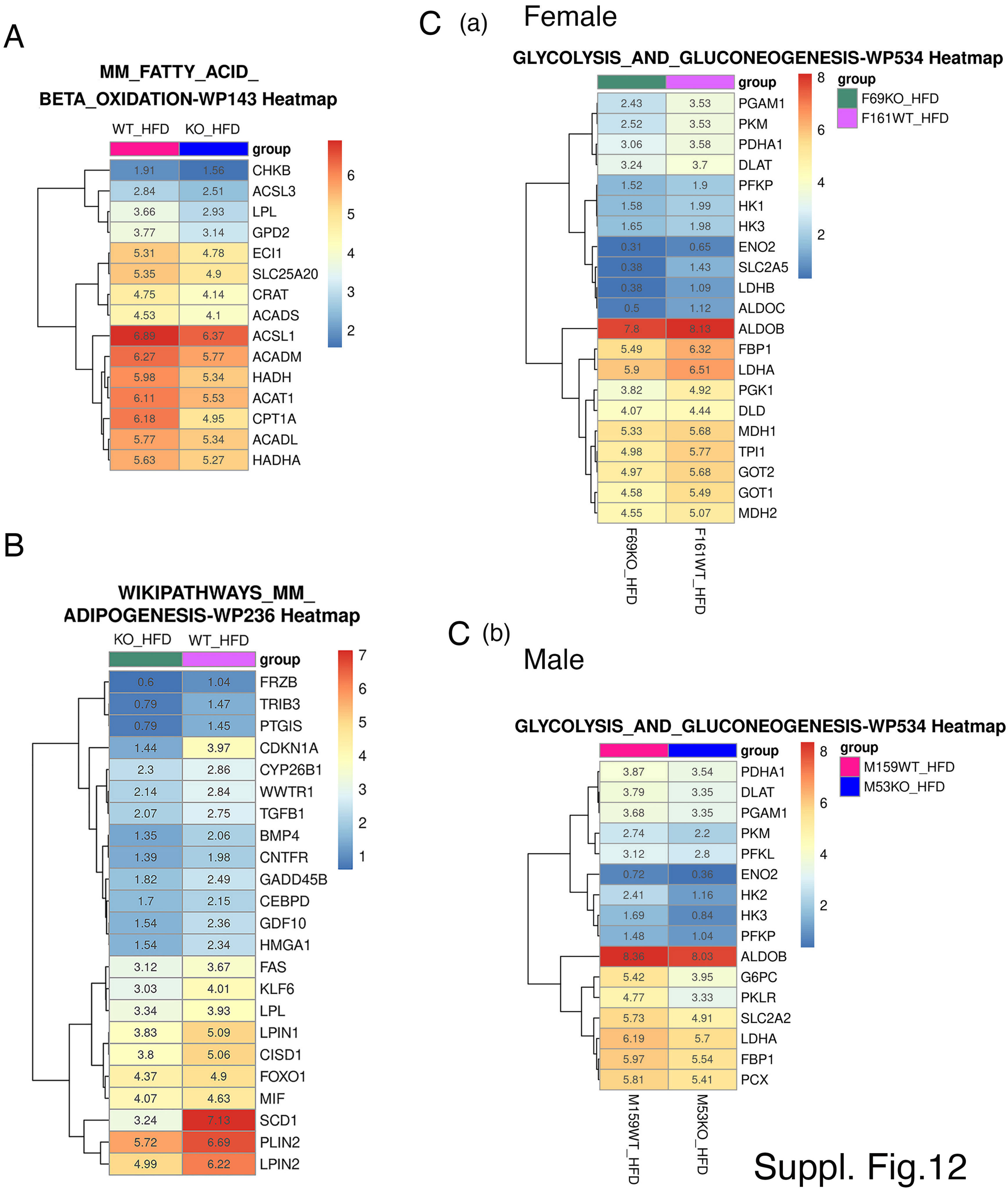
A
MM_FATTY_ACID_
BETA_OXIDATION-WP143 Heatmap
WT_HFD KO_HFD
group
1.91 1.56 CHKB
2.84 2.51 ACSL3
3.66 2.93 LPL
3.77 3.14 GPD2
5.31 4.78 ECI1
5.35 4.9 SLC25A20
4.75 4.14 CRAT
4.53 4.1 ACADS
6.89 6.37 ACSL1
6.27 5.77 ACADM
5.98 5.34 HADH
6.11 5.53 ACAT1
6.18 4.95 CPT1A
5.77 5.34 ACADL
5.63 5.27 HADHA
6
5
4
3
2
B
WIKIPATHWAYS_MM_
ADIPOGENESIS-WP236 Heatmap
KO_HFD WT_HFD
group
0.6 1.04 FRZB
0.79 1.47 TRIB3
0.79 1.45 PTGIS
1.44 3.97 CDKN1A
2.3 2.86 CYP26B1
2.14 2.84 WWTR1
2.07 2.75 TGFB1
1.35 2.06 BMP4
1.39 1.98 CNTFR
1.82 2.49 GADD45B
1.7 2.15 CEBPD
1.54 2.36 GDF10
1.54 2.34 HMGA1
3.12 3.67 FAS
3.03 4.01 KLF6
3.34 3.93 LPL
3.83 5.09 LPIN1
3.8 5.06 CISD1
4.37 4.9 FOXO1
4.07 4.63 MIF
3.24 7.13 SCD1
5.72 6.69 PLIN2
4.99 6.22 LPIN2
7
6
5
4
3
2
1
C (a) Female
GLYCOLYSIS_AND_GLUCONEOGENESIS-WP534 Heatmap
group
2.43 3.53 PGAM1
2.52 3.53 PKM
3.06 3.58 PDHA1
3.24 3.7 DLAT
1.52 1.9 PFKP
1.58 1.99 HK1
1.65 1.98 HK3
0.31 0.65 ENO2
0.38 1.43 SLC2A5
0.38 1.09 LDHB
0.5 1.12 ALDOC
7.8 8.13 ALDOB
5.49 6.32 FBP1
5.9 6.51 LDHA
3.82 4.92 PGK1
4.07 4.44 DLD
5.33 5.68 MDH1
4.98 5.77 TPI1
4.97 5.68 GOT2
4.58 5.49 GOT1
4.55 5.07 MDH2
F69KO_HFD
F161WT_HFD
8
6
4
2
group
F69KO_HFD
F161WT_HFD
C (b) Male
GLYCOLYSIS_AND_GLUCONEOGENESIS-WP534 Heatmap
group
3.87 3.54 PDHA1
3.79 3.35 DLAT
3.68 3.35 PGAM1
2.74 2.2 PKM
3.12 2.8 PFKL
0.72 0.36 ENO2
2.41 1.16 HK2
1.69 0.84 HK3
1.48 1.04 PFKP
8.36 8.03 ALDOB
5.42 3.95 G6PC
4.77 3.33 PKLR
5.73 4.91 SLC2A2
6.19 5.7 LDHA
5.97 5.54 FBP1
5.81 5.41 PCX
M159WT_HFD
M53KO_HFD
8
6
4
2
group
M159WT_HFD
M53KO_HFD

Suppl. Fig.12

A

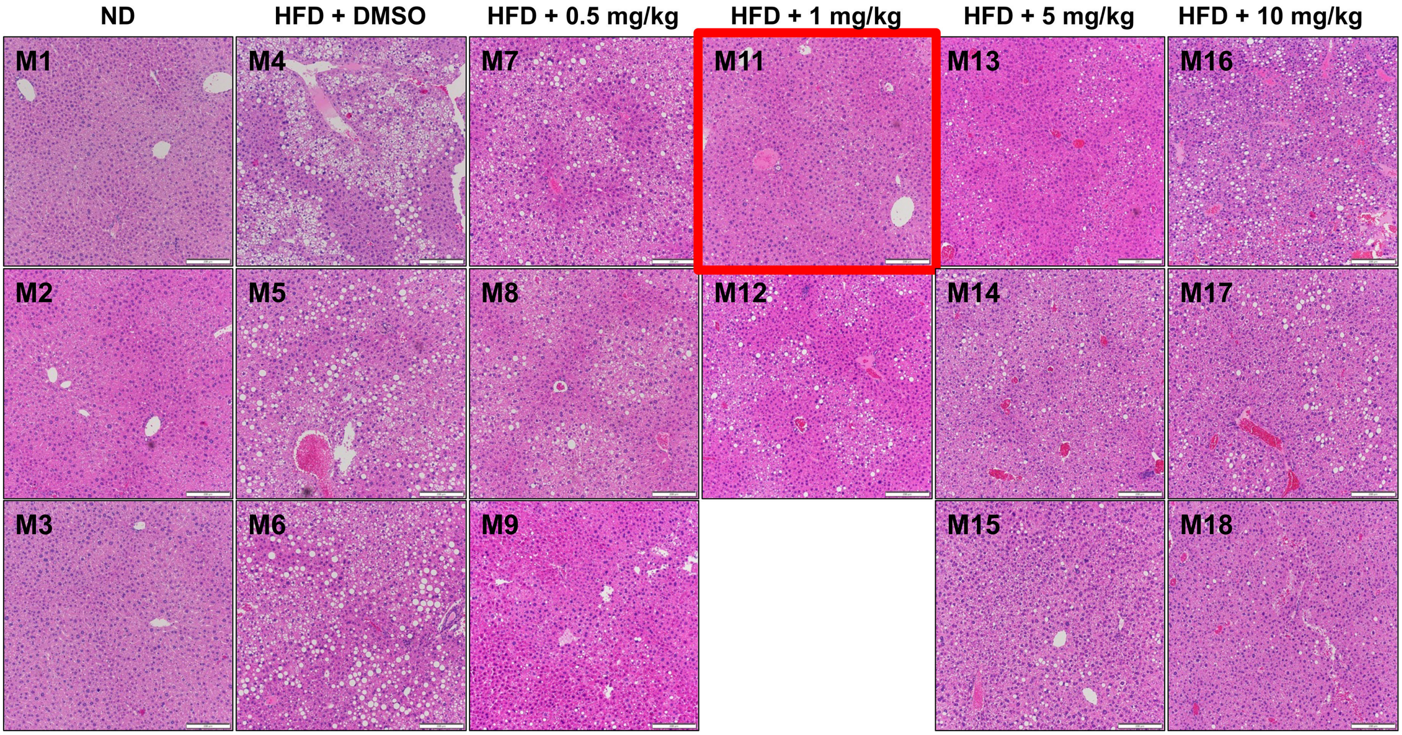

B

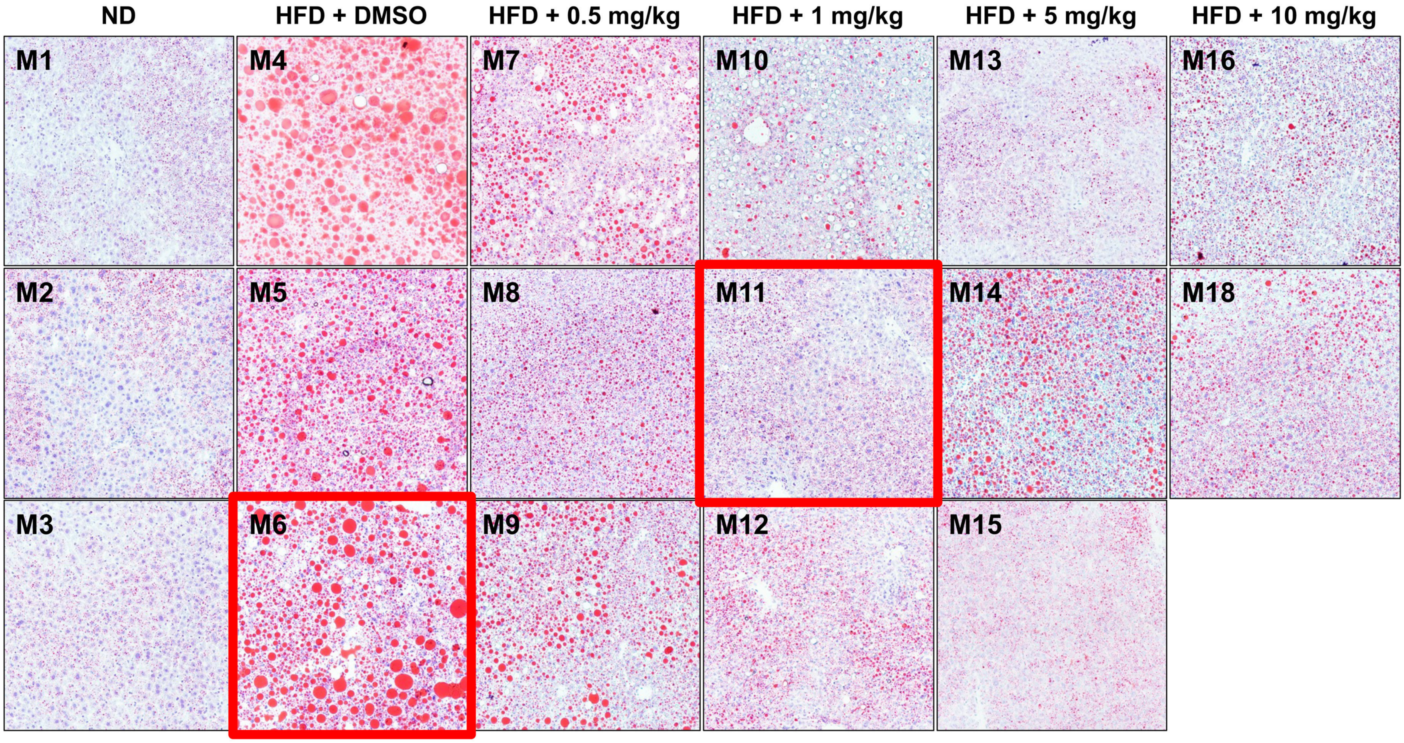

Suppl. Fig.13

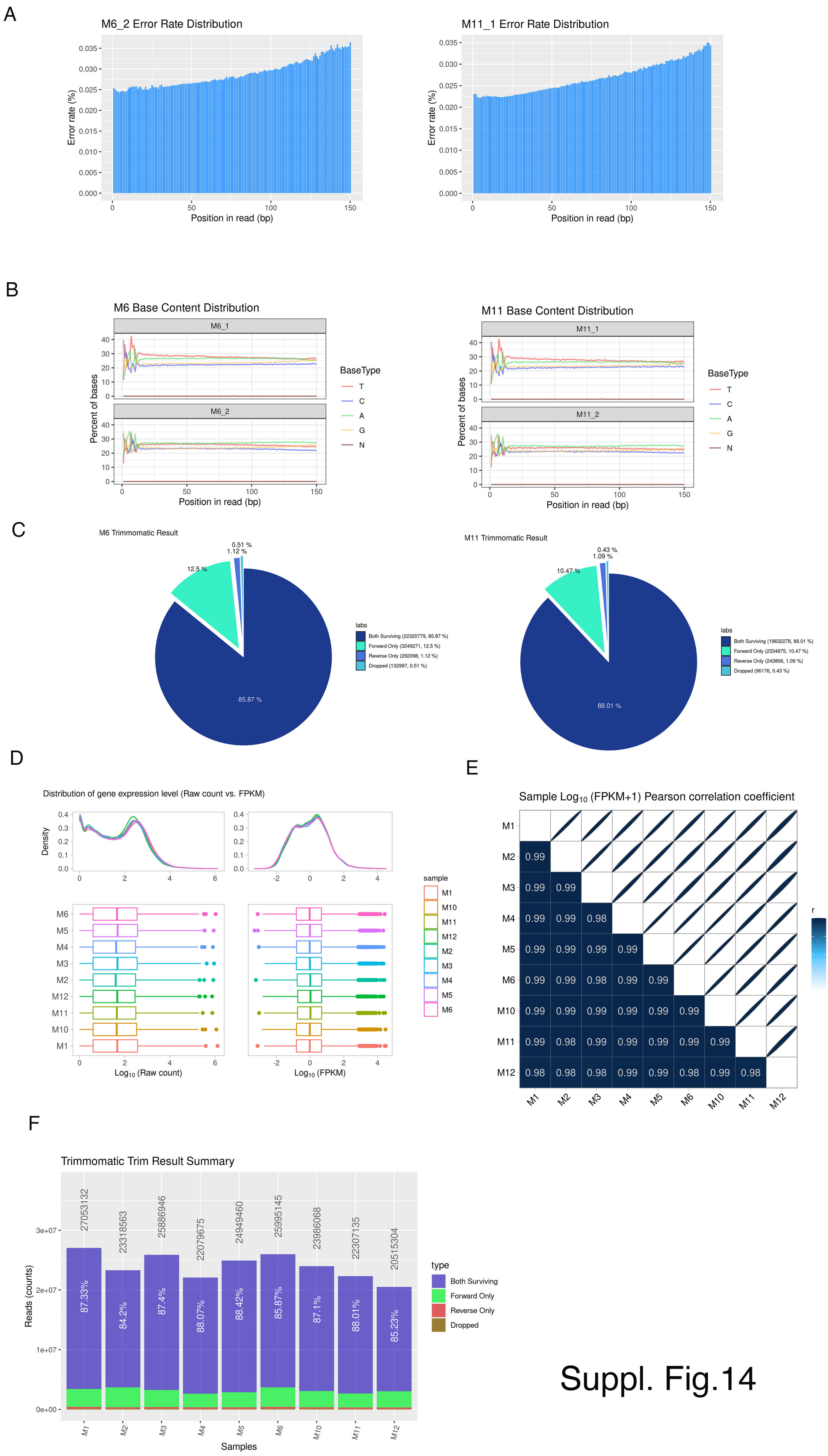
A
M6_2 Error Rate Distribution
M11_1 Error Rate Distribution
Error rate (%)
Position in read (bp)
B
M6 Base Content Distribution
M11 Base Content Distribution
M6_1
M6_2
M11_1
M11_2
Percent of bases
Position in read (bp)
BaseType
T
C
A
G
N
C
M6 Trimmomatic Result
M11 Trimmomatic Result
labs
Both Surviving (22320779, 85.87 %)
Forward Only (3249271, 12.5 %)
Reverse Only (292098, 1.12 %)
Dropped (132997, 0.51 %)
Both Surviving (19632278, 88.01 %)
Forward Only (2334875, 10.47 %)
Reverse Only (243806, 1.09 %)
Dropped (96176, 0.43 %)
D
Distribution of gene expression level (Raw count vs. FPKM)
Density
Log10 (Raw count)
Log10 (FPKM)
sample
E
Sample Log10 (FPKM+1) Pearson correlation coefficient
r
F
Trimmomatic Trim Result Summary
Reads (counts)
Samples
type
Both Surviving
Forward Only
Reverse Only
Dropped
27053132
23318563
25886946
22079675
24949460
25995145
23986068
22307135
20515304
87.33%
84.2%
87.4%
88.07%
88.42%
85.87%
87.1%
88.01%
85.23%

Suppl. Fig.14

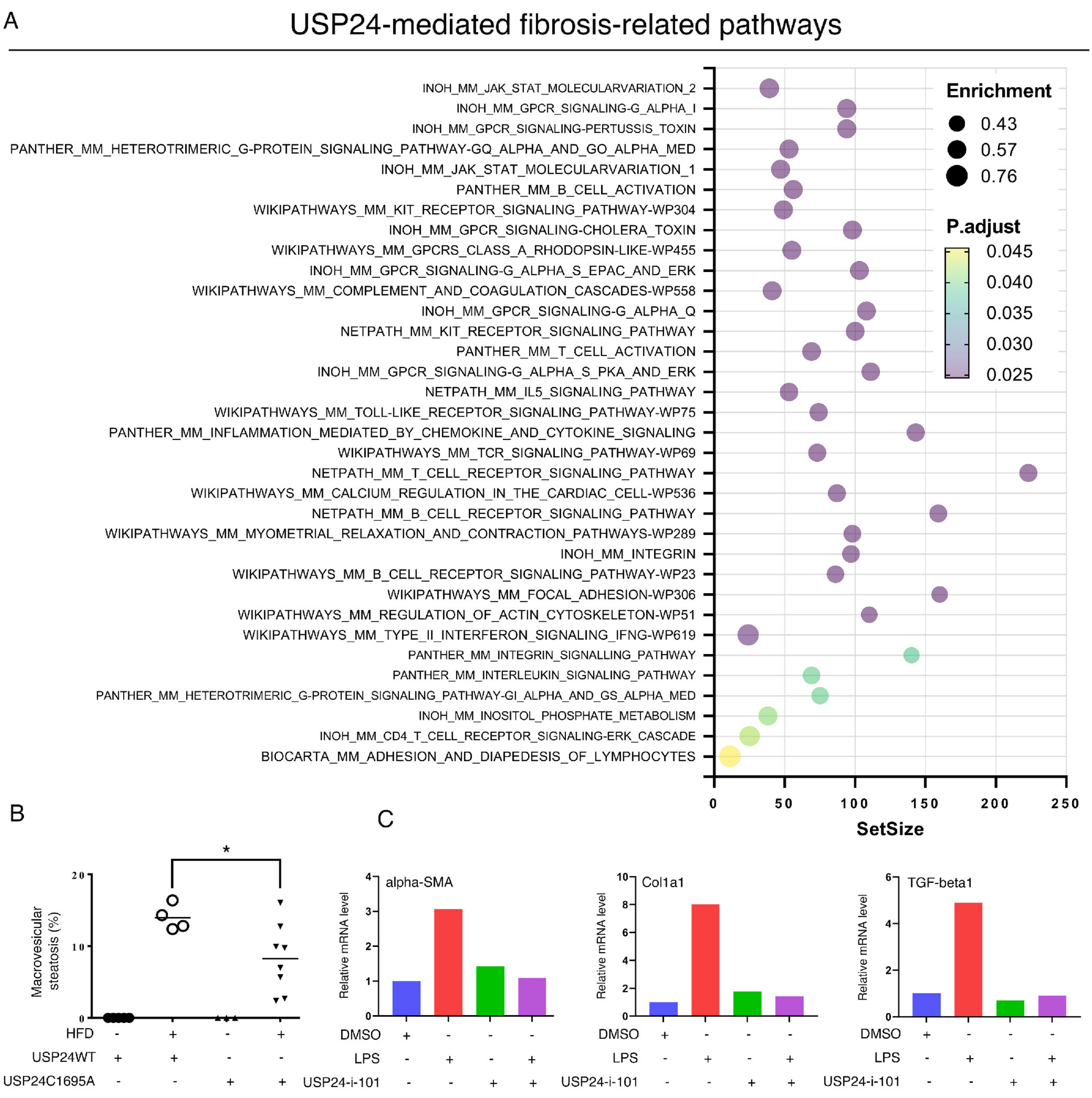
A
USP24-mediated fibrosis-related pathways
INOH_MM_JAK_STAT_MOLECULARVARIATION_2
INOH_MM_GPCR_SIGNALING-G_ALPHA_I
INOH_MM_GPCR_SIGNALING-PERTUSSIS_TOXIN
PANTHER_MM_HETEROTRIMERIC_G-PROTEIN_SIGNALING_PATHWAY-GQ_ALPHA_AND_GO_ALPHA_MED
INOH_MM_JAK_STAT_MOLECULARVARIATION_1
PANTHER_MM_B_CELL_ACTIVATION
WIKIPATHWAYS_MM_KIT_RECEPTOR_SIGNALING_PATHWAY-WP304
INOH_MM_GPCR_SIGNALING-CHOLERA_TOXIN
WIKIPATHWAYS_MM_GPCRS_CLASS_A_RHODOPSIN-LIKE-WP455
INOH_MM_GPCR_SIGNALING-G_ALPHA_S_EPAC_AND_ERK
WIKIPATHWAYS_MM_COMPLEMENT_AND_COAGULATION_CASCADES-WP558
INOH_MM_GPCR_SIGNALING-G_ALPHA_Q
NETPATH_MM_KIT_RECEPTOR_SIGNALING_PATHWAY
PANTHER_MM_T_CELL_ACTIVATION
INOH_MM_GPCR_SIGNALING-G_ALPHA_S_PKA_AND_ERK
NETPATH_MM_IL5_SIGNALING_PATHWAY
WIKIPATHWAYS_MM_TOLL-LIKE_RECEPTOR_SIGNALING_PATHWAY-WP75
PANTHER_MM_INFLAMMATION_MEDIATED_BY_CHEMOKINE_AND_CYTOKINE_SIGNALING
WIKIPATHWAYS_MM_TCR_SIGNALING_PATHWAY-WP69
NETPATH_MM_T_CELL_RECEPTOR_SIGNALING_PATHWAY
WIKIPATHWAYS_MM_CALCIUM_REGULATION_IN_THE_CARDIAC_CELL-WP536
NETPATH_MM_B_CELL_RECEPTOR_SIGNALING_PATHWAY
WIKIPATHWAYS_MM_MYOMETRIAL_RELAXATION_AND_CONTRACTION_PATHWAYS-WP289
INOH_MM_INTEGRIN
WIKIPATHWAYS_MM_B_CELL_RECEPTOR_SIGNALING_PATHWAY-WP23
WIKIPATHWAYS_MM_FOCAL_ADHESION-WP306
WIKIPATHWAYS_MM_REGULATION_OF_ACTIN_CYTOSKELETON-WP51
WIKIPATHWAYS_MM_TYPE_II_INTERFERON_SIGNALING_IFNG-WP619
PANTHER_MM_INTEGRIN_SIGNALLING_PATHWAY
PANTHER_MM_INTERLEUKIN_SIGNALING_PATHWAY
PANTHER_MM_HETEROTRIMERIC_G-PROTEIN_SIGNALING_PATHWAY-GI_ALPHA_AND_GS_ALPHA_MED
INOH_MM_INOSITOL_PHOSPHATE_METABOLISM
INOH_MM_CD4_T_CELL_RECEPTOR_SIGNALING-ERK_CASCADE
BIOCARTA_MM_ADHESION_AND_DIAPEDESIS_OF_LYMPHOCYTES
Enrichment
0.43
0.57
0.76
P.adjust
0.045
0.040
0.035
0.030
0.025
0
50
100
150
200
250
SetSize
B
Macrovesicular steatosis (%)
0
10
20
*
HFD - + - +
USP24WT + + - -
USP24C1695A - - + +
C
alpha-SMA
Relative mRNA level
0
1
2
3
4
DMSO + - - -
LPS - + - +
USP24-i-101 - - + +
Col1a1
Relative mRNA level
0
2
4
6
8
10
DMSO + - - -
LPS - + - +
USP24-i-101 - - + +
TGF-beta1
Relative mRNA level
0
2
4
6
DMSO + - - -
LPS - + - +
USP24-i-101 - - + +

Suppl. Fig.15

Macrovesicular steatosis

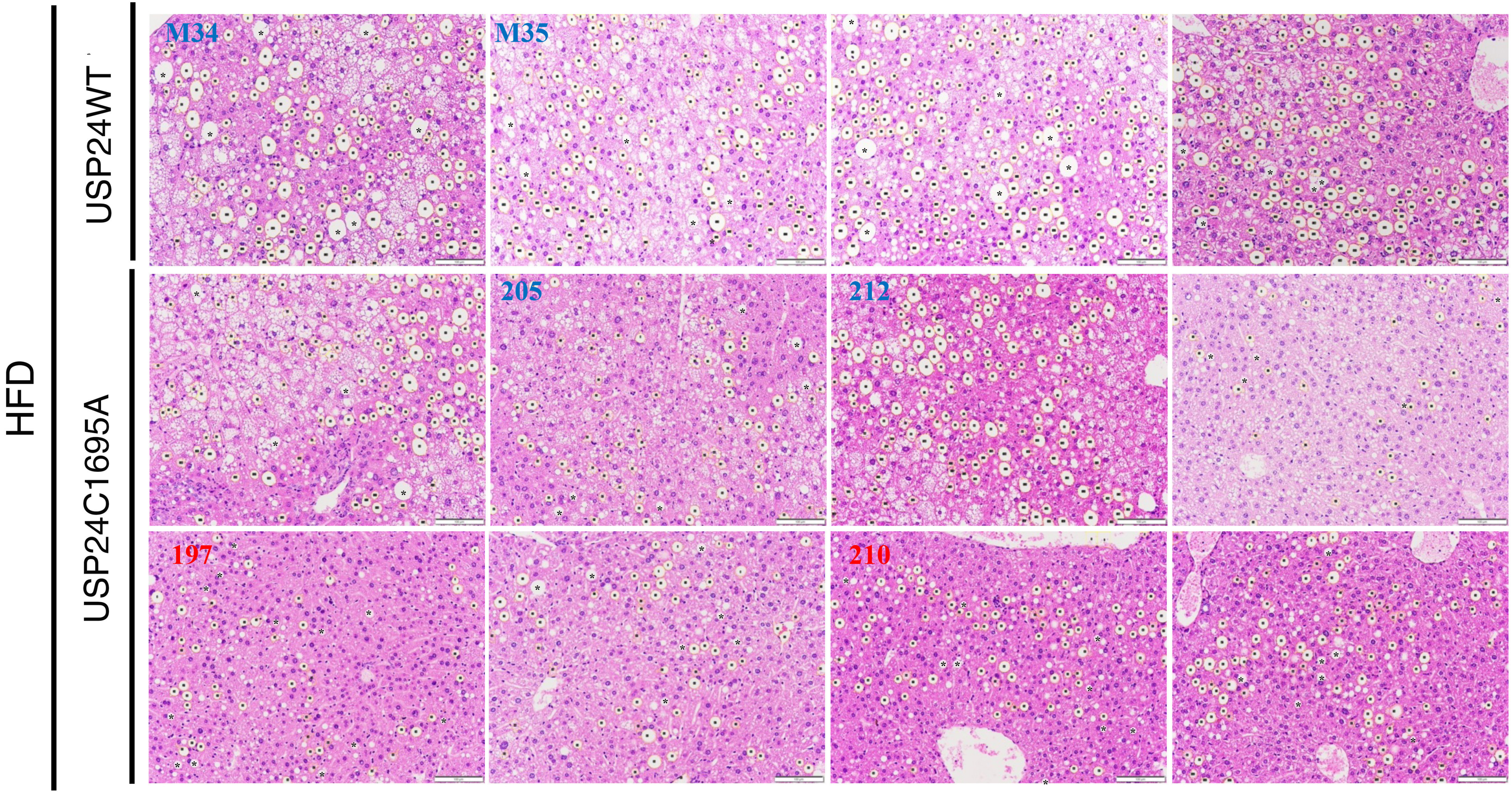

NASH: 1&2

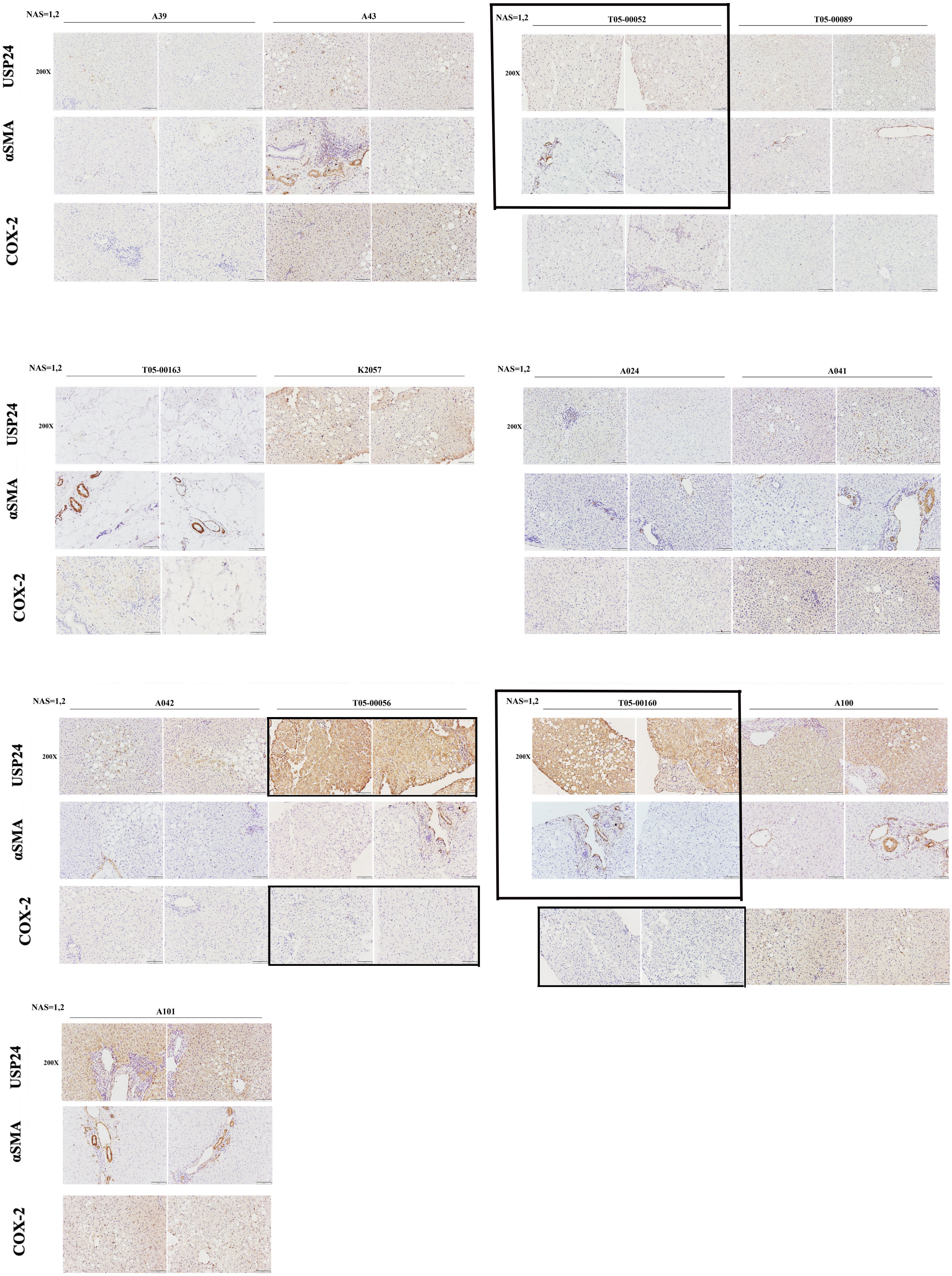

Suppl. Fig.17-1

NASH: 3&4

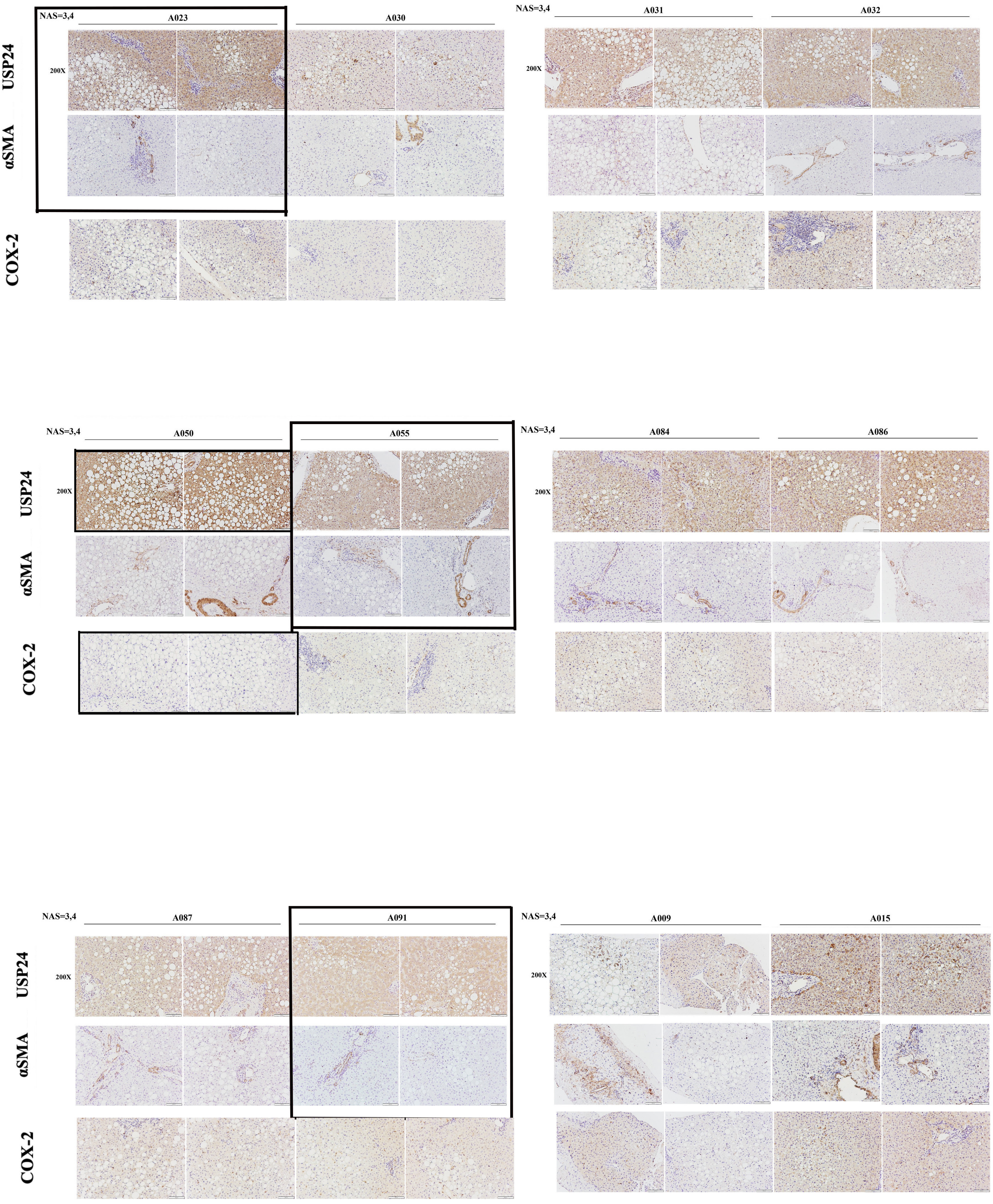

Suppl. Fig.17-2

NASH: 5&6

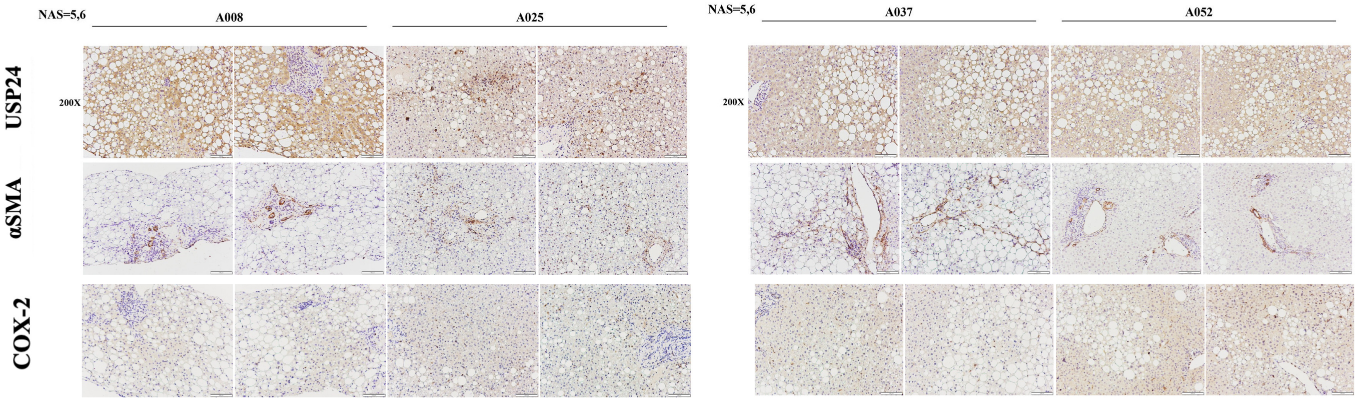

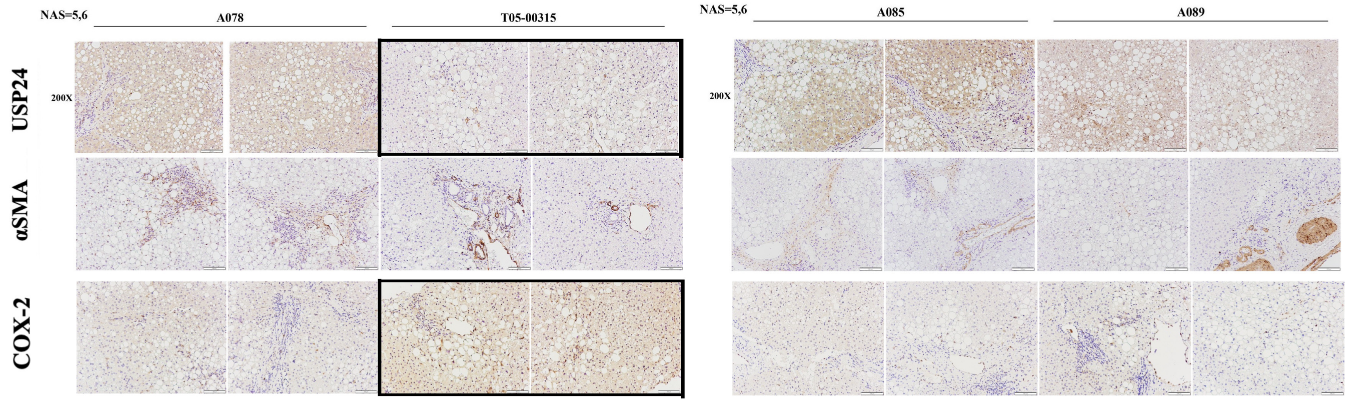

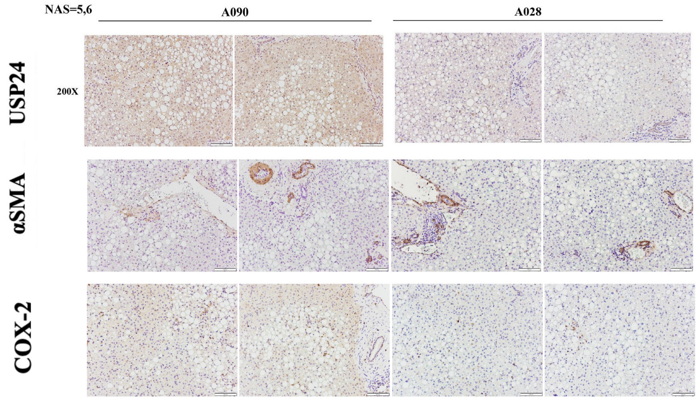

NASH: 7&8

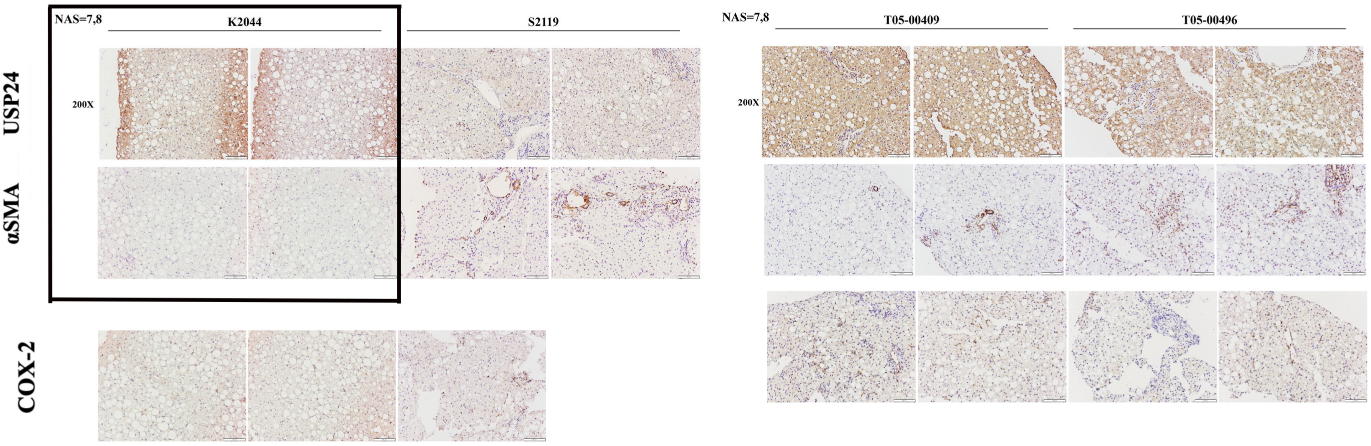

Suppl. Fig.17-3

Suppl. Fig.18

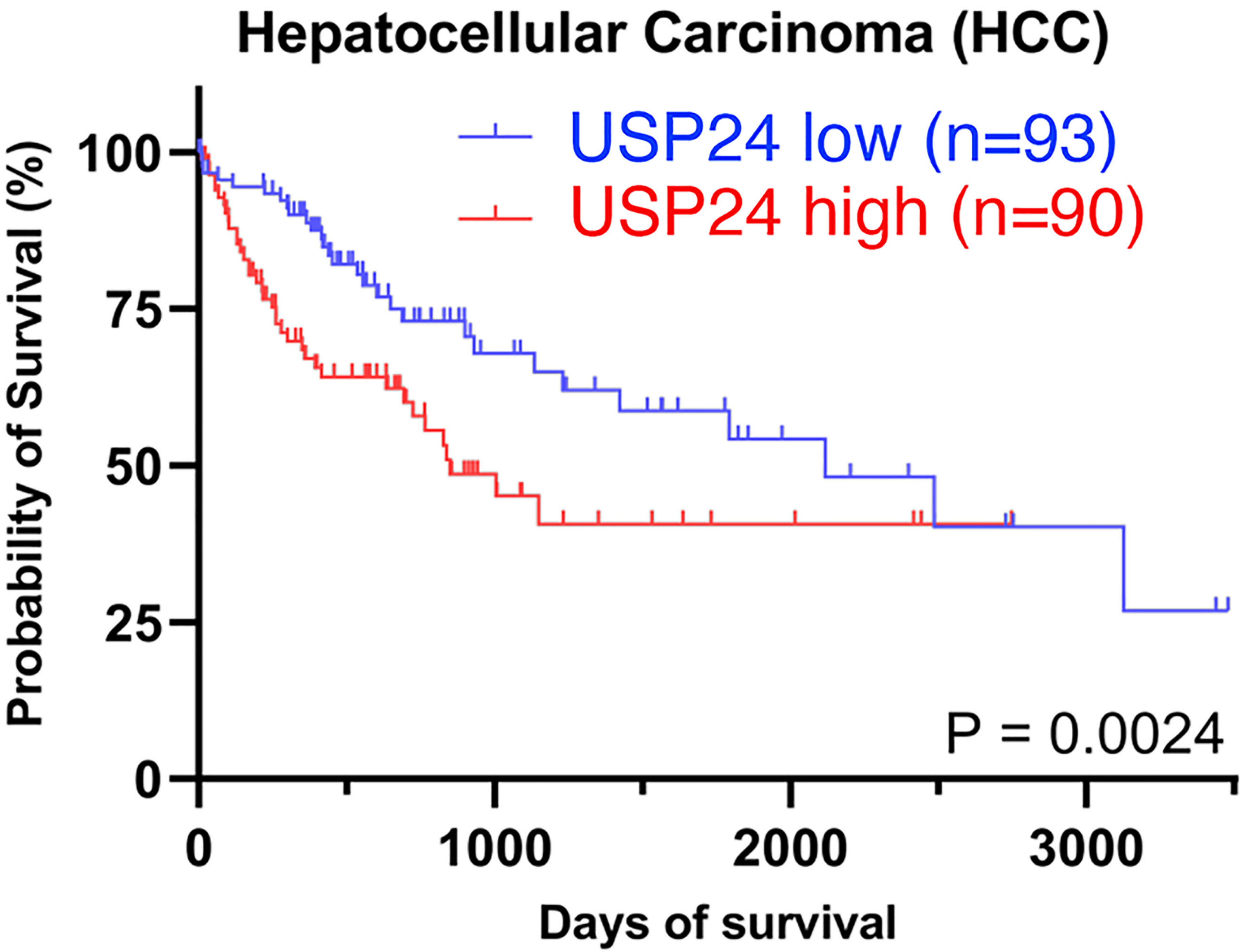

Hepatocellular Carcinoma (HCC)
USP24 low (n=93)
USP24 high (n=90)
Probability of Survival (%)
100
75
50
25
0
P = 0.0024
0
1000
2000
3000
Days of survival
